# Supplementary material for: Exploiting negative photochromism to harness a four-photon-like fluorescence response with two-photon excitation
Source: Nat Commun. 2025 Dec 3;16:10897. doi: 10.1038/s41467-025-66602-1 (PMC12678773; doi:10.1038/s41467-025-66602-1)
Supplement: Supplementary file 1 — Supplementary Information [file 41467_2025_66602_MOESM1_ESM.pdf]

Supplementary Information

Exploiting negative photochromism to harness a four-photon-like fluorescence response with two-photon excitation

Carlos Benitez-Martin,[1,2] Jean Rouillon,[1] Eduard Fron,[3,4] Flip de Jong,[3,4] Morten Grøtli,[2]* Johan Hofkens,[3,4]* Uwe Pischel,[5]* Joakim Andréasson[1]*

[1] *Department of Chemistry and Chemical Engineering, Chemistry and Biochemistry, Chalmers University of Technology, 41296 Göteborg, Sweden*

[2] *Department of Chemistry and Molecular Biology, University of Gothenburg, 40530 Göteborg, Sweden*

[3] *Chem&Tech – Molecular Imaging and Photonics, KU Leuven, Celestijnenlaan 200F, 3001 Leuven, Belgium*

[4] *KU Leuven Core Facility for Advanced Spectroscopy, Celestijnenlaan 200F, 3001 Leuven, Belgium*

[5] *CIQSO - Center for Research in Sustainable Chemistry and Department of Chemistry, University of Huelva, 21071 Huelva, Spain*

Corresponding authors

Prof. Dr. Morten Grøtli

Email: grotli@chem.gu.se
Postal address: Department of Chemistry and Chemical Engineering, Chemistry and Biochemistry, Chalmers University of Technology, SE-41296 Göteborg, Sweden

Prof. Dr. Johan Hofkens

Email: johan.hofkens@kuleuven.be
Postal address: Department of Chemistry, Molecular Imaging and Photonics, KU Leuven, Celestijnenlaan 200F, 3001 Leuven, Belgium

Prof. Dr. Uwe Pischel

Email: uwe.pischel@diq.uhu.es
Postal address: CIQSO - Center for Research in Sustainable Chemistry and Department of Chemistry, University of Huelva, E-21071 Huelva, Spain

Prof. Dr. Joakim Andréasson

Email: a-son@chalmers.se
Postal address: Department of Chemistry and Chemical Engineering, Chemistry and Biochemistry, Chalmers University of Technology, SE-412 96 Gothenburg, Sweden

Contents of Supplementary Information

1. Synthetic details 1

- Synthesis of **DASA/FRT** and characterization data 1
- Synthesis of **DASA/Napht** and characterization data 4
- Synthesis of **NOMe/Napht** and characterization data 6
- Synthesis of ***asy*NOMe/Napht** and characterization data 9
- Synthesis of **NOMe/Phtha** and characterization data 15
- Synthesis of **NTPA/Napht** and characterization data 20
- Synthesis of other model compounds 26

2. Optical spectroscopy 29

- UV/vis and fluorescence spectra 29
- Time-correlated single-photon counting data of model compounds 31
- Two-photon absorption characterization of model compounds 32
- Fatigue resistance studies 33

3. Theoretical calculations 35

- Optimized structures at the ground state 35
- Additional TDDFT calculations 38

4. FRET experimental characterization and two-photon validation studies 42

- fs-UC studies for FRET characterization 42
- Background emission determination 44
- Time-dependent fluorescence detection under 2P-excitation and corresponding log-log plots 44

5. NMR spectra 46

6. Supplementary Notes 91

7. Supplementary References 92

1. Synthetic details

- ## Synthesis of DASA/FRT and characterization data

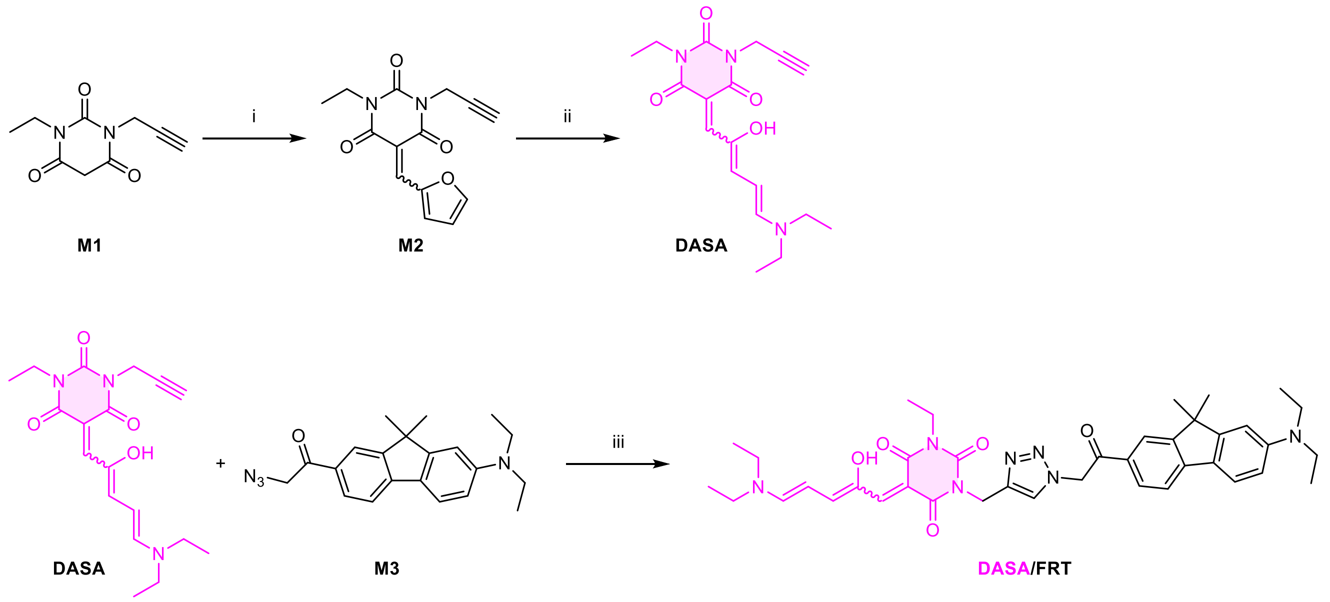

Figure S1. Synthesis of **DASA/FRT**. *Reagents and conditions*: (i) furfuraldehyde, H_2O, rt, 0.5 h, **84%**; (ii) diethylamine, THF, rt, 10 min, **85%**; (iii) $[(CH_3CN)_4Cu)]PF_6$, $CHCl_3$, rt, 4 h, **18%**.

M1 – 1-ethyl-3-(prop-2-yn-1-yl)pyrimidine-2,4,6(1*H*,3*H*,5*H*)-trione

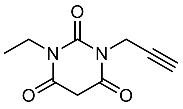

M1

M1 was prepared following a one-pot procedure reported in reference 1, without any changes. More concretely, propargyl amine (5 mmol) was added to the ethyl isocyanate (5 mmol) in dry dichloromethane (15 ml), and the mixture was stirred at room temperature for 1-2 h. After dilution with dry dichloromethane, malonyl chloride (1.1 mmol) was added dropwise under stirring at room temperature for 5 min. The resulting pale-yellow solution was stirred for an additional 1 h. The reaction was followed by TLC. After the urea intermediate was fully consumed, the reaction mixture was concentrated at reduced pressure to a small volume and purified by column chromatography (pentane/EtOAc 9:1 to 1:4). **M1** was isolated as a pale yellow solid. All characterization data were fully consistent with the literature.[1]

^{1}H NMR (400 MHz, $CDCl_3$): δ 4.64 (2H, d, *J* = 2.4 Hz), 3.97 (2H, qt, *J* = 7.1 Hz), 3.71 (2H, s), 2.23 (1H, t, *J* = 2.3 Hz), 1.23 (3H, t, *J* = 7.1 Hz). (*From reference 1*).

M2 – 1-ethyl-5-(furan-2-ylmethylene)-3-(prop-2-yn-1-yl)pyrimidine-2,4,6(1*H*,3*H*,5*H*)-trione

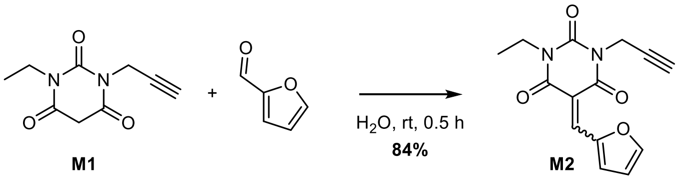

M1 (1.19 g, 6.15 mmol) and furfuraldehyde (560 µL, 6.76 mmol) were sequentially added to 20 mL of distilled H_2O, rapidly forming a yellow solid in suspension. The heterogeneous mixture was stirred at room temperature for 0.5 h, with reaction completion confirmed by TLC. The yellow precipitated was collected by vacuum filtration and washed with cold H_2O. The solid collected was then dissolved in DCM and washed sequentially with saturated $NaHSO_3$, H_2O,

saturated $NaHCO_3$ and brine. The organic layer was dried over $MgSO_4$, filtered, and the solvent evaporated to give **M2** as a yellow solid (1.41 g, 84% yield).

^{1}H NMR (400 MHz, $CDCl_3$): δ 8.73 – 8.64 (m, 1H), 8.47 (s, 1H), 7.87 (d, *J* = 1.6 Hz, 1H), 6.78 – 6.72 (m, 1H), 4.80 – 4.74 (m, 2H), 4.07 (q, *J* = 7.1 Hz, 2H), 2.26 – 2.20 (m, 1H), 1.32 – 1.23 (m, 3H).

^{13}C NMR (201 MHz, $CDCl_3$): δ 161.9, 161.7, 160.4, 160.0, 151.3, 151.3, 150.8, 150.2, 150.1, 141.8, 141.6, 128.8, 128.7, 115.4, 115.4, 111.4, 111.3, 78.3, 78.2, 71.3, 71.2, 38.0, 37.3, 31.6, 30.9, 13.4, 13.4.

HRMS (m/z, ESI): $[M]^+$ calculated for $C_{14}H_{12}N_2O_4$: 272.07971, found: 272.07960; Δ = -0.40 ppm.

DASA – 5-((4*E*)-5-(diethylamino)-2-hydroxypenta-2,4-dien-1-ylidene)-1-ethyl-3-(prop-2-yn-1-yl)pyrimidine-2,4,6(1*H*,3*H*,5*H*)-trione

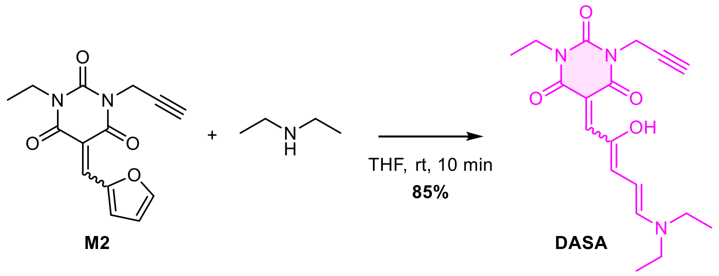

M2 **DASA**

M2 (203 mg, 0.75 mmol) was suspended in 10 mL of THF. Diethylamine (80 µL, 0.77 mmol) was added, followed by a few drops of H_2O. The heterogeneous reaction mixture turned from yellow to dark purple. This suspension was stirred for 10 min at room temperature, until completion of the reaction was confirmed by TLC. Cold pentane was then added to further induce the precipitation of the desired product. The solid was collected by vacuum filtration and thoroughly washed with cold pentane. **DASA** was obtained as a dark purple solid (218 mg, 85% yield).

^{1}H NMR (600 MHz, CD_2Cl_2): δ 12.52 – 12.19 (m, 1H), 7.33 (d, *J* = 12.1 Hz, 1H), 7.06 – 7.00 (m, 1H), 6.85 (d, *J* = 12.5 Hz, 1H), 6.19 – 6.08 (m, 1H), 4.70 – 4.62 (m, 2H), 4.02 – 3.90 (m, 2H), 3.60 – 3.45 (m, 4H), 2.23 – 2.13 (m, 1H), 1.35 – 1.30 (m, 6H), 1.21 – 1.15 (m, 3H).

^{13}C NMR (201 MHz, $CDCl_3$): δ 165.0, 164.3, 162.8, 162.3, 158.4, 158.3, 152.7, 152.6, 151.0, 146.7, 137.2, 103.8, 103.7, 97.5, 97.4, 80.1, 79.9, 70.2, 70.0, 52.6, 44.8, 37.1, 30.9, 30.8, 14.6, 13.6, 13.6, 12.6.

HRMS (m/z, ESI): $[M]^+$ calculated for $C_{18}H_{23}N_3O_4$: 345.16886, found: 345.16785; Δ = -2.93 ppm.

M3 – 2-azido-1-(7-(diethylamino)-9,9-dimethyl-9*H*-fluoren-2-yl)ethan-1-one

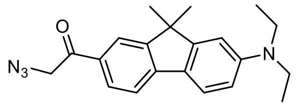

M3

M3 was prepared as detailed in two synthetic steps starting from 2-bromo-9,9-dimethyl-*9H*-fluorene, as described in reference 2. In brief, 2-bromo-9,9-dimethyl-*9H*-fluorene was first reacted with diethylamine via Buchwald-Hartwig amination. The resulting amine was then reacted with bromoacetyl bromide under Friedel-Crafts conditions using triflic acid as solvent. After removing triflic acid and work-up of the reaction, the crude containing the bromo-intermediate was redissolved in DMSO and reacted with sodium azide to yield **M3**, which was obtained as yellow solid after purification by column chromatography. All characterization data were fully consistent with the literature.[2]

First step:

BINAP (0.75 mmol), palladium(II) acetate (0.16 mmol), sodium tert-butoxide (8.13 mmol) and 2-bromo-9,9-dimethylfluorene (7.79 mmol) were added in a round bottom flask and placed under nitrogen. Toluene (15 mL) and diethylamine (9.65 mmol) were added and nitrogen was bubbled in the solution for 5 minutes. The mixture was heated at 110°C overnight. Then, the reaction was cooled to room temperature. The reaction mixture was diluted with DCM, filtered through a plug of celite, and rinsed three times with DCM. Solvents were removed under vacuum. Crude product was purified by flash column chromatography (pentane to pentane/EtOAc, 9:1) to give the *N,N*-diethyl-

9,9-dimethyl-*9H*-fluoren-2-amine as an off-white powder. Alternatively, this product can be obtained with satisfactory purity by trituration with methanol.

^{1}H NMR (600 MHz, DMSO-d_6) δ 7.61 – 7.49 (m, 2H), 7.41 (dt, *J* = 7.4, 0.9 Hz, 1H), 7.22 (td, *J* = 7.4, 1.1 Hz, 1H), 7.12 (td, *J* = 7.4, 1.1 Hz, 1H), 6.78 (d, *J* = 2.4 Hz, 1H), 6.62 (dd, *J* = 8.4, 2.4 Hz, 1H), 3.39 (q, *J* = 7.0 Hz, 4H), 1.39 (s, 6H), 1.12 (t, *J* = 7.0 Hz, 6H). *(From reference 2).*

Second step:

Triflic acid (15 mL) was added to *N,N*-diethyl-9,9-dimethyl-*9H*-fluoren-2-amine (1.82 mmol) under nitrogen atmosphere, and the resulting solution was cooled to 0°C. Bromoacetyl bromide (2.19 mmol) was added dropwise, and the solution was allowed to warm to room temperature while stirring for 1 hour. Then, ice-cold water (100 mL) and 100 mL of EtOAc were added. The biphasic mixture was strongly stirred, and a saturated solution of sodium bicarbonate was added until reaching a pH between 5 and 7. The phases were separated, and the aqueous layer was extracted EtOAc (2 x 75mL). The combined organic layers were washed with brine, dried over $MgSO_4$, filtered and solvents were removed under vacuum. Then, the resultant crude was dissolved in DMSO (5 mL). Sodium azide (3.49 mmol) was added, and the solution was stirred 10 minutes at room temperature. The reaction mixture was poured in water (100 mL) and extracted with EtOAc (3 x 100 mL). The organic layer was dried over $MgSO_4$, filtered, and solvents were removed under vacuum. Crude product was purified by flash column chromatography (pentane to pentane/EtOAc, 4:1) to give compound **M3** as yellow powder.

^{1}H NMR (600 MHz, DMSO-d_6): δ 7.99 (d, *J* = 1.6 Hz, 1H), 7.86 (dd, *J* = 8.0, 1.6 Hz, 1H), 7.70 (d, *J* = 8.0 Hz, 1H), 7.67 (d, *J* = 8.6 Hz, 1H), 6.82 (d, *J* = 2.4 Hz, 1H), 6.67 (dd, *J* = 8.6, 2.4 Hz, 1H), 4.88 (s, 2H), 3.43 (q, *J* = 7.0 Hz, 4H), 1.43 (s, 6H), 1.14 (t, *J* = 7.0 Hz, 6H). (*From reference 2*).

DASA/FRT – 5-((2*Z*,4*E*)-5-(diethylamino)-2-hydroxypenta-2,4-dien-1-ylidene)-1-((1-(2-(7-(diethylamino)-9,9-dimethyl-9*H*-fluoren-2-yl)-2-oxoethyl)-1H-1,2,3-triazol-4-yl)methyl)-3-ethylpyrimidine-2,4,6(1*H*,3*H*,5*H*)-trione

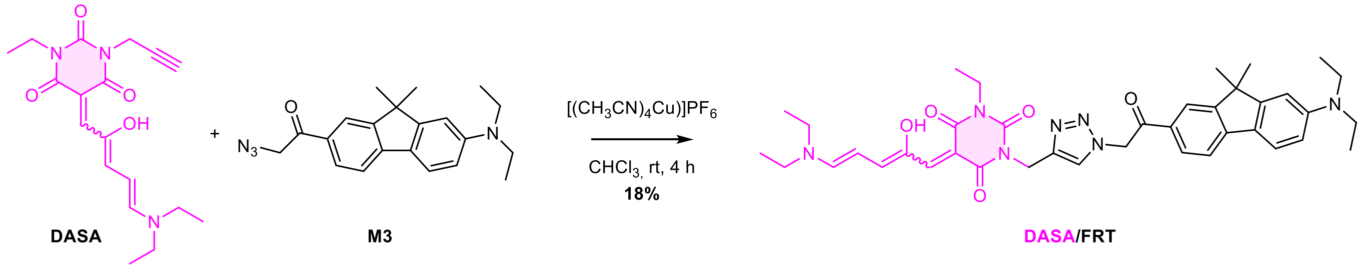

DASA (16.2 mg, 0.047 mmol) and $[Cu(CH_3CN)_4]PF_6$ (35.3 mg, 0.094 mmol) were placed in a round bottom flask under nitrogen atmosphere. Then, **M3** (16.3 mg, 0.047 mmol) was dissolved in 2.0 mL of $CHCl_3$, purged with nitrogen, and added to the prior round bottom flask. The reaction mixture was stirred for 4 h, until completion of the reaction was confirmed by TLC. The solvent was removed, and the resultant dark purple solid was washed with pentane and cold Et_2O. The crude product was purified by reverse phase column chromatography (Sfär C18 D Duo 100 Å 30 μm, H_2O/ACN 95:5 to H_2O) to give **DASA/FRT** in two different fractions, corresponding to the open and closed isomers. These fractions were combined, the solvents removed under vacuum and the resultant solid was redissolved in DCM to promote thermal back isomerization towards the colored isomer. After solvent evaporation, **DASA/FRT** was obtained as a dark purple solid (5.8 mg, 18% yield).

^{1}H NMR (900 MHz, CD_2Cl_2): δ 12.54 – 12.38 (m, 1H), 7.99 – 7.95 (m, 1H), 7.94 – 7.89 (m, 1H), 7.77 – 7.69 (m, 1H), 7.63 (dd, *J* = 7.7, 1.7 Hz, 1H), 7.62 (d, *J* = 8.7 Hz, 1H), 7.30 (d, *J* = 12.3 Hz, 1H), 7.11 – 7.04 (m, 1H), 6.86 – 6.80 (m, 1H), 6.71 (d, *J* = 2.4 Hz, 1H), 6.69 (dd, *J* = 8.4, 2.4 Hz, 1H), 6.17 – 6.06 (m, 1H), 5.85 – 5.81 (m, 2H), 5.26 – 5.22 (m, 2H), 4.02 – 3.90 (m, 2H), 3.56 – 3.41 (m, 8H), 1.48 (s, 6H), 1.33 – 1.30 (m, 6H), 1.23 – 1.19 (m, 9H).

^{13}C NMR was not obtained due to the low solubility of **DASA/FRT** causing poor resolution.

HRMS (m/z, ESI): $[M]^+$ calculated for $C_{39}H_{47}N_7O_5$: 693.36387, found 693.36218; Δ = -2.44 ppm.

- **Synthesis of DASA/Napht and characterization data**

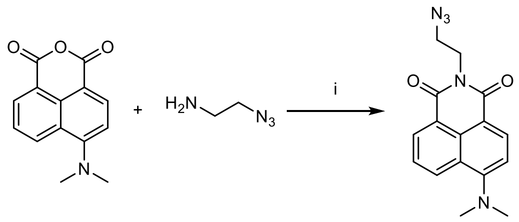

M4 **M5** **M6**

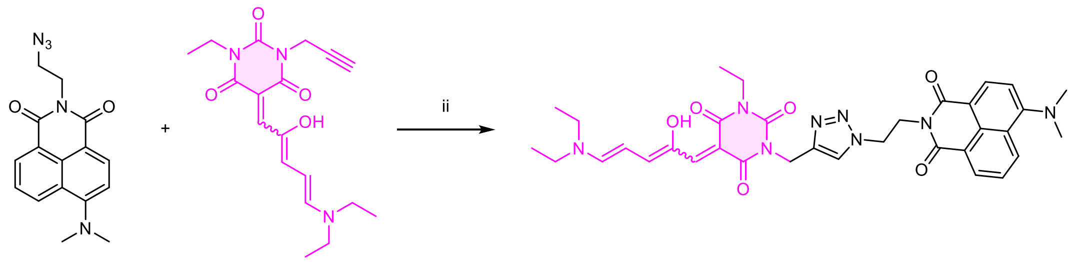

M6 **DASA** **DASA/Napht**

Figure S2. Synthesis of **DASA/Napht**. *Reagents and conditions*: (i) EtOH, reflux, 2 h, **73%**; (ii) $[(CH_3CN)_4Cu)]PF_6$, $CHCl_3$, rt, 4 h, **15%**.

M4 – 6-(dimethylamino)-1*H*,3*H*-benzo[*de*]isochromene-1,3-dione

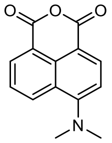

M4

M4 was prepared following a procedure reported in reference 3, without any changes. More concretely, 4-bromo-naphthalic anhydride (14.46 mmol), dimethylamine (48 mmol) and $CuSO_4{\cdot}5H_2O$ (3.28 mmol) were added to DMF (24 mL) in a round bottom flask and the reaction mixture was refluxed at 140 °C for 3 hours. After quenching with water, the precipitate was collected and dried. The solid was dissolved in chloroform and filtered to remove insoluble impurities. After removal of the solvents, the crude product was purified by silica gel column chromatography ($CHCl_3$/hexane = 1/1), to give the desired product as a yellow solid. All characterization data were fully consistent with the literature.[3]

^{1}H NMR (400 MHz, $CDCl_3$): δ 8.59 (dd, *J* = 7.3 Hz, *J* = 1.1 Hz, 1H), 8.49 (d, *J* = 8.2 Hz, 1H), 8.46 (dd, *J* = 8.5 Hz, *J* = 1.1 Hz, 1H), 7.67 (dd, *J* = 8.4 Hz, *J* = 7.4 Hz, 1H), 7.13 (d, *J* = 8.2 Hz, 1H), 4.60 (t, *J* = 7.2 Hz, 2H), 3.66 (t, *J* = 7.2 Hz, 2H), 3.12 (s, 6H). (*From reference 3*).

M5 – 2-azidoethan-1-amine

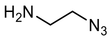

M5

M5 was prepared following a procedure reported in reference 4, without any changes. More concretely, 2-bromoethylamine hydrobromide (30 mmol) was added to a solution of sodium azide (95 mmol) in 26 ml of water and heated to 87°C for 21 h. The reaction mixture was cooled to 0°C, KOH (142 mmol) was added in three portions under stirring at 0°C, and extracted with Et_2O (4 x 35 mL). The combined organic phases were dried over $MgSO_4$, filtered, and evaporated at 34°C (760 mbar) to yield 7.26 g of a colorless etheric solution containing 2-azido-ethylamine dissolved in Et_2O (the proportion was determined by NMR). Caution! The product is very volatile and must not be isolated from the solution; explosive! The yield of the product can be determined by integration of the ^{1}H-NMR spectrum signals. All characterization data were fully consistent with the literature.[4]

^{1}H-NMR (300 MHz, CD_2Cl_2): δ 3.36 (t, 2H), 2.86–2.90 (m, 2H), 1.44 (s, 2H) (*From reference 4*).

M6 – 2-(2-azidoethyl)-6-(dimethylamino)-1*H*-benzo[*de*]isoquinoline-1,3(2*H*)-dione

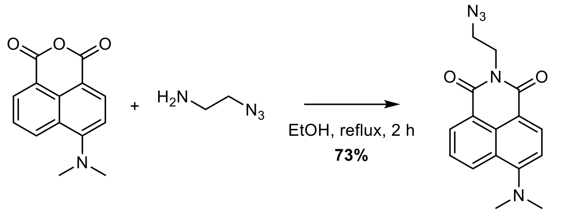

M4 **M5** **M6**

M4 (300 mg, 1.24 mmol) was placed in a round bottom flask under nitrogen atmosphere and absolute EtOH (12 mL) was added. After heating to reflux, **M5** (148 mg, 1.49 mmol) was added. The reaction mixture was refluxed for 2 h, until the completion of the reaction was confirmed by TLC. The solution was then allowed to cool to room temperature, and the resulting solid was collected by vacuum filtration and washed with cold methanol. **M6** was obtained as a yellow solid (280 mg, 73 % yield).

^{1}H NMR (400 MHz, $CDCl_3$): δ 8.59 (dd, *J* = 7.3, 1.2 Hz, 1H), 8.49 (d, *J* = 8.2 Hz, 1H), 8.45 (dd, *J* = 8.5, 1.2 Hz, 1H), 7.67 (dd, *J* = 8.5, 7.3 Hz, 1H), 7.12 (d, *J* = 8.2 Hz, 1H), 4.43 (t, *J* = 6.3 Hz, 2H), 3.65 (t, *J* = 6.3 Hz, 2H), 3.12 (s, 7H).

^{13}C NMR (201 MHz, $CDCl_3$): δ 164.7, 164.1, 156.9, 133.1, 131.6, 131.5, 130.5, 125.4, 125.2, 125.2, 122.9, 114.9, 113.6, 49.1, 45.0, 38.8.

HRMS (m/z, ESI): $[M]^+$ calculated for $C_{16}H_{15}N_5O_2$: 309.12257, found 309.12202; Δ = -1.78 ppm.

DASA/Napht – 5-((2*Z*,4*E*)-5-(diethylamino)-2-hydroxypenta-2,4-dien-1-ylidene)-1-((1-(2-(6-(dimethylamino)-1,3-dioxo-1*H*-benzo[*de*]isoquinolin-2(3*H*)-yl)ethyl)-1H-1,2,3-triazol-4-yl)methyl)-3-ethylpyrimidine-2,4,6(1*H*,3*H*,5*H*)-trione

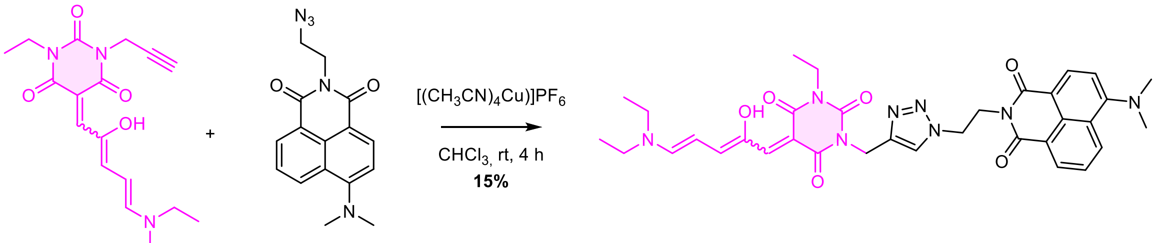

DASA **M6** **DASA/Napht**

DASA (17.0 mg, 0.049 mmol) and $[Cu(CH_3CN)_4]PF_6$ (36.5 mg, 0.098 mmol) were placed in a round bottom flask under nitrogen atmosphere. Then, **M6** (15.0 mg, 0.049 mmol) was dissolved in 2.0 mL of $CHCl_3$, purged with nitrogen, and added to the prior round bottom flask. The reaction mixture was stirred for 4 h, until completion of the reaction was confirmed by TLC. The solvent was then removed, and the resultant dark purple solid was washed with pentane and cold diethyl ether (Et_2O). The crude product was purified by reverse phase column chromatography (Sfär C18 D Duo 100 Å 30 µm, H_2O/ACN 95:5 to H_2O) to give **DASA/Napht** in two different fractions, corresponding to the open and closed isomers. These fractions were combined, the solvents removed under vacuum and the resultant solid was redissolved in DCM to promote thermal back isomerization towards the colored isomer. After solvent evaporation, **DASA/Napht** was obtained as a dark purple solid (4.8 mg, 15% yield).

^{1}H NMR (900 MHz, CD_2Cl_2): δ 12.59 – 12.40 (m, 1H), 8.49 – 8.46 (m, 1H), 8.45 (dt, *J* = 8.5, 1.2 Hz, 1H), 8.38 – 8.34 (m, 1H), 7.69 – 7.61 (m, 2H), 7.32 (d, *J* = 12.2 Hz, 1H), 7.09 (d, *J* = 8.1 Hz, 1H), 7.02 (d, *J* = 8.6 Hz, 1H), 6.85 – 6.80 (m, 1H), 6.15 – 6.07 (m, 1H), 5.14 – 5.07 (m, 2H), 4.67 – 4.61 (m, 2H), 4.59 – 4.54 (m, 2H), 3.91 – 3.85 (m, 2H), 3.56 – 3.51 (m, 2H), 3.51 – 3.46 (m, 2H), 3.11 – 3.09 (m, 6H), 1.34 – 1.31 (m, 6H), 1.14 – 1.10 (m, 3H).

^{13}C NMR was not obtained due to the low solubility of **DASA/Napht** causing poor resolution.

HRMS (m/z, ESI): $[M]^+$ calculated for $C_{34}H_{38}N_8O_6$: 654.29143, found 654.28997; Δ = -2.23 ppm.

- ## Synthesis of NOMe/Napht and characterization data

NOMe/Napht – 2,2'-((((2-(8,9-bis(4-methoxyphenyl)-2'*H*-spiro[benzo[*e*]imidazo[2,1-*a*]isoindole-11,1'-naphthalen]-2'-ylidene)-2*H*-imidazole-4,5-diyl)bis(4,1-phenylene))bis(oxy))bis(ethane-2,1-diyl))bis(6-(dimethylamino)-1*H*-benzo[*de*]isoquinoline-1,3(2*H*)-dione)

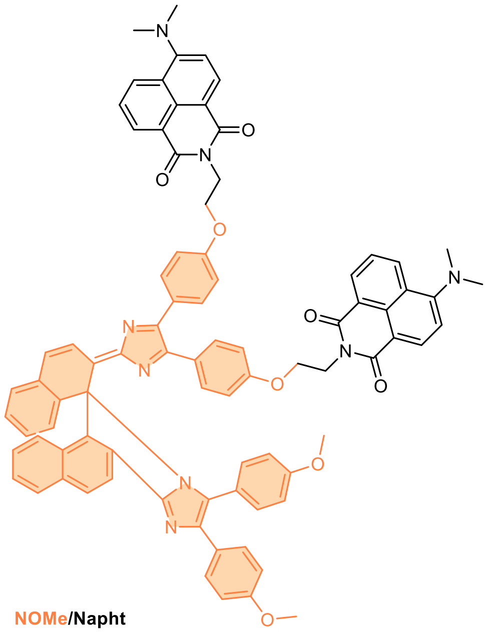

NOMe/Napht

NOMe/Napht was prepared according to a multi-step synthesis described in reference 3. In brief, **NOMe/Napht** was obtained by oxidation of the bis(imidazole) precursor. This bis(imidazole) intermediate was accessed through a sequence of Debus-Radziszewski imidazole synthesis reactions between suitably prepared diketones and [1,1'-binaphthalene]-2,2'-dicarboxaldehyde (**M9**). **M9** was prepared from dibromo-1,1'-binaphthyl by Bouveault formylation (see below). To build the bottom part of the molecule, [1,1'-binaphthalene]-2,2'-dicarboxaldehyde was reacted with one equivalent of *p*-anisil, thus desymmetrizing the scaffold. The diketone required for the top part of the molecule was prepared from 4,4'-dihydroxy-benzil (**M16**, see below), which was readily obtained by demethylation of *p*-anisil under acidic conditions. The conveniently modified naphtalimide moiety was incorporated into the 4,4'-dihydroxy-benzil via Williamson ether synthesis. All characterization data were fully consistent with the literature.[3]

Preparation of the bis(aldehyde): see below preparation of **M9**.

Desymmetrization of the bis(aldehyde) to build the bottom part of **NOMe/Napht***:*

M9 (0.976 mmol), *p*-anisil (0.890 mmol) and ammonium acetate (21.5 mmol) were dissolved in acetic acid (18 mL). The mixture was stirred at 110 °C for 19 hours. After cooling to room temperature, the reaction mixture was neutralized with aqueous NH_3, and the reaction mixture was extracted with EtOAc. The combined organic layer was washed with water and brine, and passed through a phase separator paper. After removal of the solvents, the crude product was purified by silica gel column chromatography (EtOAc /Hexane = 1/2) to give 2'-(4,5-bis(4-methoxyphenyl)-*1H*-imidazol-2-yl)-[1,1'-binaphthalene]-2-carbaldehyde as a pale brown crystal (25 %).

^{1}H NMR (400 MHz, DMSO-d_6): δ 12.51 (s, 1H), 9.72 (s, 1H), 8.28 (s, 1H), 8.20 (d, *J* = 8.7 Hz, 1H), 8.12–8.09 (m, 3H), 7.61–7.53 (m, 2H), 7.37– 7.27 (m, 4H), 7.09 (d, *J* = 8.5 Hz, 1H), 7.0–6.94 (m, 3H), 6.85 (d, *J* = 8.8 Hz, 2H) , 6.65 (d, *J* = 8.8 Hz, 2H), 3.77 (s, 3H), 3.68 (s, 3H). *(From reference 3)*.

Preparation of the diketone required for building the top part of **NOMe/Napht** *(I)*: the first step was the demethylation of *p*-anisil; see below preparation of **M16**.

Adaption of the napthalimide fluorophore (I): the next steps in the synthetic routine refer to the installation of the naphtalimide onto the previously prepared diketone **M16**. The first step of this sequence was the bromo-dimethylamino exchange in 4-bromo-naphtalic anhydride; see above preparation of **M4**.

Adaption of the napthalimide fluorophore (II):

M4 (4.163 mmol) was placed in a round bottom flask under N_2 atmosphere. Dry ethanol (27 mL) was added to the flask and a clear orange solution formed. After heating to 80 °C, ethanolamine (5.3 mmol) was added. The mixture

was refluxed for another 2 hours and the solution was allowed to cool to room temperature. After removal of the solvents, 6-(dimethylamino)-2-(2-hydroxyethyl)-*1H*-benzo[de]isoquinoline-1,3(*2H*)-dione was obtained as an orange solid and used without further purification.

^{1}H NMR (400 MHz, $CDCl_3$): δ 8.59 (dd, J = 7.3 Hz, *J* = 1.1 Hz, 1H), 8.49 (d, *J* = 8.2 Hz, 1H), 8.46 (dd, *J* = 8.5 Hz, *J* = 1.2 Hz, 1H), 7.67 (dd, *J* = 7.9 Hz, *J* = 7.3 Hz, 1H), 7.13 (d, *J* = 8.2 Hz, 1H), 4.60 (t, *J* = 7.2 Hz, 2H), 3.67 (t, *J* = 7.2 Hz, 2H), 3.12 (s, 6H). (*From reference 3*).

Adaption of the napthalimide fluorophore (III):

To a solution of 6-(dimethylamino)-2-(2-hydroxyethyl)-*1H*-benzo[de]isoquinoline-1,3(*2H*)-dione (4.059 mmol) in $CHCl_3$ (23 mL) at 0 °C was added dropwise phosphorus tribromide (15 mmol). The reaction mixture was slowly warmed to room temperature, and stirred for 3 minutes. After removal of the solvent in vacuo, ice and K_2CO_3 aq. (2 M, 10 mL) were added to the crude product. The mixture was extracted with DCM. The organic extract was washed with water and brine, and passed through a phase separator paper. After removal of the solvents, the crude product was purified by silica gel column chromatography (EtOAc/hexane = 1/2), to give 2-(2-bromoethyl)-6-(dimethylamino)-*1H*-benzo[*de*]isoquinoline-1,3(*2H*)-dione as a yellow solid.

^{1}H NMR (400 MHz, $CDCl_3$): δ 8.59 (dd, *J* = 7.3 Hz, *J* = 1.1 Hz, 1H), 8.49 (d, *J* = 8.2 Hz, 1H), 8.46 (dd, *J* = 8.5 Hz, J = 1.1 Hz, 1H), 7.67 (dd, *J* = 8.4 Hz, *J* = 7.4 Hz, 1H), 7.13 (d, *J* = 8.2 Hz, 1H), 4.60 (t, *J* = 7.2 Hz, 2H), 3.66 (t, *J* = 7.2 Hz, 2H), 3.12 (s, 6H). (*From reference 3*).

Preparation of the diketone required for building the top part of **NOMe/Napht** *(II)*: once the napthalimide equipped with a bromo leaving group was obtained, the next step was its reaction with **M16** via Williamson synthesis.

2-(2-bromoethyl)-6-(dimethylamino)-*1H*-benzo[*de*]isoquinoline-1,3(*2H*)-dione (1.24 mmol), 4,4'-dihydroxy-benzil (0.464 mmol), K_2CO_3 (2.59 mmol) and KI (0.26 mmol) were add to a round bottom flask under N_2 atmosphere. The mixture was dissolved in dry CH_3CN (30 mL) and stirred at 80 °C for 25 hours. After cooling to room temperature, the yellow precipitate was collected and dried. The precipitate was dissolved in $CHCl_3$ and filtered to remove insoluble impurities. The organic layer was washed with water and brine, and passed through a phase separator paper. The solvent was evaporated to give 2'-(4,5-bis(4-methoxyphenyl)-*1H*-imidazol-2-yl)-[1,1'- binaphthalene]-2-carbaldehyde as a yellow solid.

^{1}H NMR (400 MHz, $CDCl_3$): δ 8.58 (dd, *J* = 7.3 Hz, *J* = 1.0 Hz, 2H), 8.49 (d, *J* = 8.2 Hz, 2H), 8.45 (dd, *J* = 8.5 Hz, *J* = 1.0 Hz, 2H), 7.87 (m, 4H), 7.66 (dd, *J* = 8.4 Hz, *J* = 7.4 Hz, 2H), 7.12 (d, *J* = 8.2 Hz, 2H), 7.00 (m, 4H), 4.63 (t, *J* = 6.3 Hz, 4H), 4.38 (t, *J* = 6.4 Hz, 4H), 3.12 (s, 12H). *(From reference 3)*.

Preparation of bis(imidazole) precursor – second Debus- Radziszewski reaction:

2'-(4,5-bis(4-methoxyphenyl)-*1H*-imidazol-2-yl)-[1,1'-binaphthalene]-2-carbaldehyde (0.076 mmol), 2,2'-(((oxalylbis(4,1- phenylene))bis(oxy))bis(ethane-2,1-diyl))bis(6-(dimethylamino)-*1H*-benzo[*de*]isoquinoline-1,3(*2H*)-dione) (0.093 mmol) and ammonium acetate (3.69 mmol) were dissolved in $CHCl_3$ (5 mL). The mixture was stirred at 110 °C for 11 hours. After cooling to room temperature, the reaction mixture was added water and extracted with $CHCl_3$. The organic extract was washed with water and brine, and passed through a phase separator paper. The product was purified by silica gel column chromatography (EtOAc /DCM = 2/3) to give the bis(imidazole) precursor as a yellow solid.

^{1}H NMR (400 MHz, DMSO-*d_6*): δ 13.81 (s, 2H), 8.45 (dd, *J* = 14.0 Hz, *J* = 7.9 Hz, 4H), 8.32 (d, *J* = 8.2 Hz, 2H), 8.16–8.14 (m, 2H), 7.99 (dt, *J* = 8.2 Hz, *J* = 3.2 Hz, 4H), 7.74–7.70 (m, 2H), 7.47–7.43 (m, 2H), 7.12 (d, *J* = 8.4 Hz, 2H), 7.13–7.04 (m, 8H), 6.89 (d, *J* = 8.6 Hz, 2H), 6.82 (t, *J* = 8.4 Hz, 4H), 6.71(dd, *J* = 8.7 Hz, *J* = 6.8 Hz, 4H), 4.40–4.35 (m, 4H), 4.19–4.12 (m, 4H), 3.69 (s, 3H), 3.65 (s, 3H) , 3.06 (s, 12H). *(From reference 3)*.

Final step – oxidation of the bis(imidazole) intermediate for the obtention of **NOMe/Napht**:

All manipulations were carried out with the exclusion of light. Under nitrogen, to a solution of 2,2'-((((2-(2'-(4,5-bis(4-methoxyphenyl)-*1H*-imidazol-2-yl)-[1,1'-binaphthalen]-2-yl)-1H-imidazole-4,5-diyl)bis(4,1-phenylene))bis(oxy))bis(ethane-2,1-diyl))bis(6-(dimethylamino)-*1H*-benzo[*de*]isoquinoline-1,3(*2H*)-dione) (0.027 mmol) in toluene (26 mL) was added a solution of potassium ferricyanide (2.41 mmol) and KOH (4.81 mmol) in water (21 mL), and the reaction mixture was vigorously stirred for 4 hours. The organic layer was separated, washed with water, and concentrated. The crude product was purified with silica gel column chromatography (DCM/EtOAc = 5/1 to 2/1) to yield **NOMe/Napht** as a dark red solid.

^{1}H NMR (400 MHz, DMSO-*d_6*): δ 8.50–8.42 (m, 4H), 8.36–8.30 (m, 2H), 8.11 (dd, J = 8.4 Hz, *J* = 1.8 Hz, 1H), 8.03 (t, *J* = 7.6 Hz, 1H), 7.94 (dd, *J* = 8.0 Hz, *J* = 3.2 Hz, 1H), 7.80 (dd, *J* = 9.8 Hz, *J* = 5.9 Hz, 1H), 7.76–7.69 (m, 2H),

7.51 (d, *J* = 7.5 Hz, 1H), 7.45–7.27 (m, 10H), 7.22–7.13 (m, 4H), 6.95–6.89 (m, 4H), 6.83–6.77 (m, 3H), 6.60 (dd, *J* = 8.6 Hz, *J* = 7.3 Hz, 2H) , 6.29 (t, *J* = 8.6 Hz, 2H), 4.43–4.34 (m, 4H), 4.31–4.25 (m, 2H), 4.15–4.10 (m, 2H), 3.77 (d, *J* = 15.1 Hz, S6 6H) , 3.66 (d, *J* = 3.0 Hz, 12H). (*From reference 3*).

Additional characterization data are provided below:

^{1}H NMR (800 MHz, C_6D_6): δ 8.64 – 8.55 (m, 2H), 8.55 – 8.47 (m, 3H), 8.41 – 8.34 (m, 1H), 8.25 – 8.19 (m, 1H), 8.13 – 8.08 (m, 1H), 8.03 (td, *J* = 8.3, 1.2 Hz, 1H), 8.00 (ddd, *J* = 8.3, 3.8, 1.2 Hz, 1H), 7.86 – 7.78 (m, 1H), 7.71 – 7.67 (m, 1H), 7.66 – 7.62 (m, 1H), 7.57 – 7.52 (m, 1H), 7.52 – 7.47 (m, 1H), 7.41 – 7.38 (m, 1H), 7.38 – 7.34 (m, 1H), 7.14 – 7.10 (m, 1H), 7.10 – 7.06 (m, 1H), 7.03 – 6.99 (m, 1H), 6.99 – 6.94 (m, 1H), 6.87 – 6.74 (m, 7H), 6.74 – 6.66 (m, 3H), 6.59 – 6.46 (m, 7H), 4.64 – 4.54 (m, 4H), 4.17 – 4.01 (m, 4H), 3.24 – 3.09 (m, 6H), 2.43 – 2.37 (m, 12H).

HRMS (m/z, ESI): $[M]^+$ calculated for $C_{84}H_{64}N_8O_8$: 1312.4847, found 1312.4910; Δ = 4.80 ppm.

- ## Synthesis of *asy*NOMe/Napht and characterization data

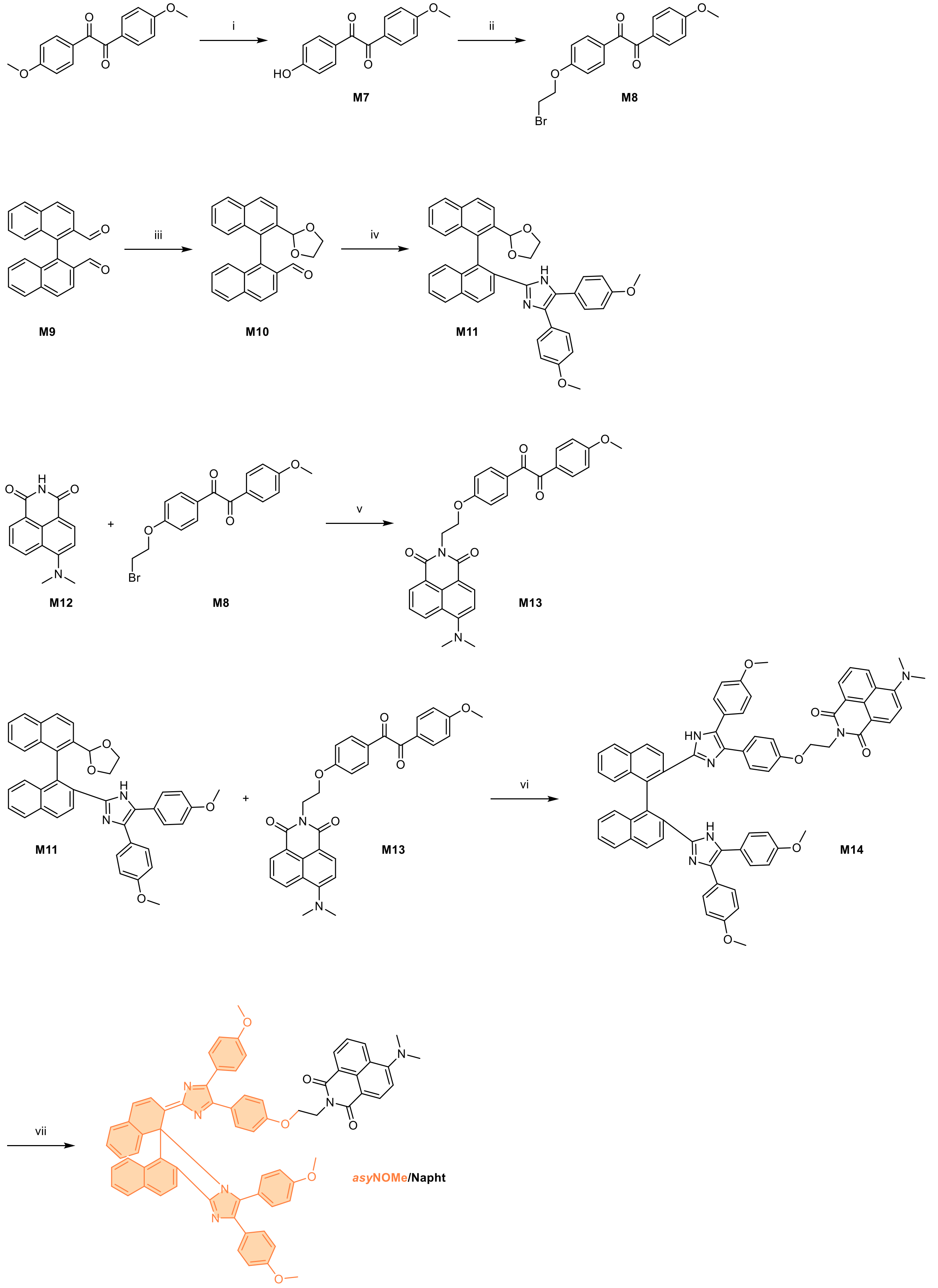

Figure S3. Synthesis of ***asy*NOMe/Napht**. *Reagents and conditions*: (i) HBr_{aq} 48%, AcOH, reflux, 2 h, **43%**; (ii) 1,2-dibromoethane, K_2CO_3, ACN, MW, 100°C, 0.5 h, **quant.**; (iii) ethylene glycol, *p*TSA, toluene, reflux, 12 h, **69%**; (iv) 4,4'-dimethoxybenzil, NH_4OAc, I_2, EtOH, 80°C, 12 h, **91%**; (v) K_2CO_3, DMF, 60°C, overnight, **81%**; (vi) NH_4OAc, AcOH, reflux, overnight, **70%**; (vii) $K_3[Fe(CN)_6]$, KOH, toluene/H_2O, rt, overnight, **46%**.

M7 – 1-(4-hydroxyphenyl)-2-(4-methoxyphenyl)ethane-1,2-dione

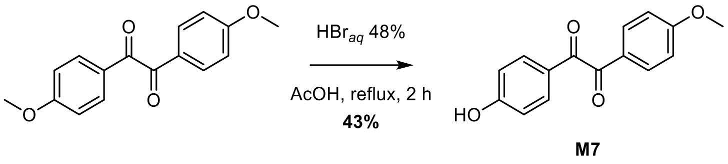

4,4'-Dimethoxybenzil (1.25 g, 4.62 mmol), HBr_{aq} 48% (10 mL, 84.24 mmol) and acetic acid (AcOH; 10 mL) were refluxed for 2 hours. The reaction mixture was then cooled down and quenched by adding H_2O. The solid formed was collected by vacuum filtration and washed with H_2O. The crude product was purified by column chromatography (Sfär Silica D Duo 60 µm, DCM to DCM/EtOAc 9:1) to give **M7** as an off-white solid (0.51 g, 43% yield).

^{1}H NMR (800 MHz, DMSO-d_6): δ 10.85 (broad s, 1H), 7.84 (d, J = 8.9 Hz, 2H), 7.75 (d, J = 8.8 Hz, 2H), 7.13 (d, J = 8.9 Hz, 2H), 6.93 (d, J = 8.8 Hz, 2H), 3.87 (s, 3H).

^{13}C NMR (201 MHz, DMSO-d_6): δ 193.8, 193.4, 164.7, 164.0, 132.3, 132.0, 125.5, 124.1, 116.2, 114.8, 55.8.

HRMS (m/z, ESI): $[M]^+$ calculated for $C_{15}H_{12}O_4$: 256.07356, found 256.07323; Δ = -1.29 ppm.

M8 – 1-(4-(2-bromoethoxy)phenyl)-2-(4-methoxyphenyl)ethane-1,2-dione

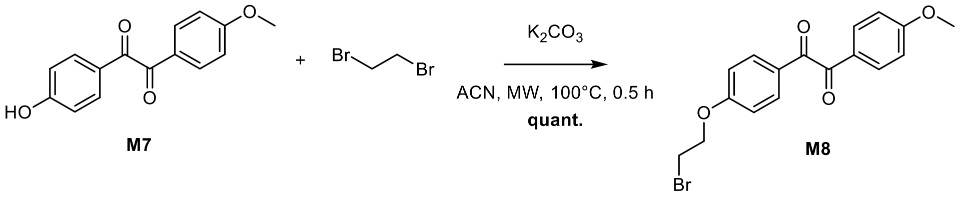

M7 (273 mg, 1.07 mmol), 1,2-dibromoethane (2.0 mL, 23.21 mmol) and K_2CO_3 (736 mg, 5.33 mmol) were placed under nitrogen atmosphere in a sealed vessel. ACN (2.5 mL) were added, and the reaction mixture was heated to 100°C for 0.5 h under microwave irradiation. After confirming completion of the reaction by TLC (DCM/EtOAc 9:1), the reaction mixture was diluted in EtOAc and washed with H_2O and brine. The organic layer was dried over $MgSO_4$, filtered, and the solvent evaporated to give **M8** as a pearl white solid (387 mg, quantitative yield). **M8** was used without further purification.

^{1}H NMR (600 MHz, $CDCl_3$): δ 8.00 – 7.89 (m, 4H), 7.00 – 6.93 (m, 4H), 4.37 (t, J = 6.2 Hz, 2H), 3.89 (s, 3H), 3.66 (t, J = 6.2 Hz, 2H).

^{13}C NMR (201 MHz, $CDCl_3$): δ 193.5, 193.4, 165.0, 163.4, 132.6, 132.5, 127.0, 126.3, 114.9, 114.4, 68.1, 55.8, 28.5.

HRMS (m/z, ESI): $[M]^+$ calculated mass for $C_{17}H_{15}O_4Br$: 362.01537, found 362.01521; Δ = -0.44 ppm.

M9 – [1,1'-binaphthalene]-2,2'-dicarbaldehyde

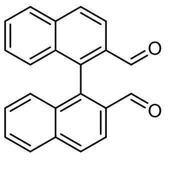

M9

M9 was prepared following a procedure reported in reference 3, without any changes. More concretely, 1.6 M n-BuLi in hexane (19.4 mmol) was added dropwise into a solution of 2,2'- dibromo-1,1'-binaphthyl (5.104 mmol) in 20 mL of dry THF at −78 °C under N_2 atmosphere. The reaction mixture was stirred at −78 °C for 1 hour. Dry DMF (1.4 mL, 15 mmol) was added dropwise to the solution, and the mixture was then stirred at −78 °C for 1 hour. The reaction mixture was slowly warmed to room temperature, and stirred for 15 hours. After quenching with 1N HCl aq., the reaction mixture was extracted with DCM. The organic extract was washed with water and brine, and passed through a phase separator paper. After removal of the solvents, the crude product was purified by silica gel column chromatography

(DCM/hexane = 3/2), to give the desired product as a white powder. All characterization data were fully consistent with the literature.[3]

^{1}H NMR (400 MHz, DMSO-*d6*): δ 9.57 (s, 2H), 8.33 (d, *J* = 8.6 Hz, 2H), 8.20 (d, *J* = 8.1 Hz, 2H), 8.13 (d, *J* = 8.6 Hz, 2H), 7.73 (m, 2H), 7.46 (m, 2H), 7.09 (d, *J* = 8.4 Hz, 2H). (*From reference 3*).

M10 – 2'-(1,3-dioxolan-2-yl)-[1,1'-binaphthalene]-2-carbaldehyde

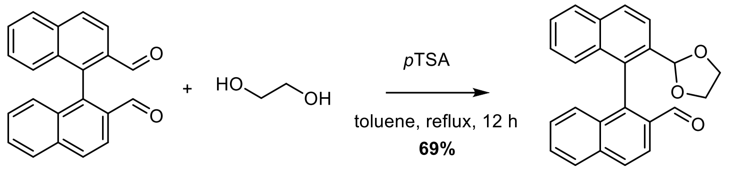

M9 **M10**

M9 (974 mg, 3.14 mmol), ethylene glycol (170 µL, 2.98 mmol) and *p*-toluenesulfonic acid (30 mg, 0.16 mmol) were added to 10 mL of toluene and placed under nitrogen atmosphere. The mixture was heated to reflux overnight. Then, the reaction was cooled to room temperature and water (100 mL) was added. The phases were separated, and the aqueous layer was extracted with EtOAc (3 x 30 mL). The combined organic layers dried over $MgSO_4$, filtered and solvents were removed under vacuum. The crude product was purified by column chromatography (Sfär Silica D Duo 60 µm, pentane to pentane/EtOAc 5:1) to give **M10** as an off-white powder (770 mg, 69% yield).

^{1}H NMR (600 MHz, DMSO-*d6*): δ 9.45 (s, 1H), 8.24 (d, *J* = 8.7 Hz, 1H), 8.22 (d, *J* = 8.7 Hz, 1H), 8.15 (d, *J* = 8.2 Hz, 1H), 8.11 (d, *J* = 7.6 Hz, 1H), 8.06 (d, *J* = 8.6 Hz, 1H), 7.87 (d, *J* = 8.7 Hz, 1H), 7.70 (ddd, *J* = 8.1, 6.7, 1.2 Hz, 1H), 7.58 (ddd, *J* = 8.1, 6.7, 1.2 Hz, 1H), 7.44 (ddd, *J* = 8.3, 6.8, 1.3 Hz, 1H), 7.35 (ddd, *J* = 8.3, 6.7, 1.3 Hz, 1H), 7.10 (d, *J* = 8.5 Hz, 1H), 6.87 (d, *J* = 8.5 Hz, 1H), 5.18 (s, 1H), 4.03 – 3.96 (m, 1H), 3.88 – 3.82 (m, 1H), 3.70 (td, *J* = 7.4, 5.9 Hz, 1H), 3.63 (td, *J* = 7.4, 5.9 Hz, 1H).

^{13}C NMR (151 MHz, DMSO-*d6*): δ 191.2, 141.2, 135.6, 135.1, 133.2, 132.6, 132.2, 132.0, 131.5, 129.4, 129.3, 129.3, 128.5, 128.4, 127.8, 127.4, 127.0, 126.7, 125.8, 123.6, 121.6, 101.0, 65.3, 65.0.

HRMS (m/z, ESI): $[M+H]^+$calculated for $C_{24}H_{19}O_3$: 355.1334, found 355.1331; Δ = -0.84 ppm.

M11 – 2-(2'-(1,3-dioxolan-2-yl)-[1,1'-binaphthalen]-2-yl)-4,5-bis(4-methoxyphenyl)-4,5-dihydro-1*H*-imidazole

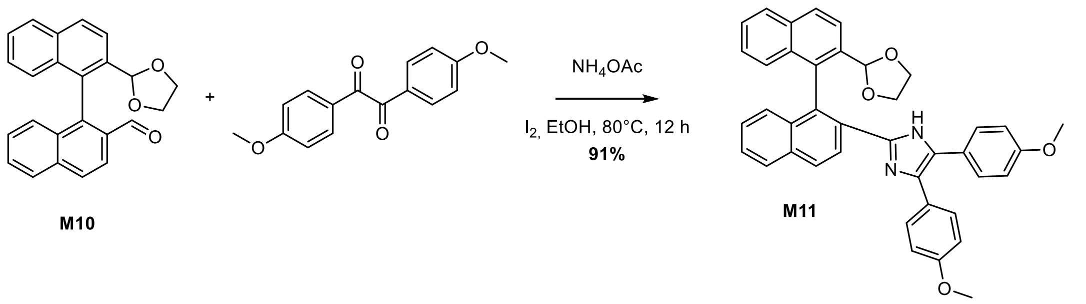

M10 **M11**

M10 (531.0 mg, 1.5 mmol), 4,4'-dimethoxybenzil (446.0 mg, 1.65 mmol), ammonium acetate (2.31 g, 29.97 mmol), iodine (19.0 mg, 0.08 mmol) were added to 5 mL of EtOH and placed under nitrogen. The mixture was heated to 80°C overnight. The reaction was cooled to room temperature and was poured into 50 mL of H_2O and extracted with EtOAc (3 × 30 mL). The combined organic layers dried over $MgSO_4$, filtered and solvents were removed under vacuum. The crude product was purified by column chromatography (Sfär Silica D Duo 60 µm, pentane to pentane/EtOAc 4:1) to give **M11** as pale yellow puffy solid (820 mg, 91% yield).

^{1}H NMR (600 MHz, DMSO-*d6*): δ 11.18 (s, 1H), 8.25 (d, *J* = 8.7 Hz, 1H), 8.19 (d, *J* = 8.8 Hz, 1H), 8.13 (d, *J* = 8.7 Hz, 1H), 8.06 (d, *J* = 8.2 Hz, 1H), 8.02 (d, *J* = 8.2 Hz, 1H), 7.85 (d, *J* = 8.6 Hz, 1H), 7.52 (ddd, *J* = 8.0, 6.7, 1.2 Hz, 1H), 7.47 (ddd, *J* = 8.0, 6.7, 1.2 Hz, 1H), 7.33 (ddd, *J* = 8.2, 6.7, 1.3 Hz, 1H), 7.22 (ddd, *J* = 8.2, 6.7, 1.3 Hz, 1H), 7.09 (d, *J* = 8.8 Hz, 2H), 7.04 (d, *J* = 8.8 Hz, 2H), 7.00 (d, *J* = 9.0 Hz, 1H), 6.94 – 6.91 (m, 2H), 6.90 (d, *J* = 9.0 Hz, 1H), 6.70 – 6.66 (m, 2H), 5.46 (s, 1H), 4.03 – 3.94 (m, 2H), 3.76 (s, 3H), 3.73 – 3.63 (m, 2H), 3.69 (s, 3H)

^{13}C NMR (151 MHz, DMSO-*d$_6$*): 158.7, 157.8, 144.1, 136.2, 135.7, 134.2, 133.6, 133.2, 132.5, 132.3, 131.2, 128.9, 128.5, 128.1, 128.0, 127.6, 127.4, 126.9, 126.4, 126.4, 126.3, 126.2, 125.9, 125.7, 123.5, 123.1, 114.2, 113.3, 101.3, 65.1, 55.2, 55.0

HRMS (m/z, ESI): $[M+H]^+$ calculated for $C_{40}H_{33}N_2O_4$: 605.2440, found 605.2438; Δ = -0.33 ppm.

M12 – 6-(dimethylamino)-1H-benzo[de]isoquinoline-1,3(2*H*)-dione

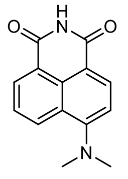

M12

M12 was prepared following a procedure reported in reference 5, without any changes. More concretely, a suspension of **M4** (10 mmol) in ethanol (20 mL) and aqueous ammonia (25 mL) was heated at 70 °C in a sealed tube overnight. After this time, the mixture was cooled, filtered, and the resulting solid washed with hexane to afford pure 1,8-naphthalimide as a yellow solid.

^{1}H NMR (600 MHz, DMSO-*d$_6$*): δ 11.48 (s, 1zH), 8.52 (d, *J* = 8.5 Hz, 1H), 8.42 (d, *J* = 7.2 Hz, 1H), 8.31 (d, *J* = 8.2 Hz, 1H), 7.77 (t, *J* = 8.2 Hz, 1H), 7.23 (d, *J* = 8.2 Hz, 1H), 3.08 (s, 6H).

M13 – 6-(dimethylamino)-2-(2-(4-(2-(4-methoxyphenyl)-2-oxoacetyl)phenoxy)ethyl)-1*H*-benzo[*de*]isoquinoline-1,3(2*H*)-dione

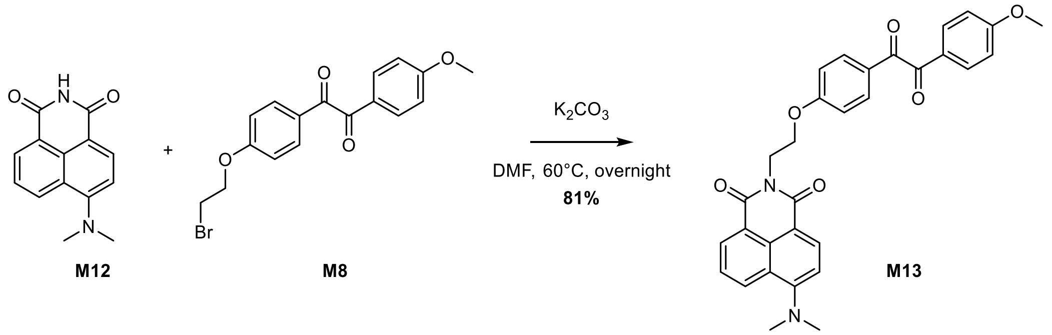

M12 (70 mg, 0.29 mmol), **M8** (70 mg, 0.19 mmol) and K_2CO_3 (80 mg, 0.58 mmol) were placed under nitrogen atmosphere in a sealed vessel. DMF (3 mL) were added, and the reaction mixture was heated to 60°C overnight. After confirming completion of the reaction by TLC (DCM/EtOAc 9:1), the reaction mixture was diluted in DCM and washed several times with water and brine. The organic layer was dried over $MgSO_4$, filtered, and the solvent evaporated under vacuum. The crude product was purified by column chromatography (Sfär Silica D Duo 60 µm, DCM to DCM/EtOAc 9:1) to afford **M13** as a bright yellow solid (82 mg, 81% yield).

^{1}H NMR (600 MHz, DMSO-*d$_6$*): δ 8.53 (dd, *J* = 8.4, 1.2 Hz, 1H), 8.48 (dd, *J* = 7.3, 1.2 Hz, 1H), 8.37 (d, *J* = 8.3 Hz, 1H), 7.86 – 7.83 (m, 2H), 7.83 – 7.80 (m, 2H), 7.76 (dd, *J* = 8.4, 7.3 Hz, 1H), 7.22 (d, *J* = 8.3 Hz, 1H), 7.15 – 7.13 (m, 2H), 7.13 – 7.11 (m, 2H), 4.46 (t, *J* = 6.2 Hz, 2H), 4.38 (t, *J* = 6.3 Hz, 2H), 3.86 (s, 3H), 3.10 (s, 6H).

^{13}C NMR (201 MHz, DMSO-*d$_6$*): δ 193.5, 164.8, 163.8, 163.7, 163.1, 156.7, 132.5, 132.0, 131.8, 130.8, 129.8, 125.6, 125.5, 125.4, 125.0, 124.2, 122.2, 115.4, 115.3, 114.8, 113.0, 113.0, 65.0, 55.8, 44.4, 38.1.

HRMS (m/z, ESI): $[M]^+$ calculated for $C_{31}H_{26}N_2O_6$: 522.17909, found 522.17909; Δ = 0.00 ppm.

M14 – 2-(2-(4-(2-(2'-(4,5-bis(4-methoxyphenyl)-4,5-dihydro-1*H*-imidazol-2-yl)-[1,1'-binaphthalen]-2-yl)-5-(4-methoxyphenyl)-1*H*-imidazol-4-yl)phenoxy)ethyl)-6-(dimethylamino)-1*H*-benzo[*de*]isoquinoline-1,3(2*H*)-dione

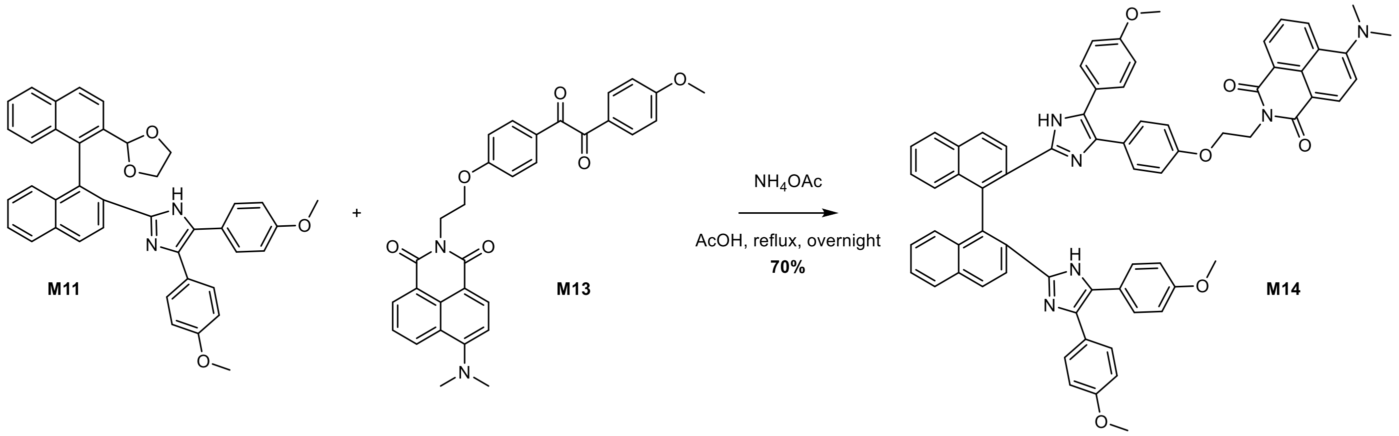

M11 (47mg, 0.08 mmol), **M13** (40 mg, 0.08 mmol) and NH_4OAc (327 mg, 4.24 mmol) were placed under nitrogen atmosphere in a sealed vessel. AcOH (3 mL) were added, and the reaction mixture was heated to reflux overnight. After confirming the completion of the reaction by TLC (DCM/EtOAc 1:1), the reaction mixture was diluted in EtOAc and washed with saturated NH_4OH, H_2O and brine. The organic layer was dried over $MgSO_4$, filtered, and the solvent evaporated under vacuum. The crude product was purified by column chromatography (Sfär Silica D Duo 60 µm, DCM to DCM/EtOAc 1:9) to afford **M14** as a light orange solid (57 mg, 70% yield).

^{1}H NMR (800 MHz, DMSO-*d$_6$*): δ 13.84 – 13.78 (m, 1H), 13.78 – 13.71 (m, 1H), 8.55 – 8.49 (m, 1H), 8.49 – 8.43 (m, 1H), 8.35 (dd, *J* = 8.3, 2.0 Hz, 1H), 8.15 (dd, *J* = 8.8, 2.4 Hz, 2H), 8.01 (dt, *J* = 8.1, 2.0 Hz, 2H), 7.98 (dd, *J* = 8.6, 3.6 Hz, 2H), 7.75 (ddd, *J* = 8.3, 7.2, 3.6 Hz, 1H), 7.48 – 7.41 (m, 2H), 7.28 – 7.22 (m, 2H), 7.21 (dd, *J* = 8.3, 1.6 Hz, 1H), 7.15 – 7.04 (m, 8H), 6.92 – 6.87 (m, 2H), 6.86 – 6.78 (m, 4H), 6.75 – 6.66 (m, 4H), 4.45 – 4.32 (m, 2H), 4.23 – 4.11 (m, 2H), 3.74 – 3.62 (m, 9H), 3.11 – 3.06 (m, 6H).

^{13}C NMR (201 MHz, DMSO-*d$_6$*): δ 163.8, 163.0, 158.6, 157.9, 157.5, 156.9, 156.8, 156.7, 146.4, 135.3, 135.2, 135.1, 134.4, 134.4, 133.0, 132.5, 131.7, 130.7, 130.2, 130.1, 129.7, 128.7, 128.7, 128.6, 128.2, 127.9, 127.4, 127.1, 127.1, 127.0, 126.9, 126.5, 126.4, 125.6, 125.0, 124.2, 123.3, 123.1, 123.0, 122.2, 114.6, 114.1, 113.6, 113.1, 113.0, 113.0, 64.3, 64.2, 55.1, 54.9, 44.4, 38.2, 38.2.

HRMS (m/z, ESI): $[M]^+$ calculated for $C_{69}H_{54}N_6O_6$: 1062.41048, found 1062.41043; Δ = -0.05 ppm.

***asy*NOMe/Napht** – 2-(2-(4-(2-(8,9-bis(4-methoxyphenyl)-2'*H*-spiro[benzo[*e*]imidazo[2,1-*a*]isoindole-11,1'-naphthalen]-2'-ylidene)-5-(4-methoxyphenyl)-2*H*-imidazol-4-yl)phenoxy)ethyl)-6-(dimethylamino)-1*H*-benzo[*de*]isoquinoline-1,3(2*H*)-dione

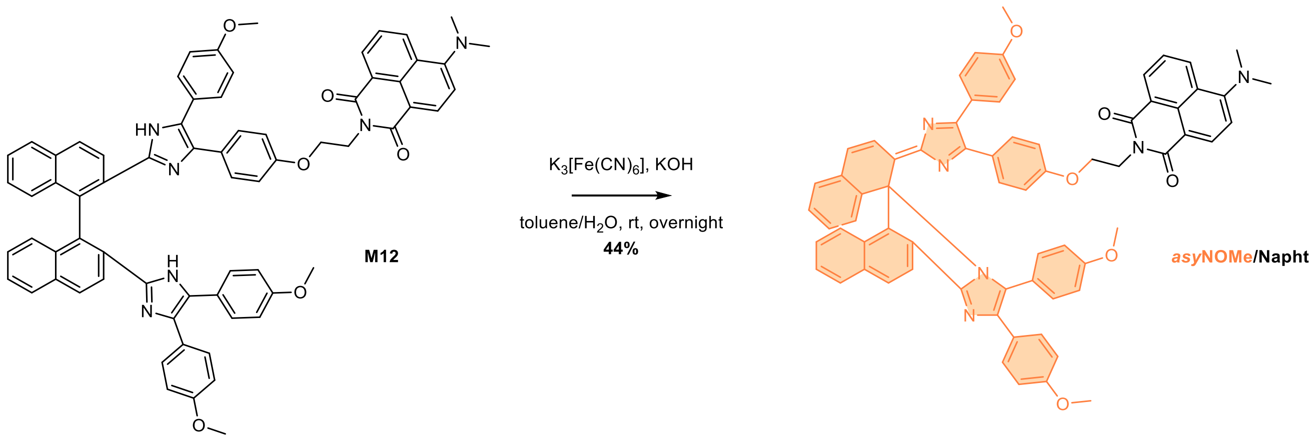

M14 (29 mg, 0.03 mmol) was dissolved in 3 mL of toluene and placed under nitrogen. A solution of KOH (90 mg, 1.64 mmol) and $K_3[Fe(CN)_6]$ (296 mg, 0.82 mmol) in 3 mL water was placed under nitrogen and added to the toluene solution. The reaction mixture was vigorously stirred overnight at room temperature. After confirming the reaction

completion by TLC (DCM/EtOAc 1:1), the reaction mixture was diluted in DCM and washed with water. The organic layer was dried over $MgSO_4$, filtered, and the solvent evaporated under vacuum. The crude product was purified by column chromatography (Sfär Silica HC High Capacity 20 µm, DCM to DCM/EtOAc 8:2) to ***asy*****NOMe/Napht** as a dark red solid (13.4 mg, 46% yield).

^{1}H NMR (600 MHz, DMSO-*d$_6$*): δ 8.57 – 8.45 (m, 2H), 8.41 – 8.33 (m, 1H), 8.14 – 8.09 (m, 1H), 8.07 – 8.01 (m, 1H), 7.97 – 7.91 (m, 1H), 7.84 – 7.72 (m, 2H), 7.53 – 7.48 (m, 1H), 7.45 – 7.30 (m, 8H), 7.25 – 7.17 (m, 3H), 6.98 – 6.88 (m, 4H), 6.84 – 6.74 (m, 3H), 6.63 – 6.57 (m, 2H), 6.32 – 6.27 (m, 2H), 4.49 – 4.11 (m, 3H), 3.81 – 3.64 (m, 9H), 3.13 – 3.05 (m, 6H).

^{13}C NMR (151 MHz, DMSO-*d$_6$*): δ 164.0, 163.9, 163.9, 163.9, 163.8, 163.8, 163.8, 163.7, 163.7, 163.6, 163.1, 163.0, 161.6, 161.6, 161.4, 160.9, 160.3, 159.9, 158.9, 157.9, 157.7, 156.7, 156.7, 156.6, 152.4, 152.4, 152.3, 151.2, 151.2, 140.9, 140.9, 138.0, 137.9, 136.5, 136.5, 133.7, 133.1, 132.5, 131.8, 131.7, 131.4, 131.2, 131.1, 130.9, 130.8, 130.2, 130.0, 129.8, 129.8, 129.5, 129.1, 129.0, 127.8, 127.7, 127.4, 127.1, 127.1, 127.0, 126.5, 125.8, 125.6, 125.5, 125.3, 125.3, 125.2, 125.2, 125.1, 125.0, 124.7, 124.5, 124.4, 124.2, 122.2, 122.2, 121.6, 117.9, 117.8, 114.3, 114.2, 114.1, 113.9, 113.8, 113.7, 113.6, 113.5, 113.1, 113.1, 113.0, 113.0, 65.8, 65.8, 65.8, 64.5, 64.2, 64.2, 55.4, 55.3, 55.1, 55.1, 55.1, 54.9, 44.4, 38.3, 38.2.

HRMS (m/z, ESI): $[M]^+$ calculated for $C_{69}H_{24}N_6O_6$: 1060.39483 found, 1060.39369; Δ = -1.08 ppm.

- **Synthesis of NOMe/Phtha and characterization data**

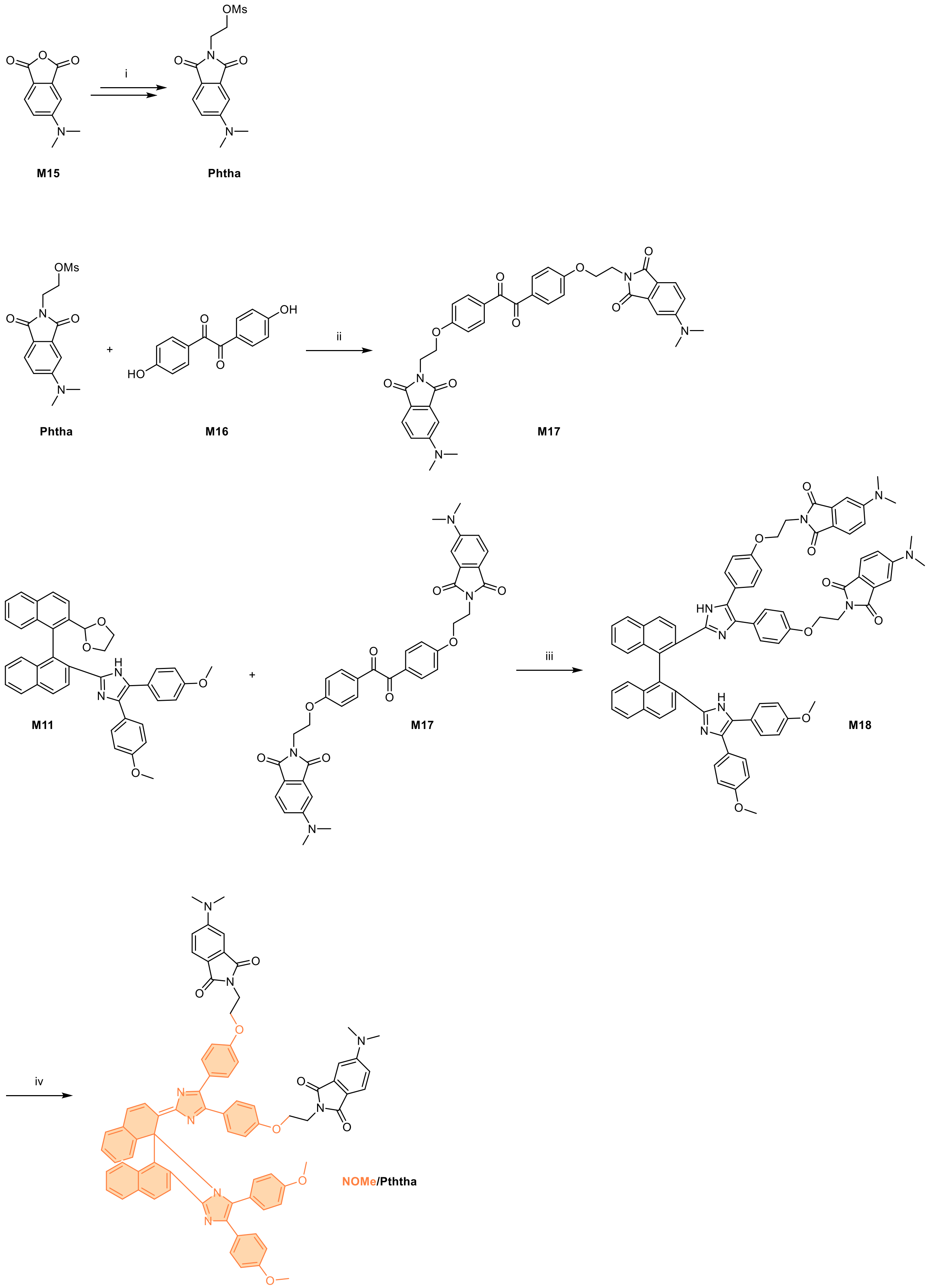

Figure S4. Synthesis of **NOMe/Phtha**. *Reagents and conditions*: (i) 1) ethanolamine, EtOH, reflux, 12 h, 2) MsCl, Et_3N, DCM, 0°c to rt, 12 h, **95%** over two steps; (ii) K_2CO_3, KI, ACN, reflux, 12 h, **43%**; (iii) NH_4OAc, AcOH, $CHCl_3$, 110°C, 12 h, **80%**; (vii) $K_3[Fe(CN)_6]$, KOH, toluene/H_2O, rt, 4 h, **61%**.

M15 – 5-(dimethylamino)isobenzofuran-1,3-dione

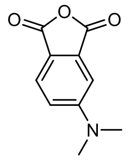

M15

M15 was prepared following a procedure reported in reference 6, without any changes in it. More concretely, the protocol reads as follows:

1. Weigh 500 mg of 4-aminophthalic acid into a 500 ml two-neck round-bottom flask equipped with a Teflon-coated stir bar.
2. Add 150 ml of methanol and turn the magnetic stirrer on.
3. Attach a three-way flushing adapter secured with a keck clip to the flask and connect a vacuum and nitrogen line to one of the inlets. Stopper the flask by using a rubber septum.
4. Purge the solution by alternating three cycles of evacuation/inert gas saturation.
5. Briefly remove the septum and under a gentle stream of inert gas add 15 ml of a 37% solution of formaldehyde followed by 200 mg of Pd/C 10%.
6. Purge the solution by alternating three cycles of evacuation/inert gas saturation.
7. Fill a thick-walled balloon equipped with a three-way stopcock with hydrogen. Adapt a needle to the stopcock and connect the balloon to the reaction vessel via the rubber septum.
8. Purge with hydrogen and stir for 3 h under hydrogen atmosphere.
9. Purge with inert gas.
10. Pack a pad of Celite (~3 cm) into a fritted glass funnel (coarse frit, 8.5 cm internal diameter (i.d.) × 7.3 cm height) and wash two times with 20 ml of methanol.
11. Filter the suspension through the pad of Celite and wash two times with 50 ml of methanol.
12. Transfer the filtrate into a 1 liter round-bottom flask, evaporate the solvent on a rotary evaporator at 25 °C and dry the residual white solid on a vacuum pump (~2 h).

All characterization data were fully consistent with the literature.[6]

^{1}H NMR (400 MHz, $CDCl_3$): δ 3.0 (s, 6H), 6.9 (dd, 1H, J_1 = 2.4 Hz, J_2 = 8.7 Hz), 7.07 (d, 1H, J = 2.4 Hz), 7.75 (d, 1H J = 8.6 Hz). (*From reference 6*).

M16 – 1,2-bis(4-hydroxyphenyl)ethane-1,2-dione

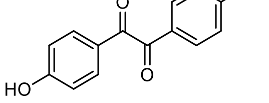

M16

M16 was prepared following a procedure reported in reference 3, without any changes. More concretely, *p*-anisil (3.70 mmol), HBr aq. (8 mL, 47.0–49.0%) and acetic acid (8 mL) were added to a round bottom flask, and the mixture was refluxed for 21 hours. After quenching with water, the reaction mixture was extracted with EtOAc. The organic extract was washed with water and brine, and passed through a phase separator paper. After removal of the solvents, the crude product was purified by silica gel column chromatography (EtOAc/hexane = 1/1), to give the desired product as a pale-yellow solid. All characterization data were fully consistent with the literature.[3]

^{1}H NMR (400 MHz, DMSO-d_6): δ 10.84 (broad s, 2H), 7.75–7.72 (m, 4H), 6.94–6.90 (m, 4H). (*From reference 3*).

Phtha – 2-(5-(dimethylamino)-1,3-dioxoisoindolin-2-yl)ethyl methanesulfonate

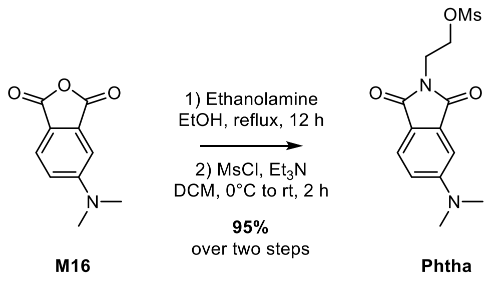

M16 (900 mg, 4.71 mmol), ethanolamine (370 µL, 6.12 mmol) and ethanol (15 mL) were added and placed under nitrogen. Nitrogen was bubbled in the solution for 5 minutes. The mixture was heated to reflux overnight. The reaction was cooled to room temperature. Solvents were removed under vacuum and the crude product was used directly in the next step without further purification. To this crude product, DCM (15 mL) and triethylamine (2 mL) were added, and the solution was cooled down to 0°C. Then, mesyl chloride (730 µL, 9.42 mmol) was added dropwise. The reaction mixture was slowly warmed to room temperature and stirred for 2 hours, and water was added. The phases were separated, and the aqueous layer was extracted with DCM (2 x 30mL). The combined organic layers were dried over $MgSO_4$, filtered and the solvents were removed under vacuum. The crude product was purified by column chromatography (Sfär Silica D Duo 60 µm, pentane/EtOAc 2:1) to give **Phtha** as a yellow powder (1.47 g, 95% yield over two steps).

^{1}H NMR (600 MHz, DMSO-d_6): δ 7.63 (d, J = 8.5 Hz, 1H), 7.06 (d, J = 2.4 Hz, 1H), 6.94 (dd, J = 8.5, 2.4 Hz, 1H), 4.38 (t, J = 5.3 Hz, 2H), 3.85 (t, J = 5.3 Hz, 2H), 3.14 (s, 3H), 3.09 (s, 6H).

^{13}C NMR (151 MHz, DMSO-d_6): δ 168.6, 168.1, 154.8, 134.6, 125.1, 116.7, 115.4, 105.8, 67.5, 37.3, 37.2.

HRMS (m/z, ESI): $[M+H]^+$ calculated for $C_{13}H_{17}N_2O_5S$: 313.0858, found 313.0854; Δ = -1.27 ppm.

M17 – 2,2'-(((oxalylbis(4,1-phenylene))bis(oxy))bis(ethane-2,1-diyl))bis(5-(dimethylamino)isoindoline-1,3-dione)

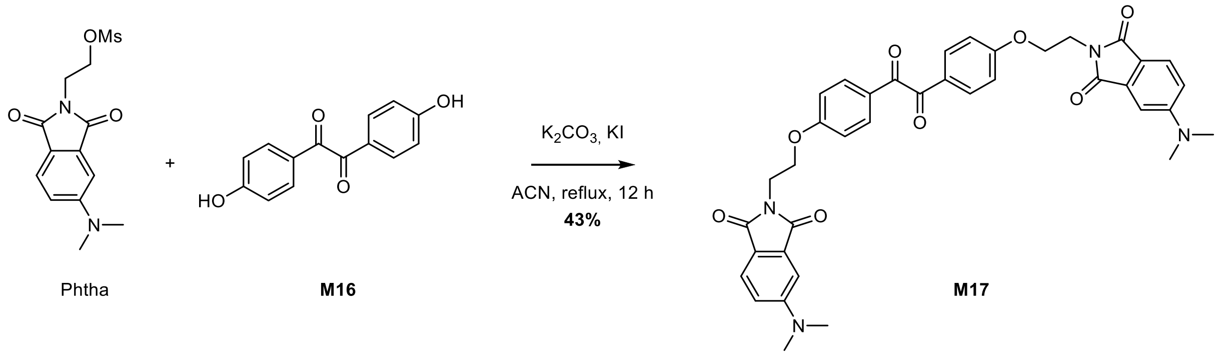

Phtha (567.4 mg, 1.82 mmol), **M16** (220 mg, 0.91 mmol), potassium carbonate (647 mg, 4.68 mmol) and potassium iodide (93 mg, 0.56 mmol) were added to 15 mL acetonitrile and placed under nitrogen. Nitrogen was bubbled in the solution for 5 minutes. The mixture was heated to reflux overnight. Then, the reaction was cooled to room temperature. Solvents were removed under vacuum, and the crude was triturated with cold methanol and filtered to give **M17** as a yellow powder (265 mg, 43% yield).

^{1}H NMR (600 MHz, DMSO-d_6): δ 7.79 (d, J = 8.9 Hz, 4H), 7.61 (d, J = 8.5 Hz, 2H), 7.07 (d, J = 8.9 Hz, 4H), 7.04 (d, J = 2.4 Hz, 2H), 6.92 (dd, J = 8.5, 2.4 Hz, 2H), 4.31 (t, J = 5.6 Hz, 4H), 3.93 (t, J = 5.6 Hz, 4H), 3.08 (s, 12H).

^{13}C NMR (151 MHz, DMSO-d_6): δ 193.5, 168.2, 167.8, 163.6, 154.3, 134.1, 132.0, 125.6, 124.6, 116.2, 115.3, 114.9, 105.3, 65.4, 36.4.

HRMS (m/z, ESI): $[M+H]^+$ calculated for $C_{38}H_{35}N_4O_8$: 675.2455, found 675.2450; Δ = -0.74 ppm.

M18 – 2,2'-((((2-(2'-(4,5-bis(4-methoxyphenyl)-4,5-dihydro-1H-imidazol-2-yl)-[1,1'-binaphthalen]-2-yl)-1*H*-imidazole-4,5-diyl)bis(4,1-phenylene))bis(oxy))bis(ethane-2,1-diyl))bis(5-(dimethylamino)isoindoline-1,3-dione)

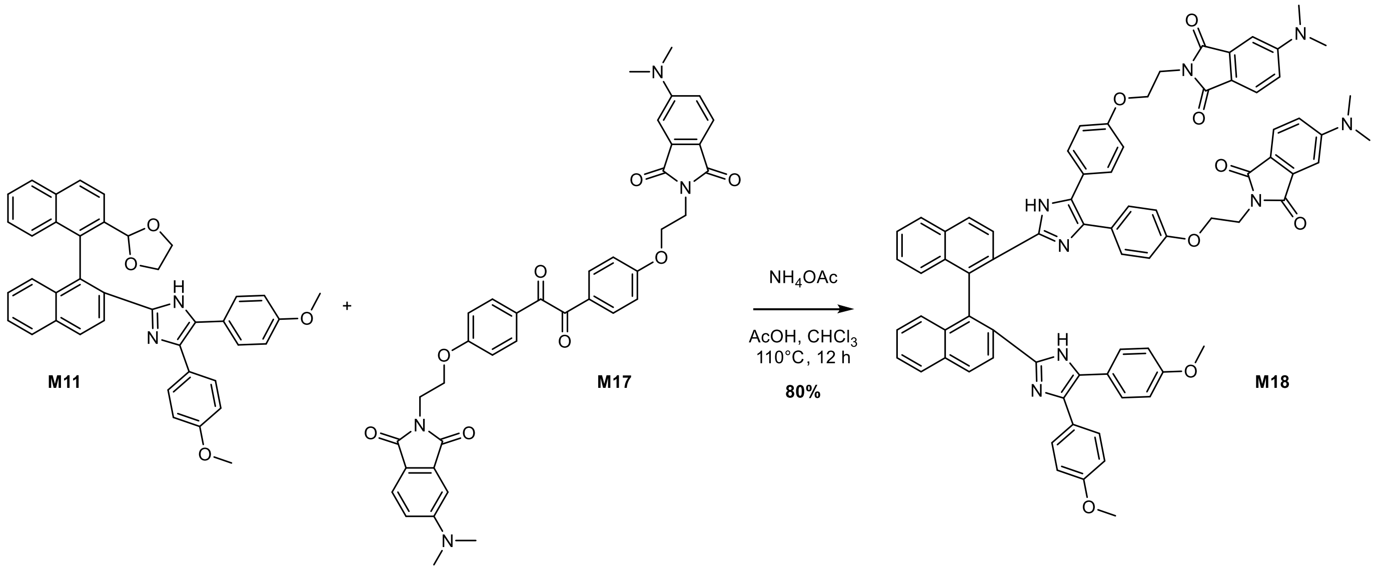

M11 (35.9 mg, 0.059 mmol), **M17** (40 mg, 0.059 mmol) and ammonium acetate (228 mg, 2.96 mmol) were dissolved in chloroform (1 mL) and acetic acid (3 mL). The mixture was stirred at 110 °C in a sealed vessel overnight. After cooling to room temperature, the reaction mixture was added to ammonium hydroxide solution (20 mL). The resulting aqueous solution was extracted with EtOAc (3 x 30mL). The combined organic layers were dried over $MgSO_4$, filtered and solvents were removed under vacuum. Crude product was purified by column chromatography (Sfär Silica D Duo 60 µm, DCM/EtOAc 2:3) to give **M18** as a yellow solid (58 mg, 80% yield).

^{1}H NMR (600 MHz, DMSO-*d$_6$*): δ 13.78 (s, 1H), 13.75 (s, 1H), 8.19 – 8.10 (m, 2H), 8.00 (dd, *J* = 8.2, 4.2 Hz, 2H), 7.97 (dd, *J* = 8.5, 3.5 Hz, 2H), 7.60 (d, *J* = 8.5 Hz, 2H), 7.45 (td, *J* = 7.2, 4.4 Hz, 2H), 7.24 (td, *J* = 7.0, 2.6 Hz, 2H), 7.14 – 6.99 (m, 10H), 6.94 – 6.85 (m, 4H), 6.81 (d, *J* = 8.4 Hz, 2H), 6.76 (d, *J* = 8.4 Hz, 2H), 6.69 (d, *J* = 8.7 Hz, 2H), 6.64 (d, *J* = 8.6 Hz, 2H), 4.12 (t, *J* = 5.7 Hz, 2H), 4.08 (t, *J* = 5.8 Hz, 2H), 3.88 – 3.81 (m, 4H), 3.70 (s, 3H), 3.66 (s, 3H), 3.07 (s, 12H).

^{13}C NMR (151 MHz, DMSO-*d$_6$*): δ 168.2, 167.8, 158.6, 157.9, 156.8, 154.3, 146.5, 135.2, 134.4, 134.1, 133.0, 132.5, 130.1, 128.7, 128.2, 127.9, 127.4, 126.9, 126.5, 125.7, 124.5, 123.4, 123.0, 116.3, 114.9, 114.7, 114.1, 113.6, 105.3, 64.6, 55.1, 54.9, 36.7.

HRMS (m/z, ESI): $[M+H]^+$ calculated for $C_{76}H_{63}N_8O_8$: 1215.4769, found 1215.4828; Δ = 4.85 ppm.

NOMe/Phtha – 2,2'-((((2-(8,9-bis(4-methoxyphenyl)-2'*H*-spiro[benzo[*e*]imidazo[2,1-*a*]isoindole-11,1'-naphthalen]-2'-ylidene)-2*H*-imidazole-4,5-diyl)bis(4,1-phenylene))bis(oxy))bis(ethane-2,1-diyl))bis(5-(dimethylamino)isoindoline-1,3-dione)

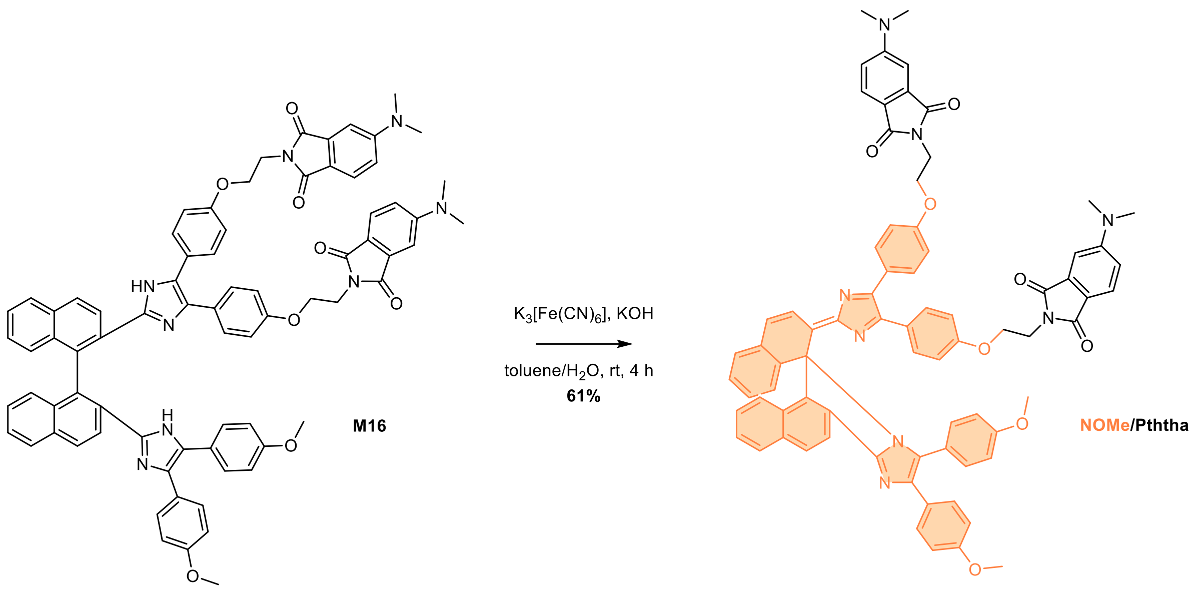

M16 (33.0 mg, 0.027 mmol) was dissolved in toluene (2 mL) and placed under nitrogen. A solution of potassium hydroxide (91 mg, 61 mmol) and potassium ferricyanide (194 mg, 0.59 mmol) in water (3 mL) was prepared under nitrogen and added to the toluene solution. The reaction mixture was vigorously stirred for 4 hours at room temperature. The organic layers were separated and washed with water (3 x 20 mL), dried over $MgSO_4$, and filtered. The solvents were removed under vacuum. The crude product was purified by column chromatography (Sfär Silica D Duo 60 µm, DCM/MeOH 97:3) to give **NOMe/Phtha** as dark red solid (20 mg, 61% yield).

^{1}H NMR (800 MHz, C_6D_6): δ 8.59 – 8.51 (m, 1H), 8.37 (t, *J* = 9.3 Hz, 1H), 8.13 (d, *J* = 8.4 Hz, 1H), 8.01 (d, *J* = 8.4 Hz, 1H), 7.85 – 7.75 (m, 1H), 7.68 (d, *J* = 8.3 Hz, 1H), 7.61 – 7.52 (m, 3H), 7.49 (d, *J* = 8.6 Hz, 1H), 7.41 – 7.34 (m, 2H), 7.13 (t, *J* = 7.6 Hz, 1H), 7.06 – 6.98 (m, 2H), 6.98 – 6.92 (m, 2H), 6.89 – 6.87 (m, 2H), 6.87 – 6.82 (m, 1H), 6.82 – 6.78 (m, 2H), 6.77 – 6.72 (m, 2H), 6.72 – 6.65 (m, 2H), 6.59 – 6.55 (m, 3H), 6.55 – 6.50 (m, 1H), 6.50 – 6.42 (m, 2H), 6.18 – 6.10 (m, 2H), 3.85 – 3.77 (m, 8H), 3.21 (s, 3H), 3.14 – 3.12 (m, 3H), 2.20 – 2.19 (m, 6H), 2.18 – 2.16 (m, 6H).

^{13}C NMR (201 MHz, C_6D_6): δ 168.7, 168.2, 165.9, 165.8, 165.0, 164.9, 163.7, 163.6, 162.1, 161.6, 161.0, 160.6, 159.8, 158.8, 157.7, 154.2, 154.1, 153.9, 153.8, 153.0, 142.8, 142.7, 139.6, 138.7, 138.3, 137.8, 137.7, 135.4, 135.3, 135.1, 134.2, 133.0, 126.2, 126.2, 125.7, 125.6, 124.8, 124.7, 124.7, 124.6, 122.7, 118.8, 118.6, 118.5, 114.8, 114.8, 114.8, 114.7, 114.4, 114.2, 114.1, 113.8, 105.9, 105.8, 65.1, 65.1, 65.0, 54.7, 39.6, 39.5, 37.3, 37.1, 37.0.

HRMS (m/z, ESI): $[M]^+$ calculated for $C_{76}H_{60}N_8O_8$: 1212.4534 found, 1212.4580; Δ = 3.79 ppm.

- **Synthesis of NTPA/Napht and characterization data**

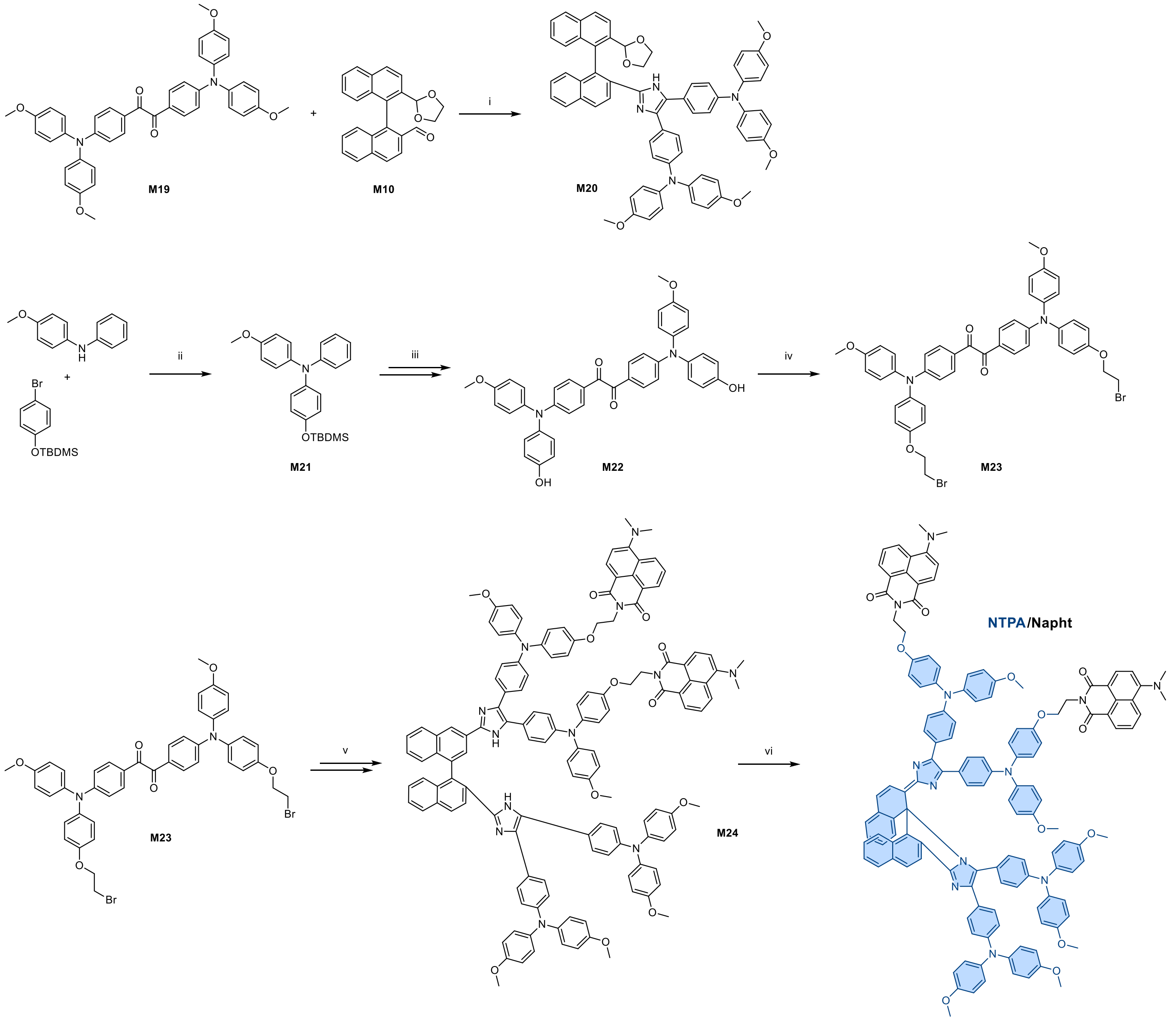

Figure S5. Synthesis of **NTPA/Napht**. *Reagents and conditions*: (i) NH_4OAc, DABCO, *t*-BuOH, 90°C, 12 h, **80%**; (ii) $Pd_2(dba)_3$, dppf, NaO*t*-Bu, toluene, 90°C, 12 h, **78%**; (iii) 1) $(COCl)_2$, $AlCl_3$, DCM, 0°C to rt, 12 h, 2) TBAF, THF, 0°C, 2 h, **48%** over two steps; (iv) 1,2-dibromoethane, KOH, 18-crown-6, toluene, 105°C, 5 h, **44%**; (v) 1) **M12**, K_2CO_3, DMF, 80°C, 12 h, 2) **M20**, NH_4OAc, AcOH, $CHCl_3$, 110°C, 12 h, **80%** over two steps; (vi) $K_3[Fe(CN)_6]$, KOH, toluene/H_2O, rt, 4 h, **4%**.

M19 – 1,2-bis(4-(bis(4-methoxyphenyl)amino)phenyl)ethane-1,2-dione

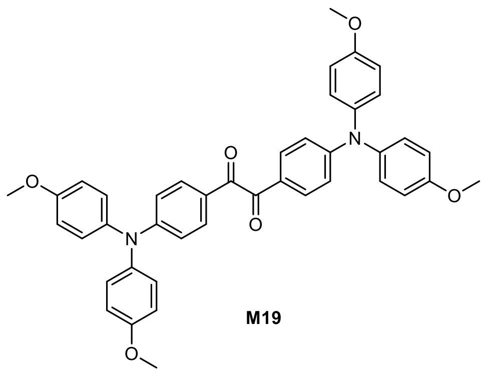

M19

M19 was prepared following a procedure reported in reference 7, without any changes. More concretely, A suspension of $AlCl_3$ (0.83 mmol) in dry dichloromethane (3 mL) was slowly added to a solution of 4-methoxy-*N*-(4-

methoxyphenyl)-*N*-phenylaniline (1.65 mmol) and oxalyl chloride (0.83 mmol) in dry dichloromethane (1.5 mL) at 0 °C. The reaction mixture was stirred at ambient temperature overnight and then quenched with ice and concentrated HCl aq. After stirring for another 1 h, the organic layer was passed through a phase separator paper. After the solvent was removed, the crude mixture was purified by silica gel column chromatography (ethyl acetate/dichloromethane/hexane: 3/20/20), to give the desired product as a yellow solid. All characterization data were fully consistent with the literature.[7]

^{1}H NMR (400 MHz, $CDCl_3$): δ 7.72 (d, *J* = 9.0 Hz, 2H), 7.63 (d, *J* = 8.8 Hz, 2H), 7.13–7.10 (m, 8H), 6.89–6.83 (m, 10H), 6.78 (d, *J* = 9.0 Hz, 4H), 3.81 (s, 12H). (*From reference* 7).

M20 – 4,4'-(2-(2'-(1,3-dioxolan-2-yl)-[1,1'-binaphthalen]-2-yl)-1*H*-imidazole-4,5-diyl)bis(*N*,*N*-bis(4-methoxyphenyl)aniline)

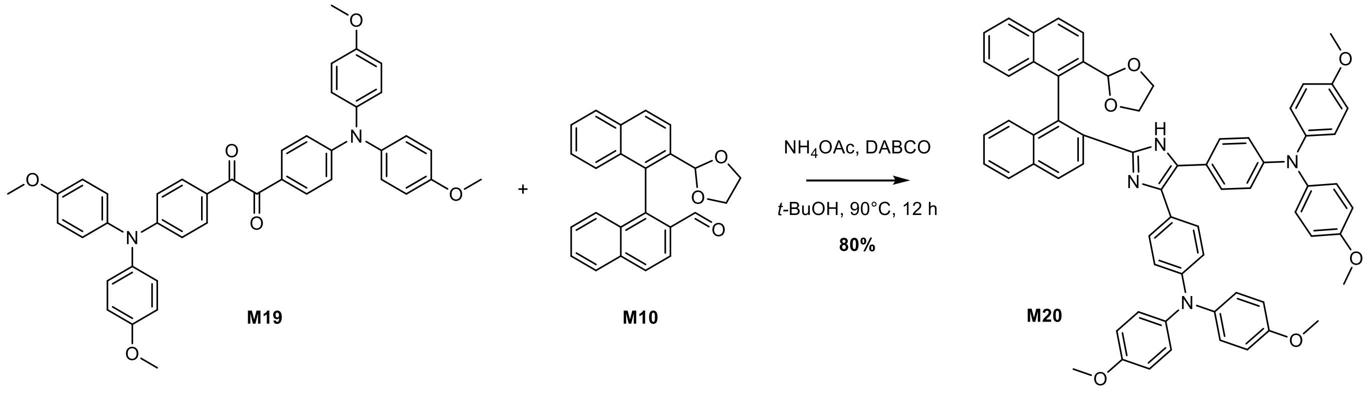

M19 (196 mg, 1.26 mmol), **M10** (83 mg, 0.23 mmol), ammonium acetate (709 mg, 9.20 mmol), and DABCO (18.4 mg, 0.16 mmol) were added to 15 mL of tert-butyl alcohol and placed under nitrogen. The mixture was heated to 90°C overnight. Then, the reaction was cooled to room temperature and was poured into 50 mL water and extracted with EtOAc (3 × 30 mL). The combined organic layers were dried over $MgSO_4$, filtered and solvents were removed under vacuum. Crude product was purified by flash column chromatography (Sfär Silica D Duo 60 µm, pentane to pentane/B 7:3, with B: EtOAc/EtOH 3:1) to give **M20** as pale yellow solid (820 mg, 91% yield).

^{1}H NMR (600 MHz, DMSO-*d$_6$*): δ 11.16 (s, 1H), 8.23 (d, *J* = 8.7 Hz, 1H), 8.16 (d, *J* = 8.7 Hz, 1H), 8.09 (d, *J* = 8.7 Hz, 1H), 8.04 (d, *J* = 8.4 Hz, 1H), 7.98 (d, *J* = 8.4 Hz, 1H), 7.82 (d, *J* = 8.7 Hz, 1H), 7.51 (ddd, *J* = 8.0, 6.8, 1.2 Hz, 1H), 7.44 (ddd, *J* = 8.0, 6.7, 1.2 Hz, 1H), 7.31 (td, *J* = 8.2, 6.8, 1.3 Hz, 1H), 7.20 (ddd, *J* = 8.2, 6.7, 1.3 Hz, 1H), 7.03 (d, *J* = 9.0 Hz, 4H), 7.01 – 6.98 (m, 4H), 6.97 (d, *J* = 8.7 Hz, 1H), 6.93 (d, *J* = 9.0 Hz, 4H), 6.91 (d, *J* = 8.9 Hz, 4H), 6.89 – 6.84 (m, 5H), 6.67 (d, *J* = 8.8 Hz, 2H), 6.53 (d, *J* = 8.8 Hz, 2H), 5.44 (s, 1H), 4.03 – 3.93 (m, 2H), 3.73 (s, 6H), 3.73 (s, 6H), 3.71 – 3.59 (m, 2H).

^{13}C NMR (151 MHz, DMSO-*d$_6$*): δ 156.0, 155.4, 147.8, 146.4, 144.0, 140.3, 139.6, 136.3, 135.6, 134.1, 133.6, 133.2, 132.5, 132.3, 131.1, 128.3, 128.0, 127.4, 127.1, 126.8, 126.4, 126.2, 125.9, 125.6, 123.6, 121.9, 119.3, 118.3, 115.0, 114.9, 101.3, 65.1, 55.2, 55.2, 39.9, 39.8, 39.7, 39.5, 39.4, 39.2, 39.1.

HRMS (m/z, ESI): $[M+H]^+$ calculated for $C_{66}H_{55}N_4O_6$: 999.4121, found 999.4104; Δ = -2.00 ppm.

M21 – 4-((*tert*-butyldimethylsilyl)oxy)-*N*-(4-methoxyphenyl)-*N*-phenylaniline

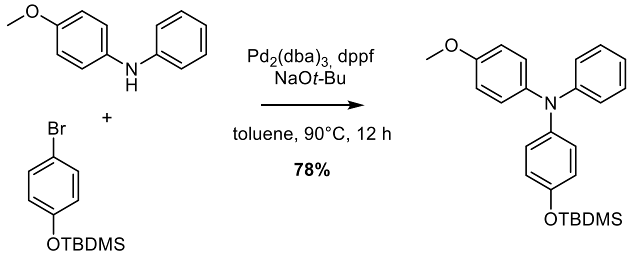

M21

N-(4-methoxyphenyl)aniline (1 g, 5.02 mmol), $Pd_2(dba)_3$ (220 mg, 0.24 mmol), dppf (319 mg, 0.44 mmol) and sodium tert-butoxide (723 mg, 7.53 mmol) were placed in a vessel and then placed under nitrogen. Toluene (12 mL) was added and 1-bromo-4-[[(1,1-dimethylethyl)dimethylsilyl]oxy]benzene (1.5 mL, 6.2 mmol) was poured to the resulting solution. The mixture was stirred at 90°C in a sealed vessel overnight. The reaction mixture was cooled down to room temperature, filtered through celite and washed with DCM. Solvents were removed under vacuum. The crude product was purified by column chromatography (Sfär Silica D Duo 60 µm, pentane/DCM 4:1) to give **M21** as a colorless oil (1.58 g, 78% yield).

^{1}H NMR (600 MHz, $CDCl_3$): δ 7.17 (dd, *J* = 8.7, 7.3 Hz, 2H), 7.04 (d, *J* = 9.0 Hz, 2H), 6.96 (d, *J* = 8.8 Hz, 2H), 6.95 – 6.92 (m, 2H), 6.89 – 6.85 (m, 1H), 6.82 (d, *J* = 9.0 Hz, 2H), 6.74 (d, *J* = 8.8 Hz, 2H), 3.79 (s, 3H), 0.99 (s, 9H), 0.20 (s, 6H).

^{13}C NMR (151 MHz, $CDCl_3$): δ 155.8, 151.6, 148.9, 141.7, 141.3, 129.2, 129.0, 126.6, 126.2, 121.2, 120.8, 120.7, 114.8, 55.6, 25.8, 18.3.

HRMS (m/z, ESI): $[M+H]^+$ calculated for $C_{25}H_{32}NO_2Si$: 406.2202, found 406.2205; Δ = 0.74 ppm.

M22 – 1,2-bis(4-((4-hydroxyphenyl)(4-methoxyphenyl)amino)phenyl)ethane-1,2-dione

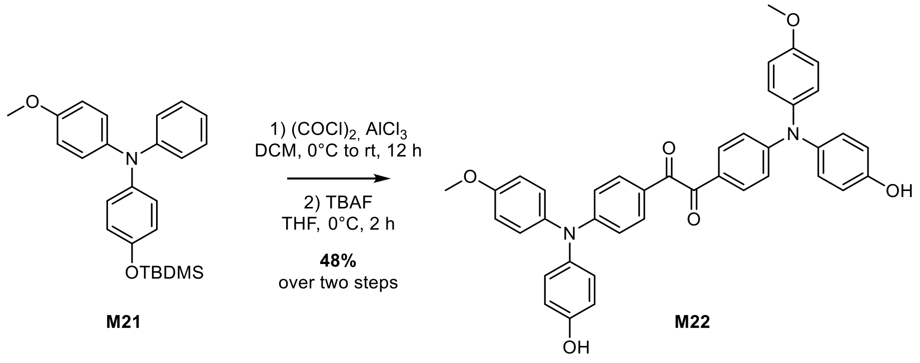

M21 **M22**

To a solution of aluminum chloride (297 mg, 2.23 mmol) in DCM (20 mL) cooled at 0°C, **M21** (1.13 g, 2.79 mmol) and oxalyl chloride (120 µL, 1.39 mmol) were added. The reaction mixture was stirred overnight and allowed to warm to room temperature. The resulting solution was quenched with ice and 1M HCl (20 mL) and extracted with DCM (3 × 30 mL). The combined organic layers were dried over $MgSO_4$, filtered and solvents were removed under vacuum. The resulting crude was dissolved in 20 mL THF and cooled to 0°C. Then, 1 M TBAF solution in THF (7 mL) was added and reaction mixture was stirred for 2 hours. Then, the reaction was warmed to room temperature and was poured into 50 mL H_2O and extracted with EtOAc (3 × 30 mL). The combined organic layers were dried over $MgSO_4$, filtered and solvents were removed under vacuum. Crude product was purified by column chromatography (Sfär Silica D Duo 60 µm, DCM to DCM/MeOH (97/3)) to give **M22** as an orange powder (423 mg, 48% yield over two steps).

^{1}H NMR (600 MHz, DMSO-*d*$_6$): δ 9.60 (s, 2H), 7.58 (d, *J* = 8.9 Hz, 4H), 7.20 (d, *J* = 8.9 Hz, 4H), 7.09 (d, *J* = 8.8 Hz, 4H), 6.98 (d, *J* = 9.0 Hz, 4H), 6.80 (d, *J* = 8.7 Hz, 4H), 6.61 (d, *J* = 9.2 Hz, 4H), 3.76 (s, 6H).

^{13}C NMR (151 MHz, DMSO-*d*$_6$): δ 193.1, 157.3, 155.8, 154.1, 137.9, 136.2, 131.4, 128.7, 128.4, 122.5, 116.6, 115.3, 114.9, 55.3.

HRMS (m/z, ESI): $[M+H]^+$ calculated for $C_{42}H_{33}N_2O_6$: 637.2338, found 637.2344; Δ = 0.94 ppm.

M23 – 1,2-bis(4-((4-(2-bromoethoxy)phenyl)(4-methoxyphenyl)amino)phenyl)ethane-1,2-dione

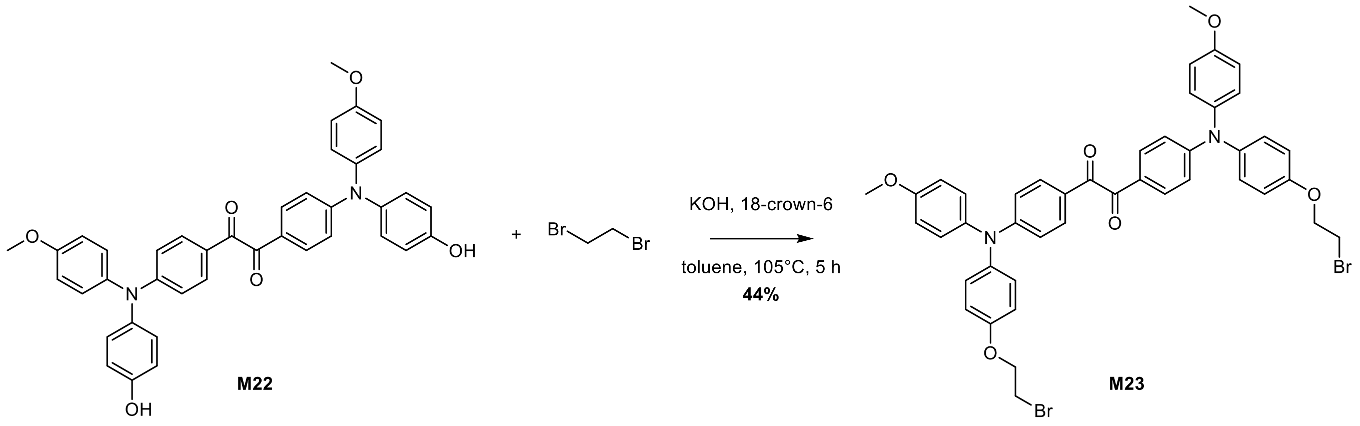

M22 (84 mg, 0.13 mmol), potassium hydroxide (509 mg, 9.29 mmol) and 18-crown-6 (14 mg, 0.053 mmol) were placed in vessel under nitrogen. Toluene (5 mL) was added, and the resulting solution was refluxed for 2 hours. Then, 1,2-dibromoethane (350 µL, 3.96 mmol) and the mixture was stirred for 2 hours. The reaction was allowed to cool to room temperature, poured into 50 mL water, and extracted with EtOAc (3 × 30 mL). The combined organic layers were dried over $MgSO_4$, filtered and solvents were removed under vacuum. The crude product was purified by column chromatography (Sfär Silica D Duo 60 µm, DCM to DCM/MeOH 95:5) to give **M23** as an orange powder (49 mg, 44% yield).

^{1}H NMR (600 MHz, DMSO-d_6): δ 7.60 (d, J = 8.8 Hz, 4H), 7.21 (d, J = 9.7 Hz, 8H), 7.01 (d, J = 9.1 Hz, 4H), 6.99 (d, J = 8.8 Hz, 4H), 6.66 (d, J = 9.1 Hz, 4H), 4.34 – 4.30 (m, 4H), 3.81 – 3.79 (m, 4H), 3.76 (s, 6H).

^{13}C NMR (151 MHz, DMSO-d_6): δ 193.1, 157.4, 156.0, 153.9, 138.3, 137.8, 131.5, 128.6, 128.5, 122.8, 116.0, 115.3, 115.3, 68.0, 55.3, 31.5.

HRMS (m/z, ESI): $[M+H]^+$ calculated for $C_{44}H_{39}Br_2N_2O_6$: 849.1175, found 849.1185; Δ = 1.18 ppm.

M24 – 2,2'-((((((2-(2'-(4,5-bis(4-(bis(4-methoxyphenyl)amino)phenyl)-1*H*-imidazol-2-yl)-[1,1'-binaphthalen]-3-yl)-1*H*-imidazole-4,5-diyl)bis(4,1-phenylene))bis((4-methoxyphenyl)azanediyl))bis(4,1-phenylene))bis(oxy))bis(ethane-2,1-diyl))bis(6-(dimethylamino)-1*H*-benzo[*de*]isoquinoline-1,3(2*H*)-dione)

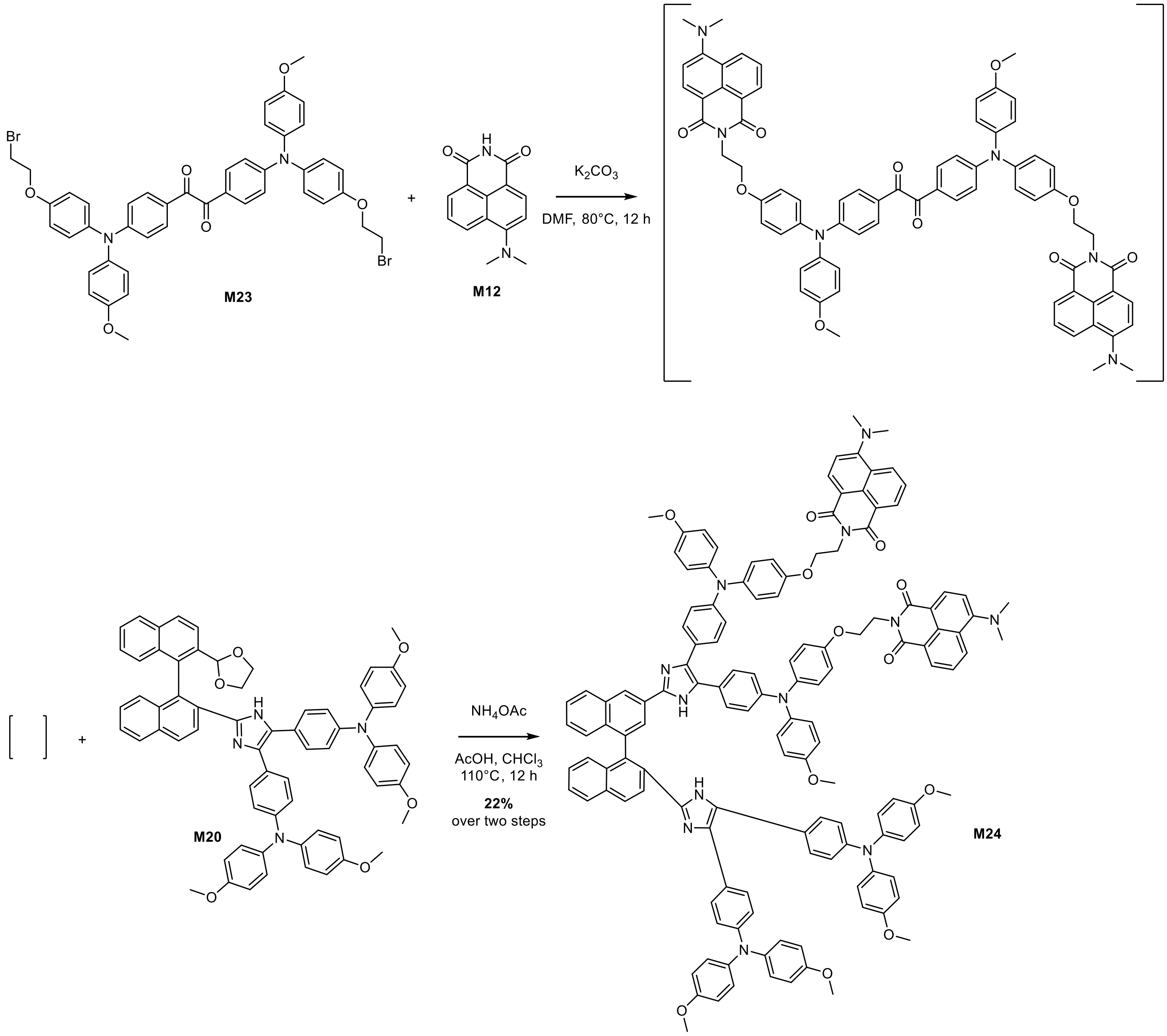

M23 (50 mg, 0.059 mmol), **M12** (40 mg, 0.17 mmol) and potassium carbonate (40.6 mg, 0.29 mmol) were added to 5 mL of DMF and placed under nitrogen. The mixture was heated to 80°C for 12 h and allowed to cool to room temperature. The resulting solution was poured in water and extracted with EtOAc (3 × 30 mL). The combined organic layers were dried over $MgSO_4$, filtered and solvents were removed under vacuum.

To the crude **M20** (61 mg, 0.05 mmol) and ammonium acetate (350 mg, 4.54 mmol) were dissolved in 3 mL of AcOH and 3 mL of $CHCl_3$. The resulting solution was placed under nitrogen and the vessel was sealed. The mixture was heated to 110°C for 12 h. Then, the reaction was cooled to room temperature and was poured into 20 mL of ammonia and extracted with DCM (3 × 30 mL). The combined organic layers were dried over $MgSO_4$, filtered and solvents were removed under vacuum. The crude product was purified by column chromatography (Sfär Silica D Duo 60 µm, toluene to toluene/B 4:1, with B: EtOAc/EtOH 3:1) to give **M24** as a yellow solid (24 mg, 22% over two steps).

^{1}H NMR (600 MHz, DMSO-*d$_6$*): δ 13.75 (s, 1H), 13.72 (s, 1H), 8.47 – 8.39 (m, 4H), 8.30 (d, *J* = 8.3 Hz, 2H), 8.11 (d, *J* = 8.6 Hz, 2H), 7.98 (d, *J* = 8.2 Hz, 2H), 7.92 (dd, *J* = 8.5, 3.2 Hz, 2H), 7.68 (t, *J* = 7.9 Hz, 2H), 7.43 (t, *J* = 7.6 Hz, 2H), 7.23 (t, *J* = 7.8 Hz, 2H), 7.13 (dd, *J* = 8.4, 4.7 Hz, 2H), 7.11 – 7.07 (m, 2H), 7.05 (d, *J* = 8.6 Hz, 2H), 7.03 – 6.95 (m, 4H), 6.94 – 6.75 (m, 36H), 6.54 – 6.47 (m, 4H), 6.46 – 6.38 (m, 2H), 4.40 – 4.34 (m, 4H), 4.26 – 4.11 (m, 4H), 3.68 – 3.64 (m, 18H), 3.02 – 2.98 (m, 12H).

^{13}C NMR (151 MHz, DMSO-d_6): δ 164.2, 163.5, 157.1, 156.3, 155.9, 155.3, 154.9, 151.6, 147.8, 146.8, 140.5, 140.1, 133.4, 132.8, 132.1, 131.1, 130.1, 128.6, 127.8, 127.3, 126.8, 126.1, 125.4, 124.6, 122.6, 119.7, 118.9, 116.0, 115.9, 115.4, 115.3, 113.4, 64.9, 55.6, 44.7.

HRMS (m/z, ESI): $[M+2H]^{2+}$ calculated for $C_{136}H_{112}N_{12}O_{12}$: 1051.9295, found 1051.9257; Δ = 3.61 ppm.

NTPA/Napht - 2,2'-((((((2-(8,9-bis(4-(bis(4-methoxyphenyl)amino)phenyl)-2'*H*-spiro[benzo[*e*]imidazo[2,1-*a*]isoindole-11,1'-naphthalen]-2'-ylidene)-2*H*-imidazole-4,5-diyl)bis(4,1-phenylene))bis((4-methoxyphenyl)azanediyl))bis(4,1-phenylene))bis(oxy))bis(ethane-2,1-diyl))bis(6-(dimethylamino)-1*H*-benzo[*de*]isoquinoline-1,3(2*H*)-dione)

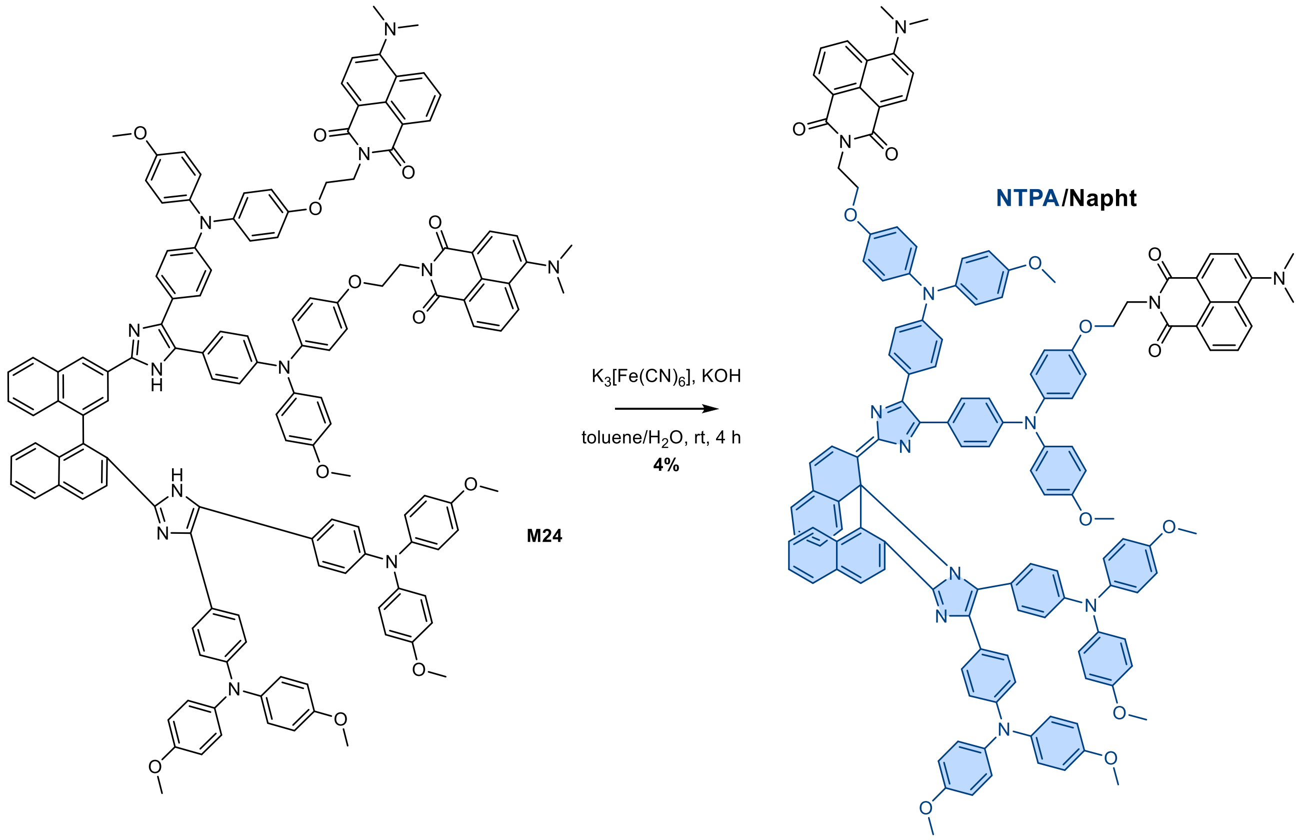

M24 (45 mg, 0.02 mmol) was dissolved in toluene (3 mL) and placed under nitrogen. A solution of potassium hydroxide (24 mg, 0.44 mmol) and potassium ferricyanide (71 mg, 0.22 mmol) in water (1 mL) was placed under nitrogen and added to the toluene solution. The reaction mixture was vigorously stirred for 4 hours at room temperature. The organic layers were separated and washed with water (3 x 20 mL) and were dried over $MgSO_4$, filtered, and the solvents were removed under vacuum. Crude product was purified by column chromatography (Sfär KP-Amino D Duo 50 µm, toluene to toluene/B 9:1, with B: EtOAc/EtOH 3:1) to give **NTPA/Napht** as dark blue solid (2 mg, 4% yield).

^{1}H NMR (600 MHz, C_6D_6): δ 8.64 – 8.58 (m, 2H), 8.58 – 8.55 (m, 1H), 8.54 – 8.51 (m, 2H), 8.43 (dd, *J* = 8.4, 4.9 Hz, 1H), 8.21 (d, *J* = 8.8 Hz, 1H), 8.17 (d, *J* = 8.8 Hz, 1H), 8.06 – 7.95 (m, 2H), 7.93 – 7.84 (m, 2H), 7.53 – 7.47 (m, 2H), 7.44 (d, *J* = 8.9 Hz, 1H), 7.36 (d, *J* = 8.3 Hz, 1H), 7.00 – 6.85 (m, 24H), 6.84 – 6.72 (m, 9H), 6.71 – 6.50 (m, 19H), 4.75 – 4.60 (m, 4H), 4.29 – 4.15 (m, 4H), 3.36 – 3.24 (m, 18H), 2.45 – 2.36 (m, 12H).

^{13}C NMR was not obtained due to low solubility of **NTPA/Napht** causing poor resolution.

HRMS (m/z, ESI): $[M+2H]^{2+}$ calculated for $C_{136}H_{110}N_{12}O_{12}$: 1051.4178, found 1051.4115; Δ = -5.99 ppm.

• Synthesis of other model compounds

Napht – 6-(dimethylamino)-2-ethyl-1*H*-benzo[*de*]isoquinoline-1,3(2*H*)-dione

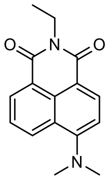

Napht

M9 was prepared following a procedure reported in reference 8. **M4** (1.24 mmol) was placed in a round bottom flask under nitrogen atmosphere and absolute EtOH (12 mL) was added. After heating to reflux, ethyl amine in THF (2M, 1.49 mmol) was added. The reaction mixture was refluxed for 2 h, until the completion of the reaction was confirmed by TLC. The solution was then allowed to cool to room temperature, and the resulting solid was collected by vacuum filtration and washed with cold methanol. **Napht** was obtained as a yellow solid. All characterization data were fully consistent with the literature.[8]

^{1}H NMR (400 MHz, $CDCl_3$): δ 8.57 (dd, *J* = 7.3, 1.2 Hz, 1H), 8.48 (d, *J* = 8.2 Hz, 1H), 8.45 (dd, *J* = 8.5, 1.1 Hz, 1H), 7.66 (dd, *J* = 8.5, 7.3 Hz, 1H), 7.13 (d, *J* = 8.2 Hz, 1H), 4.22 (q, *J* = 7.1 Hz, 2H), 3.10 (s, 5H), 1.31 (t, *J* = 7.1 Hz, 3H).

NOMe – 2'-(4,5-bis(4-methoxyphenyl)-2*H*-imidazol-2-ylidene)-8,9-bis(4-methoxyphenyl)-2'*H*-spiro[benzo[*e*]imidazo[2,1-*a*]isoindole-11,1'-naphthalene]

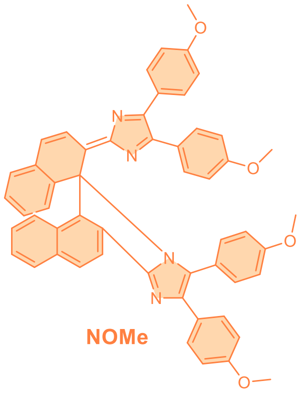

NOMe

NOMe was prepared according to a multi-step synthesis reported in reference 9. In analogy to the synthesis of **NOMe/Napht**, **NOMe** is obtained after oxidation of a bis(imidazole) intermediate. In this case, given the symmetry of the structure, the bis(imidazole) can be readily obtained by Debus-Radziszewski imidazole synthesis between **M9** and *p*-anisil. All characterization data were fully consistent with the literature.[9]

Preparation of the bis(aldehyde): see above preparation of **M9**.

Double Debus-Radziszewski reaction:

M9 (3.03 mmol), *p*-anisil (6.55 mmol), and ammonium acetate (89.2 mmol) were refluxed in acetic acid (30 mL) for 18 h. After cooling to room temperature, the reaction mixture was neutralized with aqueous NH_3. The slurry precipitate formed by neutralization was filtered off, washed with water, and then dried. This residue was purified with silica gel column chromatography using EtOAc/hexane =1/2 to 1/1 as eluent to give the corresponding bis(imidazole) precursor as a yellow powder.

^{1}H NMR (400 MHz, 296 K, DMSO-*d*$_6$): δ 3.67 (s, 3H × 2, −OMe), 3.71 (s, 3H × 2, −OMe), 6.72 (d, J = 8.0 Hz, 2H × 2), 6.83 (d, J = 8.0 Hz, 2H × 2), 6.89 (d, J = 10.0 Hz, 1H × 2), 7.10 (d, J = 8.0 Hz, 2H × 2), 7.14 (d, J = 8.0 Hz, 2H × 2), 7.26 (t, J = 10.0 Hz, 1H × 2), 7.46 (t, J = 10.0 Hz, 1H × 2), 7.99 (d, J = 10.0 Hz, 1H × 2), 8.02 (d, J = 10.0 Hz, 1H × 2), 8.17 (d, J = 10.0 Hz, 1H × 2), 13.82 (s, 1H × 2, −NH). (*From reference 9*).

Oxidation of the bis(imidazole) precursor for the obtention of **NOMe**:

All manipulations were carried out with the exclusion of light. Under nitrogen, to a solution of the bis(imidazole) precursor (1.91 mmol) in benzene (200 mL) was added a solution of potassium ferricyanide (144 mmol) and KOH

(287 mmol) in water (150 mL), and the reaction mixture was vigorously stirred for 2 h. The organic layer was separated, exhaustively washed with water, and concentrated. The residue was purified by column chromatography (DCM to DCM/EtOAc 8:2), and **NOMe** was obtained as a dark red solid.

^{1}H NMR (400 MHz, 296 K, CD_2Cl_2): δ 3.64 (s, 3H, −OMe), 3.66 (s, 3H, −OMe), 3.72 (s, 3H, −OMe), 3.74 (s, 3H, −OMe), 6.37 (d, *J* = 8.9 Hz, 2H), 6.50 (d, *J* = 8.9 Hz, 2H), 6.64 (d, *J* = 8.9 Hz, 2H), 6.75 (d, *J* = 8.9 Hz, 2H), 6.78 (d, *J* = 8.9 Hz, 2H) 6.82 (d, *J* = 7.9 Hz, 1H), 6.85 (d, *J* = 9.8 Hz, 1H), 7.01 (ddd, *J* = 7.9, 6.1, 2.1 Hz, 1H), 7.13−7.25 (m, 4H), 7.33 (d, *J* = 8.9 Hz, 2H), 7.34−7.39 (m, 1H), 7.38 (d, *J* = 8.9 Hz, 2H), 7.41 (d, *J* = 8.9 Hz, 2H), 7.74 (d, *J* = 7.9 Hz, 1H), 7.82 (d, *J* = 9.8 Hz, 1H), 7.88 (d, *J* = 8.4 Hz, 1H), 8.10 (d, *J* = 8.4 Hz, 1H). (*From reference 9*).

NTPA – 4,4'-(2-(8,9-bis(4-(bis(4-methoxyphenyl)amino)phenyl)-2'*H*-spiro[benzo[*e*]imidazo[2,1-*a*]isoindole-11,1'-naphthalen]-2'-ylidene)-2*H*-imidazole-4,5-diyl)bis(*N*,*N*-bis(4-methoxyphenyl)aniline)

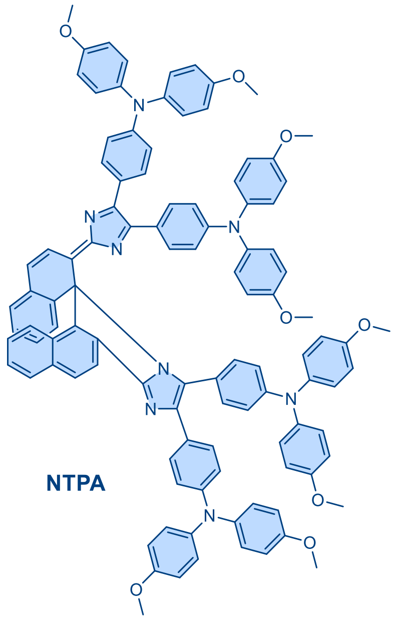

NTPA

NTPA was prepared according to a multi-step synthesis reported in reference 7. In analogy to the synthesis of **NOMe/Napht**, **NTPA** is obtained after oxidation of the bis(imidazole) intermediate. In this case, given the symmetry of the structure, the bis(imidazole) can be readily obtained by Debus-Radziszewski imidazole synthesis between **M9** and **M19**. All characterization data were fully consistent with the literature.[7]

Preparation of the bis(aldehyde): see above preparation of **M9**.

Preparation of the triphenylamino diketone derivative: see above preparation of **M19**.

Double Debus-Radziszewski reaction between **M9** *and* **M19***:*

M9 (0.068 mmol), **M19** (0.15 mmol), and ammonium acetate (3.68 mmol) were refluxed in AcOH (5 mL) for 15 h. After cooling to room temperature, the reaction mixture was neutralized with aqueous NH_3 and extracted with chloroform. The combined organic layer was washed with water and brine and passed through a phase separator paper. After the solvent was removed, the crude mixture was purified by triethyl amine treated silica gel column chromatography (EtOAc/DCM/hexane = 3/10/10), to give the desired bis(imidazole) as a pale yellow solid.

^{1}H NMR (400 MHz, DMSO-*d*$_6$): δ 13.74 (s, 2H), 8.12 (d, *J* = 8.5 Hz, 2H), 7.99 (d, *J* = 8.3 Hz, 2H), 7.92 (d, *J* = 8.5 Hz, 2H), 7.44 (t, *J* = 7.7 Hz, 2H), 7.24 (t, *J* = 7.7 Hz, 2H), 7.08 (d, *J* = 8.7 Hz, 4H), 7.01–6.81 (m, 38H), 6.59–6.53 (m, 8H), 3.70 (s, 12H), 3.69 (s, 12H). (*From reference 7*).

Oxidation of the bis(imidazole) precursor for the obtention of **NTPA**:

To a solution of the bis(imidazole) precursor obtained in the last reaction (0.017 mmol) in benzene (2 mL), it was added a solution of potassium ferricyanide (0.51 mmol) and KOH (1.3 mmol) in water (2.5 mL). The reaction mixture was vigorously stirred overnight. The organic layer was separated, exhaustively washed with water, and passed through a phase separator paper. After the solvent was removed, the crude mixture was purified by triethyl amine treated silica gel column chromatography (THF/benzene = 1/200 → 1/40), to give **NTPA** as a dark blue solid.

^{1}H NMR (400 MHz, DMSO-d_6): δ 8.10 (d, *J* = 8.6 Hz, 1H), 8.01 (d, *J* = 8.6 Hz, 1H), 7.94 (d, *J* = 7.8 Hz, 1H), 7.86 (d, *J* = 9.8 Hz, 1H), 7.44 (d, *J* = 7.5 Hz, 1H), 7.37–7.26 (m, 10H), 7.21 (d, *J* = 10.0 Hz, 1H), 7.09 (t, *J* = 7.8 Hz, 10H), 6.99–6.87 (m, 26H), 6.70 (d, *J* = 7.8 Hz, 1H), 6.62–6.59 (m, 4H), 8.50 (d, *J* = 9.0 Hz, 1H), 6.40 (d, *J* = 8.6 Hz, 1H), 6.17 (d, *J* = 8.6 Hz, 1H), 3.73 (m, 18H), 3.72 (s, 6H). (*From reference 7*).

2. Optical spectroscopy

- **UV/vis and fluorescence spectra**

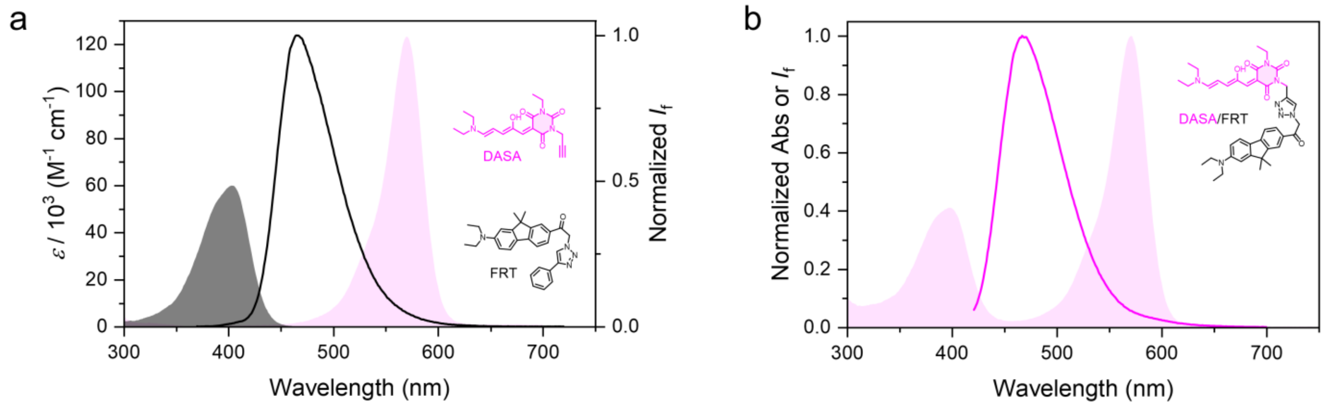

Figure S6. Absorption and emission spectra of model compounds **FRT** and **DASA** vs. dyad **DASA/FRT**. (a) Absorption (filled areas) and emission (solid line, excitation at 365 nm) spectra in air-equilibrated toluene solutions of model compounds **DASA** (magenta) and **FRT** (black). (b) Absorption spectrum of **DASA/FRT** dyad (filled area), and emission spectrum (solid line, excitation at 405 nm) recorded in air-equilibrated toluene solution at a certain photothermal distribution upon irradiating a sample containing the colored species at 405 nm.

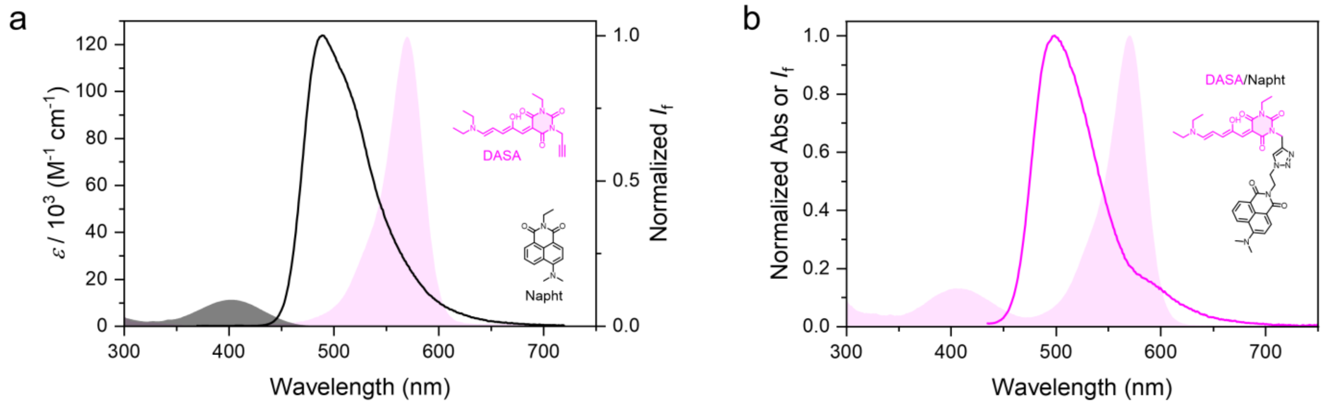

Figure S7. Absorption and emission spectra of model compounds **Napht** and **DASA** vs. dyad **DASA/Napht**. (a) Absorption (filled areas) and emission (solid line, excitation at 365 nm) spectra in air-equilibrated toluene solutions of model compounds **DASA** (magenta) and **Napht** (black). (b) Absorption spectrum of **DASA/Napht** dyad (filled area), and emission spectrum (solid line, excitation at 405 nm) recorded in air-equilibrated toluene solution at a certain photothermal distribution upon irradiating a sample containing the colored species at 405 nm.

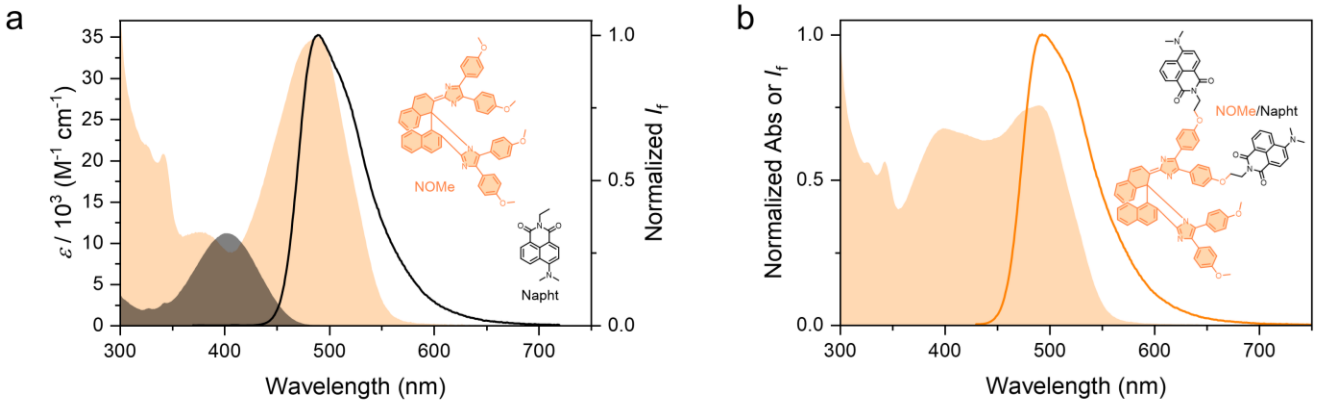

Figure S8. Absorption and emission spectra of model compounds **Napht** and **NOMe** vs. triad **NOMe/Napht**. (a) Absorption (filled areas) and emission (solid line, excitation at 365 nm) spectra in air-equilibrated toluene solutions of model compounds **NOMe** (orange) and **Napht** (black). (b) Absorption spectrum of **NOMe/Napht** triad (filled area), and emission spectrum (solid line, excitation at 405 nm) recorded in air-equilibrated toluene solution at a certain photothermal distribution upon irradiating a sample containing the colored species at 405 nm.

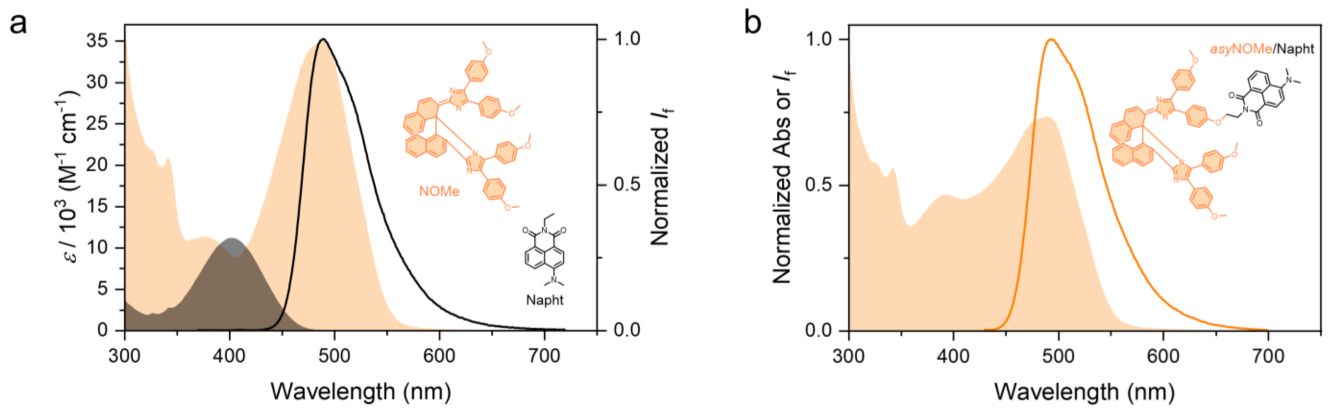

Figure S9. Absorption and emission spectra of model compounds **Napht** and **NOMe** vs. dyad ***asy*NOMe/Napht**. (a) Absorption (filled areas) and emission (solid line, excitation at 365 nm) spectra in air-equilibrated toluene solutions of model compounds **NOMe** (orange) and **Napht** (black). (b) Absorption spectrum of ***asy*NOMe/Napht** dyad (filled area), and emission spectrum (solid line, excitation at 405 nm) recorded in air-equilibrated toluene solution at a certain photothermal distribution upon irradiating a sample containing the colored species at 405 nm.

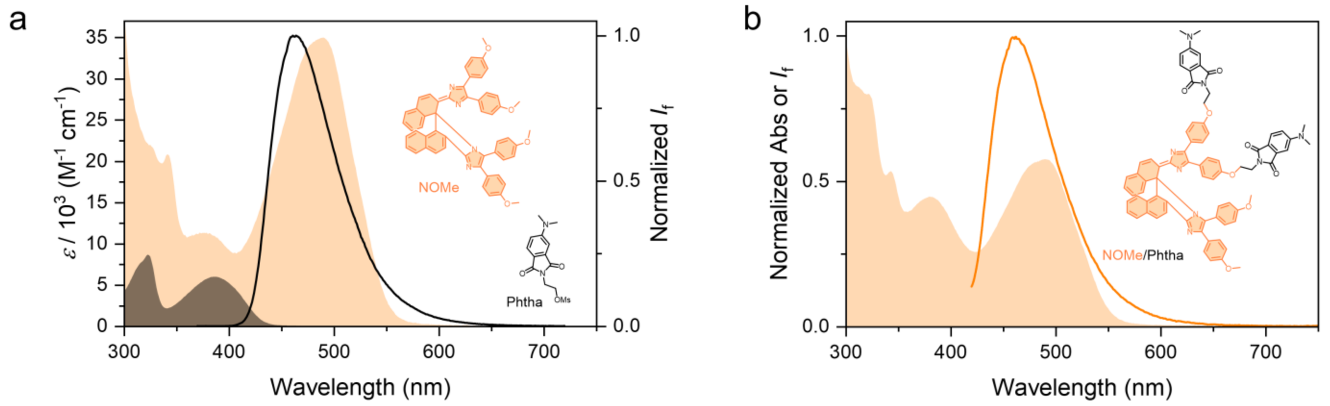

Figure S10. Absorption and emission spectra of model compounds **Napht** and **Phtha** vs. triad **NOMe/Phtha**. (a) Absorption (filled areas) and emission (solid line, excitation at 365 nm) spectra in air-equilibrated toluene solutions of model compounds **NOMe** (orange) and **Phtha** (black). (b) Absorption spectrum of **NOMe/Phtha** triad (filled area), and emission spectrum (solid line, excitation at 405 nm) recorded in air-equilibrated toluene solution at a certain photothermal distribution upon irradiating a sample containing the colored species at 405 nm.

- **Time-correlated single-photon counting data of model compounds**

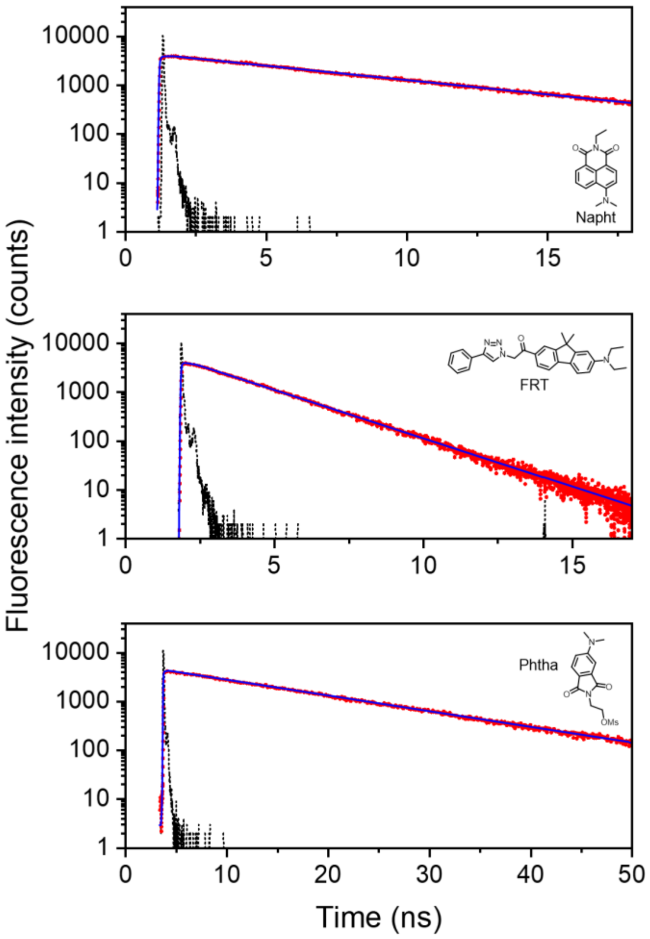

Figure S11. Fluorescence decay traces (red dotted lines) of **Napth**, **FRT**, and **Phtha** (top to bottom) in aerated toluene solution. Black and blue lines represent the instrumental response factor and the decay fittings, respectively. **Napht** and **Phtha** showed monoexponential decays with lifetimes of 7.4 and 13.4 ns, respectively. **FRT** showed a biexponential decay, with a major long-lived component of 2.1 ns (90%) and a minor short-lived component of <0.04 ns (10%). All samples were excited at 400 nm, and the fluorescence was monitored at 500 nm for **Napth** and **FRT**, and at 480 nm for **Phtha**.

- ## Two-photon absorption characterization of model compounds

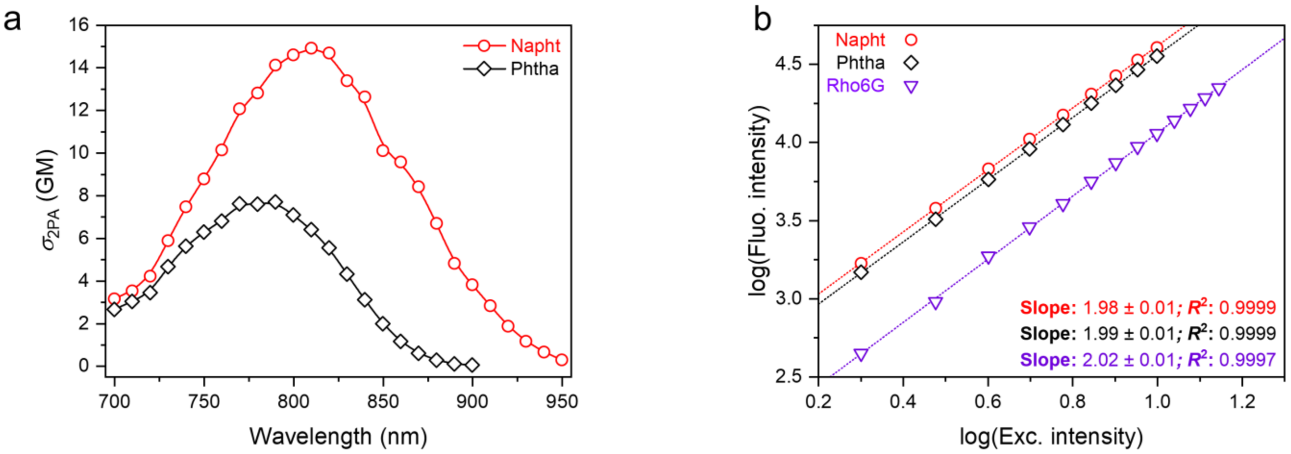

Figure S12. Characterization of the two-photon absorption properties of the model fluorophores. (a) Two-photon absorption spectra of **Napht** (red) and **Phtha** (black) in air-equilibrated toluene solution. (b) Respective double logarithmic plot of the two-photon excited fluorescence intensity vs. excitation intensity for **Napht** (red), **Phtha** (black), and **Rho6G** (purple, included as standard; methanol as solvent). The dotted lines represent the linear regression fittings.

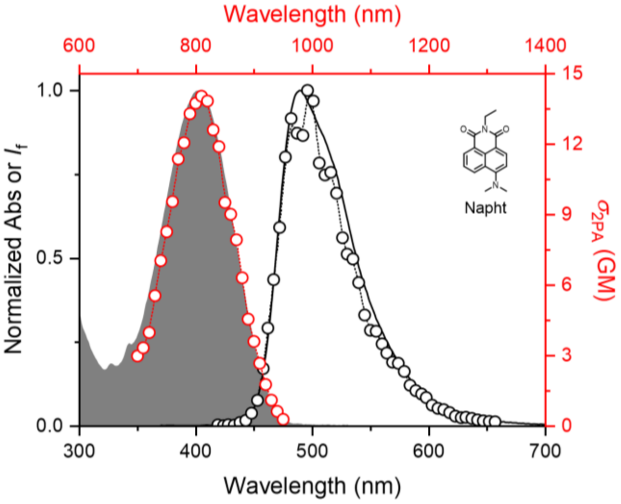

Figure S13. Comparison between absorption and emission spectra in air-equilibrated toluene of **Napht** obtained under different excitation conditions. Dark grey area and black solid line represent one-photon absorption and fluorescence spectra, respectively. The two-photon absorption (red) and the two-photon induced emission (black) spectra are represented by symbols connected by dotted lines. Fluorescence was recorded upon one-photon excitation at 380 nm or two-photon excitation at 800 nm.

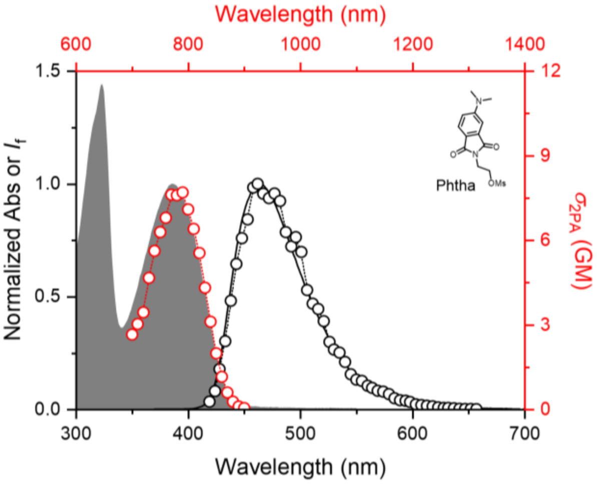

Figure S14. Comparison between absorption and emission spectra in air-equilibrated toluene of **Phtha** obtained under different excitation conditions. Dark grey area and black solid line represent one-photon absorption and fluorescence spectra, respectively. The two-photon absorption (red) and the two-photon induced emission (black) spectra are represented by symbols connected by dotted lines. Fluorescence was recorded upon one-photon excitation at 380 nm or two-photon excitation at 800 nm.

- ## Fatigue resistance studies

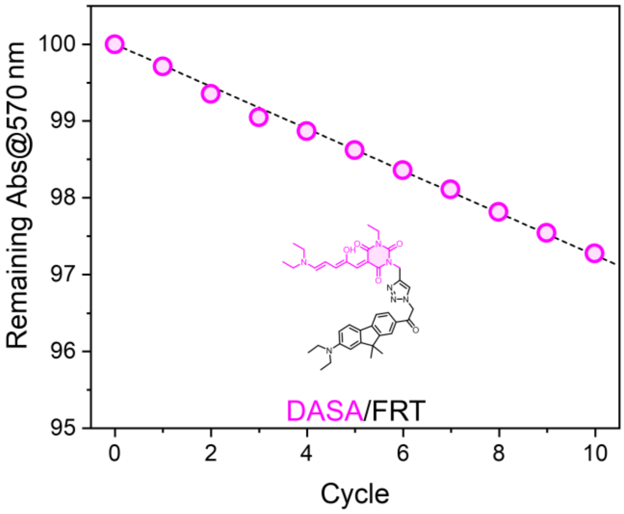

Figure S15. Fatigue resistance study for **DASA/FRT**. The remaining absorbance at the longest-wavelength absorption maximum of **DASA/FRT** is represented after several photoisomerization cycles in air-equilibrated toluene solution.

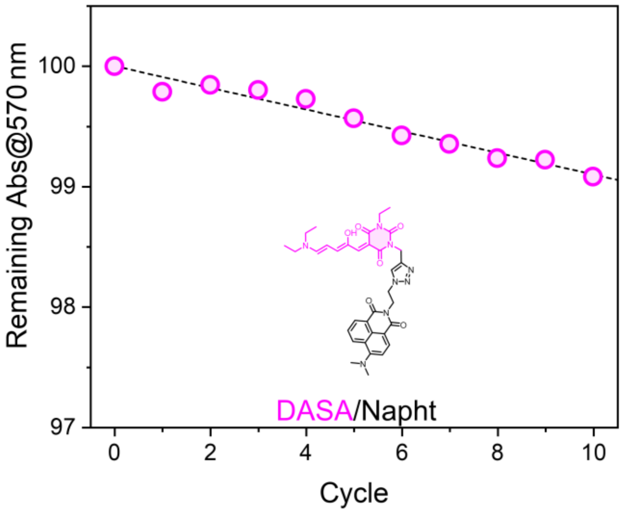

Figure S16. Fatigue resistance study for **DASA/Napht**. The remaining absorbance at the longest-wavelength absorption maximum of **DASA/Napht** is represented after several photoisomerization cycles in air-equilibrated toluene solution.

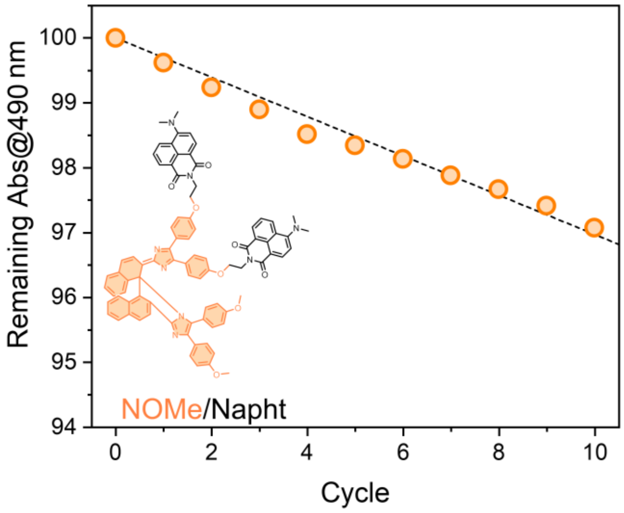

Figure S17. Fatigue resistance study for **NOMe/Napht**. The remaining absorbance at the longest-wavelength absorption maximum of **NOMe/Napht** is represented after several photoisomerization cycles in air-equilibrated toluene solution.

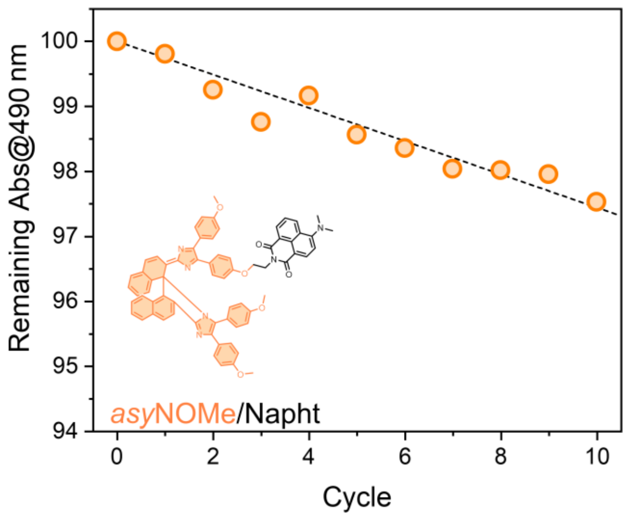

Figure S18. Fatigue resistance study for ***asy*****NOMe/Napht**. The remaining absorbance at the longest-wavelength absorption maximum of ***asy*****NOMe/Napht** is represented after several photoisomerization cycles in air-equilibrated toluene solution.

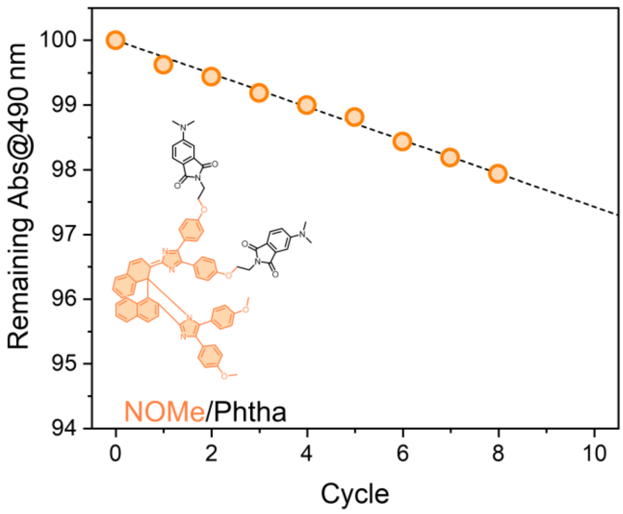

Figure S19. Fatigue resistance study for **NOMe/Phtha**. The remaining absorbance at the longest-wavelength absorption maximum of **NOMe/Phtha** is represented after several photoisomerization cycles in air-equilibrated toluene solution.

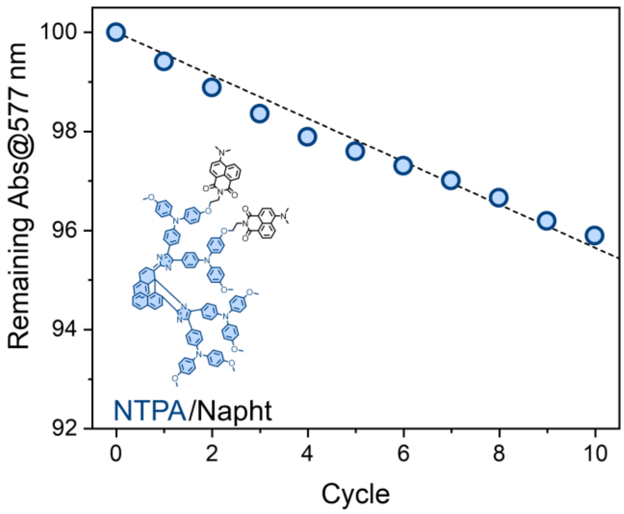

Figure S20. Fatigue resistance study for **NTPA/Napht**. The remaining absorbance at the longest-wavelength absorption maximum of **NTPA/Napht** is represented after several photoisomerization cycles in air-equilibrated toluene solution.

3. Theoretical calculations

- **Optimized structures at the ground state**

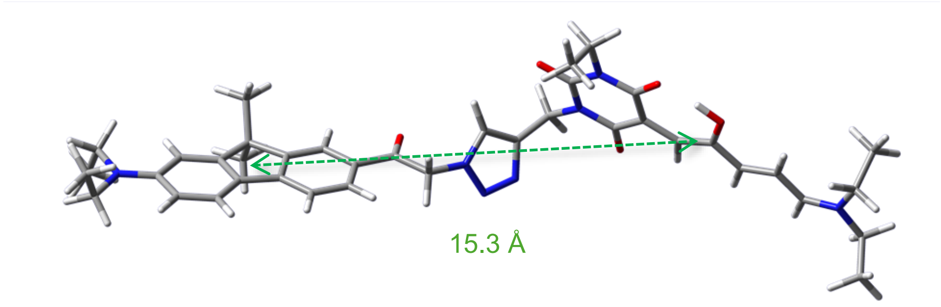

Figure S21. Optimized geometry of **DASA/FRT** at the ground state calculated at the SMD(toluene)/mPW1PW91/6-31+G(d) level of theory. The green arrow connects both donor and acceptor centers of mass. The center-to-center of mass distance is indicated. Energy: -2273.468801 Hartree. Imaginary frequencies: 0.

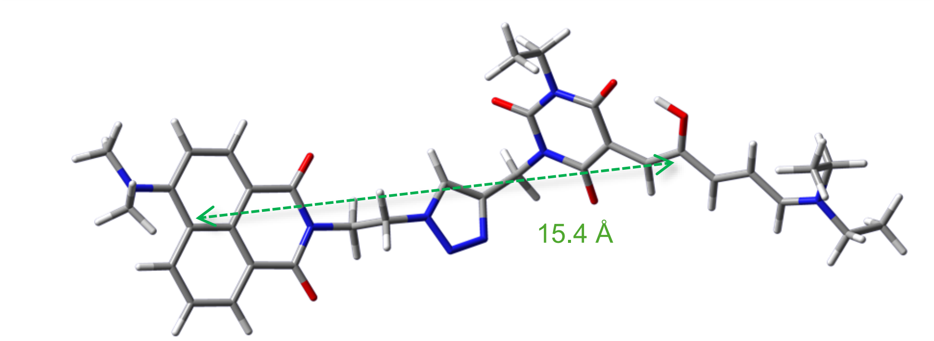

Figure S22. Optimized geometry of **DASA/Napht** at the ground state calculated at the SMD(toluene)/mPW1PW91/6-31+G(d) level of theory. The green arrow connects both donor and acceptor centers of mass. The center-to-center of mass distance is indicated. Energy: -2207.526528 Hartree. Imaginary frequencies: 0.

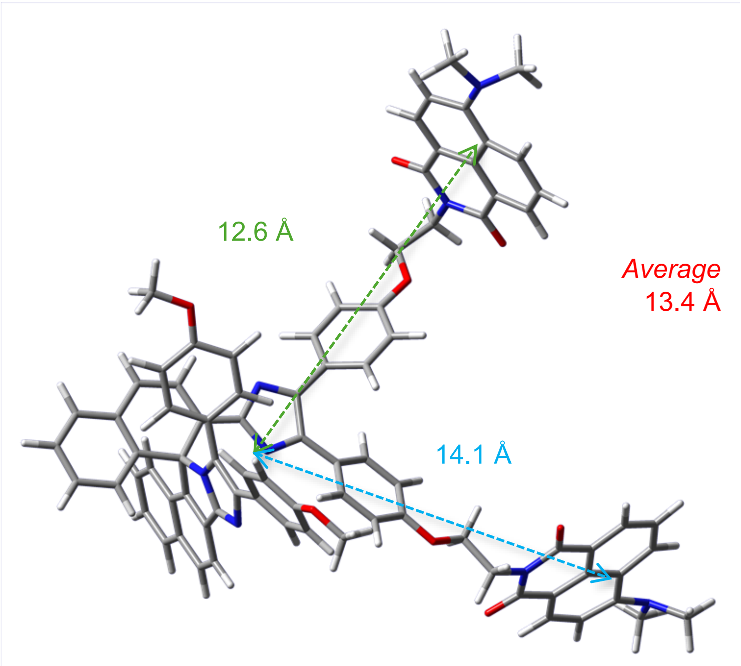

Figure S23. Optimized geometry of **NOMe/Napht** at the ground state calculated at the SMD(toluene)/mPW1PW91/6-31+G(d) level of theory. The blue and green arrows connect donors and acceptor centers of mass. The center-to-center of mass distances as well as the average donor-to-acceptor distance (red) are indicated. Energy: -4278.619700 Hartree. Imaginary frequencies: 0.

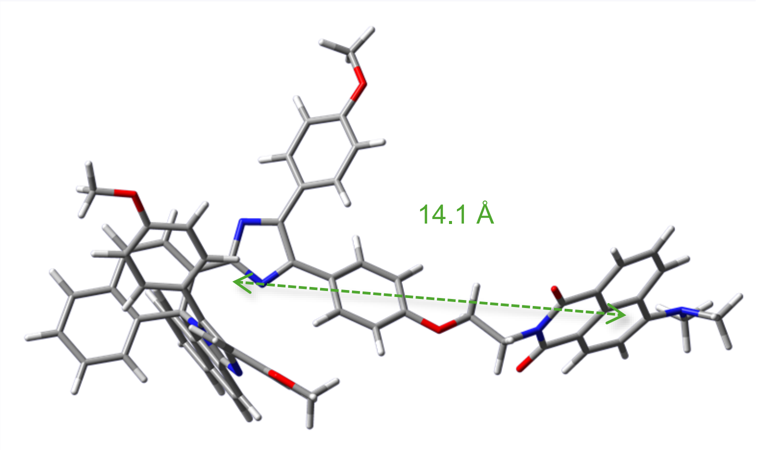

Figure S24. Optimized geometry of ***asy*NOMe/Napht** at the ground state calculated at the SMD(toluene)/mPW1PW91/6-31+G(d) level of theory. The green arrow connects both donor and acceptor centers of mass. The center-to-center of mass distance is indicated. Energy: -3439.936362 Hartree. Imaginary frequencies: 0.

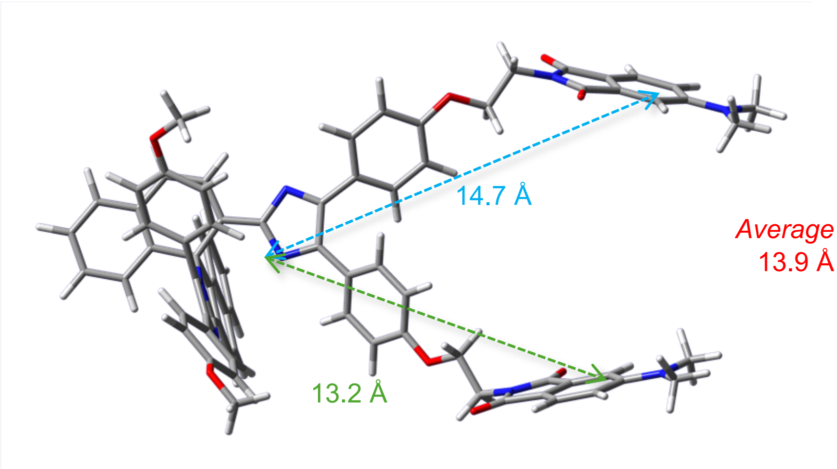

Figure S25. Optimized geometry of **NOMe/Phtha** at the ground state calculated at the SMD(toluene)/mPW1PW91/6-31+G(d) level of theory. The blue and green arrows connect donors and acceptor centers of mass. The center-to-center of mass distances as well as the average donor-to-acceptor distance (red) are indicated. Energy: -3971.383674 Hartree. Imaginary frequencies: 0.

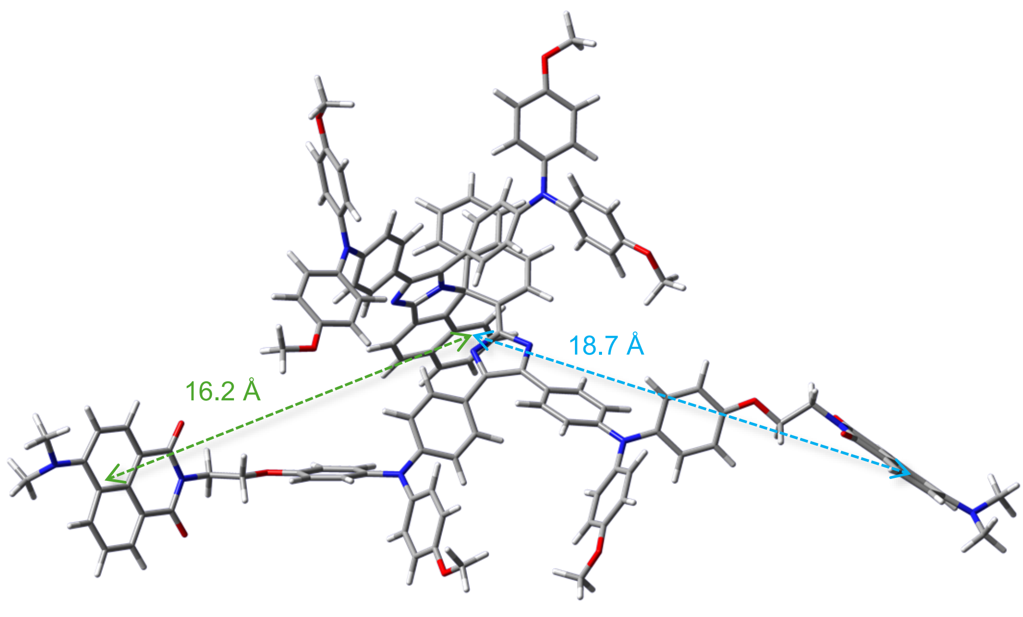

Figure S26. Optimized geometry of **NTPA/Napht** at the ground state calculated at the SMD(toluene)/mPW1PW91/6-31+G(d) level of theory. The blue and green arrows connect donors and acceptor centers of mass. The center-to-center of mass distances as well as the average donor-to-acceptor distance (red) are indicated. Energy: -6806.040246 Hartree. Imaginary frequencies: 0.

Table S1. Additional theoretical insights in FRET for dyads and triads under study.

**Compound**	***r* (Å)**[a]	***J* / 10^{15} (nm^4 M^{-1} cm^{-1})**[b]	**R_0 (Å)**[c]	**E_{FRET}**[d]
**DASA/FRT**	15.3	0.8	45 ± 2	0.9985
**DASA/Napht**	15.4	2.4	53 ± 2	0.9994
**NOMe/Napht**	13.4	1.3	48 ± 2	0.9995
***asy*NOMe/Napht**	14.1	1.3	48 ± 2	0.9993
**NOMe/Phtha**	13.9	1.3	46 ± 2	0.9991
**NTPA/Napht**	17.5	1.7	48 ± 2	0.9976

[a] FRET-donor-acceptor distance calculated at the ground state optimized geometry by SMD(toluene)/mPW1PW91/6-31+G(d). For the triads, the average donor-acceptor distance is presented.
[b] Overlap integrals calculated from the experimental spectra of the respective monomers. Typical error: 10%.
[c] Förster radius (R_0) calculated according to FRET formalism[10], using the experimental data in Table 1 and assuming the orientation factor (κ^2) to be 2/3. For the triads, the Förster radius was calculated for each donor- -acceptor pair and averaged. Errors in R_0 values were determined by propagating typical deviations of ca. 10% in the fluorescence quantum yield, integral overlap and κ^2, and of ca. 5% for the refractive index.[11]
[d] FRET efficiency calculated as $1/[1 + (r/R_0)^6]$. For the triads, the efficiency was averaged from that of each donor-to-acceptor pair.

• Additional TDDFT calculations

Table S2. Calculated electronic and photophysical data for **DASA/FRT**.

Electronic transition	*f* [a]	Assignation[b]	NTO composition (contribution[c])	NTO weight	E_{theo} (eV)[d] [λ (nm)]	E_{exp} (eV)[e] [λ (nm)]
$S_0 \rightarrow S_1$	1.5863	Acceptor (**DASA**)	HOMO→LUMO (100)	100	2.52 [491.86]	2.18 [570]
$S_0 \rightarrow S_3$	0.9279	Donor (**FRT**)	HOMO-1→LUMO+1 (100)	100	3.07 [404.02]	3.08 [403]

[a] Oscillator strength. [b] Based on NTOs visual inspection (see figure below). [c] Percentage contribution approximated by $2c_i^2 \times 100\%$. [c] Absorption energies calculated at the SMD(toluene)/mPW1PW91/6-31+G(d) level of theory. [d] Experimental absorption energies in air-equilibrated toluene solution of the model monomers composing **DASA/FRT** (*please note that the identification of the absorption bands corresponding to the components integrated onto the dyads/triads can be hampered in some cases by spectral overlap; therefore, we chose to compare the theoretical obtained values in the dyad or triad with the experimental ones from the model compounds*).

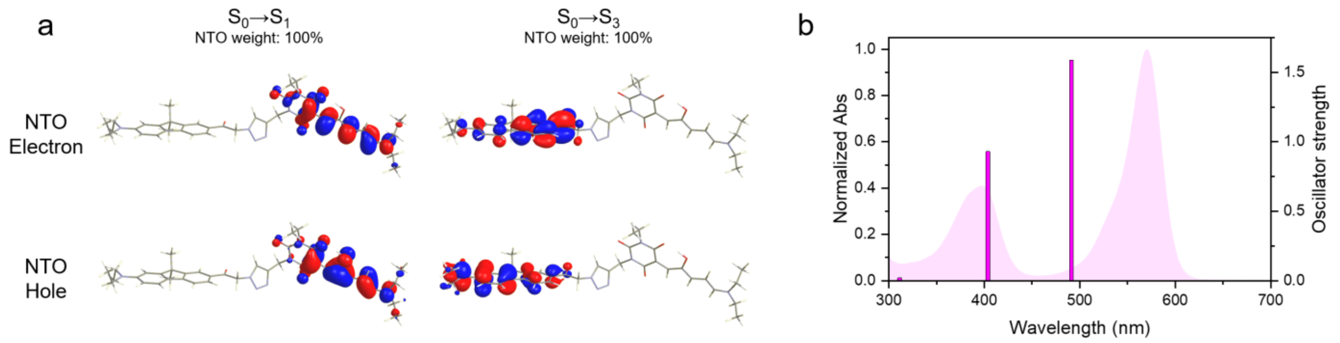

Figure S27. NTOs and calculated absorption spectrum for **DASA/FRT**. (a) Natural Transition Orbitals (NTOs) for $S_0 \rightarrow S_1$ and $S_0 \rightarrow S_3$ transitions in **DASA/FRT**. The contributions of the NTOs to the respective transitions are indicated in each case (isosurface: 0.03 e/bohr^3). (b) Experimental (fill area) and calculated (perpendicular lines) absorption spectra for **DASA/FRT**.

Table S3. Calculated electronic and photophysical data for **DASA/Napht**.

Electronic transition	*f* [a]	Assignation[b]	NTO composition (contribution[c])	NTO weight	E_{theo} (eV)[d] [λ (nm)]	E_{exp} (eV)[e] [λ (nm)]
$S_0 \rightarrow S_1$	1.5294	Acceptor (**DASA**)	HOMO→LUMO+1 (100)	100	2.52 [491.84]	2.18 [570]
$S_0 \rightarrow S_3$	0.3587	Donor (**Napht**)	HOMO-1→LUMO (100)	100	3.03 [408.71]	3.08 [402]

[a] Oscillator strength. [b] Based on NTOs visual inspection (see figure below). [c] Percentage contribution approximated by $2c_i^2 \times 100\%$. [c] Absorption energies calculated at the SMD(toluene)/mPW1PW91/6-31+G(d) level of theory. [d] Experimental absorption energies in air-equilibrated toluene solution of the model monomers composing **DASA/Napht** (*please note that the identification of the absorption bands corresponding to the components integrated onto the dyads/triads can be hampered in some cases by spectral overlap therefore, we chose to compare the theoretical obtained values in the dyad or triad with the experimental ones from the model compounds*).

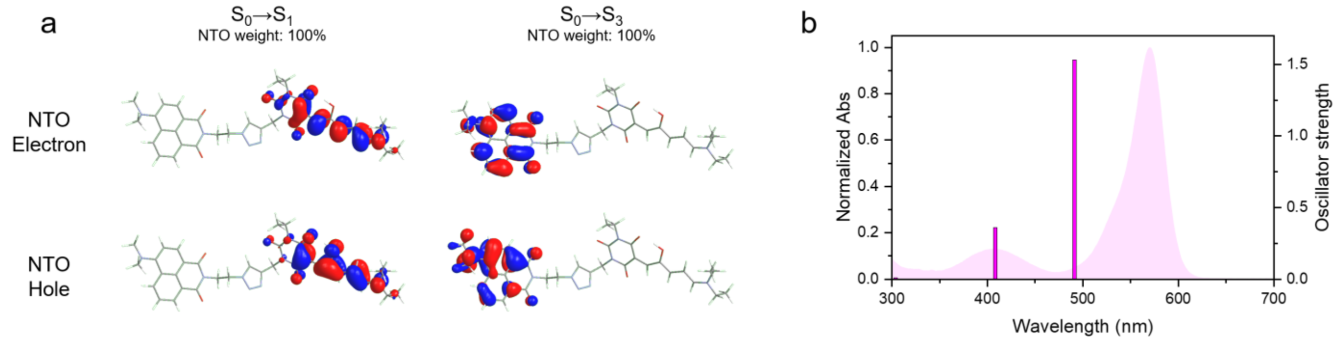

Figure S28. NTOs and calculated absorption spectrum for **DASA/Napht**. (a) Natural Transition Orbitals (NTOs) for $S_0 \rightarrow S_1$ and $S_0 \rightarrow S_3$ transitions in **DASA/Napht**. The contributions of the NTOs to the respective transitions are indicated in each case (isosurface: 0.03 e/bohr^3). (b) Experimental (fill area) and calculated (perpendicular lines) absorption spectra for **DASA/Napht**.

Table S4. Calculated electronic and photophysical data for **NOMe/Napht**.

Electronic transition	*f* [a]	Assignation[b]	NTO composition (contribution[c])	NTO weight	E_{theo} (eV)[d] [λ (nm)]	E_{exp} (eV)[e] [λ (nm)]
$S_0 \rightarrow S_2$	1.1654	Acceptor (**NOMe**)	HOMO-1→LUMO (98)	98	2.29 [541.56]	2.53 [490]
$S_0 \rightarrow S_{12}$	0.3587	Donor (**Napht**)	HOMO-3→LUMO+1 (97)	97	3.04 [408.33]	3.08 [402]
$S_0 \rightarrow S_{13}$	0.3587	Donor (**Napht**)	HOMO-2→LUMO+2 (97)	97	3.05 [406.19]	3.08 [402]

[a] Oscillator strength. [b] Based on NTOs visual inspection (see figure below). [c] Percentage contribution approximated by $2c_i^2 \times 100\%$. [c] Absorption energies calculated at the SMD(toluene)/mPW1PW91/6-31+G(d) level of theory. [d] Experimental absorption energies in air-equilibrated toluene solution of the model monomers composing **NOMe/Napht** (*please note that (i) the identification of the absorption bands corresponding to the components integrated onto the dyads/triads can be hampered in some cases by spectral overlap therefore, we chose to compare the theoretical obtained values in the dyad or triad with the experimental ones from the model compounds, and (ii) considerable experimental-theoretical differences for NBIC derivatives are observed with this level of theory when including the solvent in the calculations, as seen in ref. 7).*

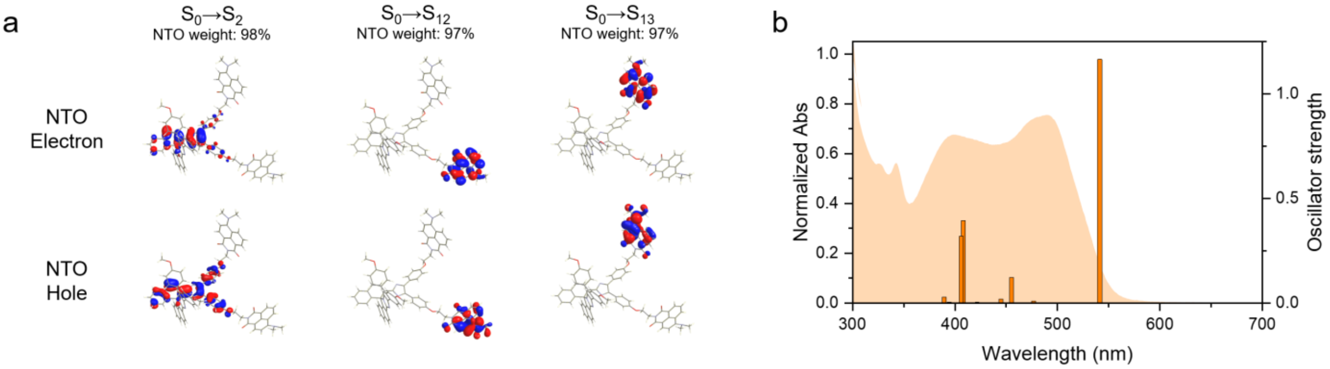

Figure S29. NTOs and calculated absorption spectrum for **NOMe/Napht**. (a) Natural Transition Orbitals (NTOs) for $S_0 \rightarrow S_2$, $S_0 \rightarrow S_{12}$ and $S_0 \rightarrow S_{13}$ transitions in **NOMe/Napht**. The contributions of the NTOs to the respective transitions are indicated in each case (isosurface: 0.03 e/bohr^3). (b) Experimental (fill area) and calculated (perpendicular lines) absorption spectra for **NOMe/Napht**.

Table S5. Calculated electronic and photophysical data for ***asy*NOMe/Napht**.

Electronic transition	*f* [a]	Assignation[b]	NTO composition (contribution[c])	NTO weight	E_{theo} (eV)[d] [λ (nm)]	E_{exp} (eV)[e] [λ (nm)]
$S_0 \rightarrow S_2$	1.1500	Acceptor (**NOMe**)	HOMO-1→LUMO (98)	98	2.29 [540.42]	2.53 [490]
$S_0 \rightarrow S_9$	0.3571	Donor (**Napht**)	HOMO-2→LUMO+1 (100)	98	3.03 [408.66]	3.08 [402]

[a] Oscillator strength. [b] Based on NTOs visual inspection (see figure below). [c] Percentage contribution approximated by $2c_i^2 \times 100\%$. [c] Absorption energies calculated at the SMD(toluene)/mPW1PW91/6-31+G(d) level of theory. [d] Experimental absorption energies in air-equilibrated toluene solution of the model monomers composing ***asy*NOMe/Napht** (*please note that the identification of the absorption bands corresponding to the components integrated onto the dyads/triads can be hampered in some cases by spectral overlap therefore, we chose to compare the theoretical obtained values in the dyad or triad with the experimental ones from the model compounds, and (ii) considerable experimental-theoretical differences for NBIC derivatives are observed with this level of theory when including the solvent in the calculations, as seen in ref. 7).*

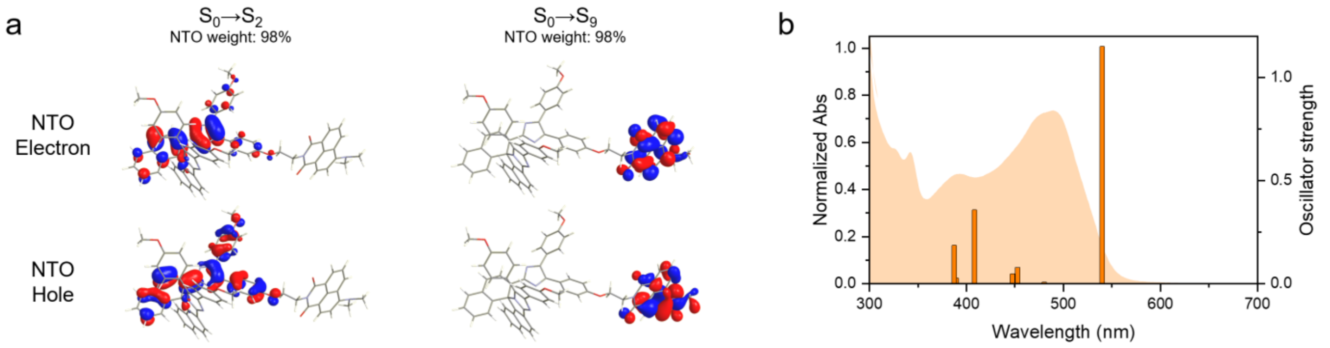

Figure S30. NTOs and calculated absorption spectrum for ***asy*NOMe/Napht**. (a) Natural Transition Orbitals (NTOs) for $S_0 \rightarrow S_2$, $S_0 \rightarrow S_9$ transitions in ***asy*NOMe/Napht**. The contributions of the NTOs to the respective transitions are indicated in each case (isosurface: 0.03 e/bohr^3). (b) Experimental (fill area) and calculated (perpendicular lines) absorption spectra for ***asy*NOMe/Napht**.

Table S6. Calculated electronic and photophysical data for **NOMe/Phtha**.

Electronic transition	*f* [a]	Assignation[b]	NTO composition (contribution[c])	NTO weight	E_{theo} (eV)[d] [λ (nm)]	E_{exp} (eV)[e] [λ (nm)]
$S_0 \rightarrow S_2$	1.1645	Acceptor (**NOMe**)	HOMO-1→LUMO (99)	99	2.30 [539.91]	2.53 [490]
$S_0 \rightarrow S_{13}$	0.0334	Donor (**Phtha**)	HOMO-3→LUMO+1 (69)<br>HOMO-2→LUMO+2 (27)	97	3.21 [385.81]	3.22 [385]
$S_0 \rightarrow S_{14}$	0.2732	Donor (**Phtha**)	HOMO-2→LUMO+2 (69)<br>HOMO-3→LUMO+1 (28)	97	3.22 [385.30]	3.22 [385]

[a] Oscillator strength. [b] Based on NTOs visual inspection (see figure below). [c] Percentage contribution approximated by $2c_i^2 \times 100\%$. [c] Absorption energies calculated at the SMD(toluene)/mPW1PW91/6-31+G(d) level of theory. [d] Experimental absorption energies in air-equilibrated toluene solution of the model monomers composing **NOMe/Phtha** (*please note that (i) the identification of the absorption bands corresponding to the components integrated onto the dyads/triads can be hampered in some cases by spectral overlap therefore, we chose to compare the theoretical obtained values in the dyad or triad with the experimental ones from the model compounds, and (ii) considerable experimental-theoretical differences for NBIC derivatives are observed with this level of theory when including the solvent in the calculations, as seen in ref. 7).*

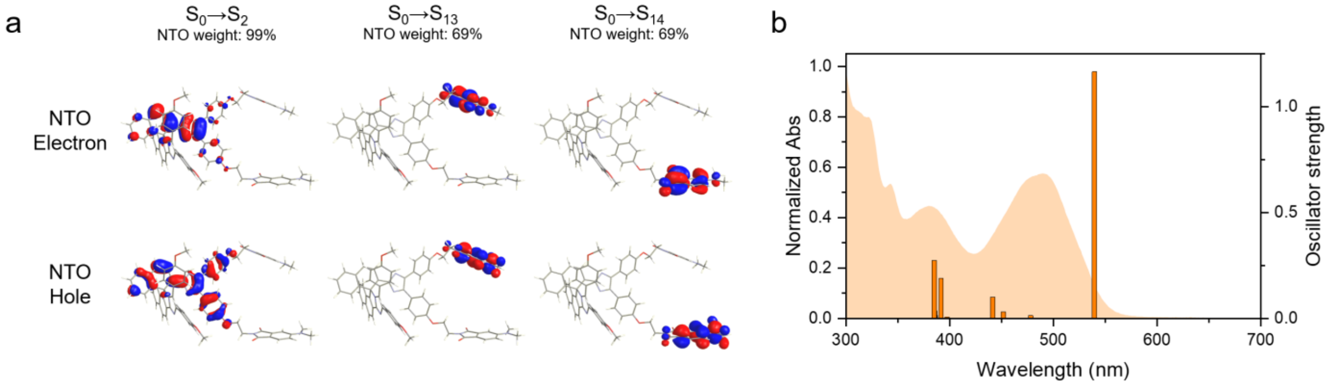

Figure S31. NTOs and calculated absorption spectrum for **NOMe/Phtha**. (a) Natural Transition Orbitals (NTOs) for $S_0 \rightarrow S_2$, $S_0 \rightarrow S_{13}$ and $S_0 \rightarrow S_{14}$ transitions in **NOMe/Phtha**. The contributions of the NTOs to the respective transitions are indicated in each case (isosurface: 0.03 e/bohr^3). (b) Experimental (fill area) and calculated (perpendicular lines) absorption spectra for **NOMe/Phtha**.

Table S7. Calculated electronic and photophysical data for **NTPA/Napht**.

Electronic transition	*f* [a]	Assignation[b]	NTO composition (contribution[c])	NTO weight	E_{theo} (eV)[d] [λ (nm)]	E_{exp} (eV)[e] [λ (nm)]
$S_0 \rightarrow S_2$	0.1170	Acceptor (**NOMe**)	HOMO-1→LUMO (70)<br>HOMO-2→LUMO (27)	97	1.72 [722.48]	2.15 [577]
$S_0 \rightarrow S_3$	0.6985	Acceptor (**NOMe**)	HOMO-2→LUMO (69)<br>HOMO-1→LUMO (27)	99	1.80 [687.43]	2.15 [577]
$S_0 \rightarrow S_4$	0.3384	Acceptor (**NOMe**)	HOMO-3→LUMO (97)	97	1.88 [658.30]	2.15 [577]
$S_0 \rightarrow S_{19}$	0.4334	Donor (**Napht**)	HOMO-7→LUMO+1 (98)	98	3.03 [409.25]	3.08 [402]
$S_0 \rightarrow S_{20}$	0.3618	Donor (**Napht**)	HOMO-6→LUMO+2 (98)	98	3.04 [407.93]	3.08 [402]

[a] Oscillator strength. [b] Based on NTOs visual inspection (see figure below). [c] Percentage contribution approximated by $2c_i^2 \times 100\%$. [c] Absorption energies calculated at the SMD(toluene)/mPW1PW91/6-31+G(d) level of theory. [d] Experimental absorption energies in air-equilibrated toluene solution of the model monomers composing **NTPA/Napht** (*please note that (i) the identification of the absorption bands corresponding to the components integrated onto the dyads/triads can be hampered in some cases by spectral overlap therefore, we chose to compare the theoretical obtained values in the dyad or triad with the experimental ones from the model compounds, and (ii) considerable experimental-theoretical differences for NBIC derivatives are observed with this level of theory when including the solvent in the calculations, as seen in ref. 7).*

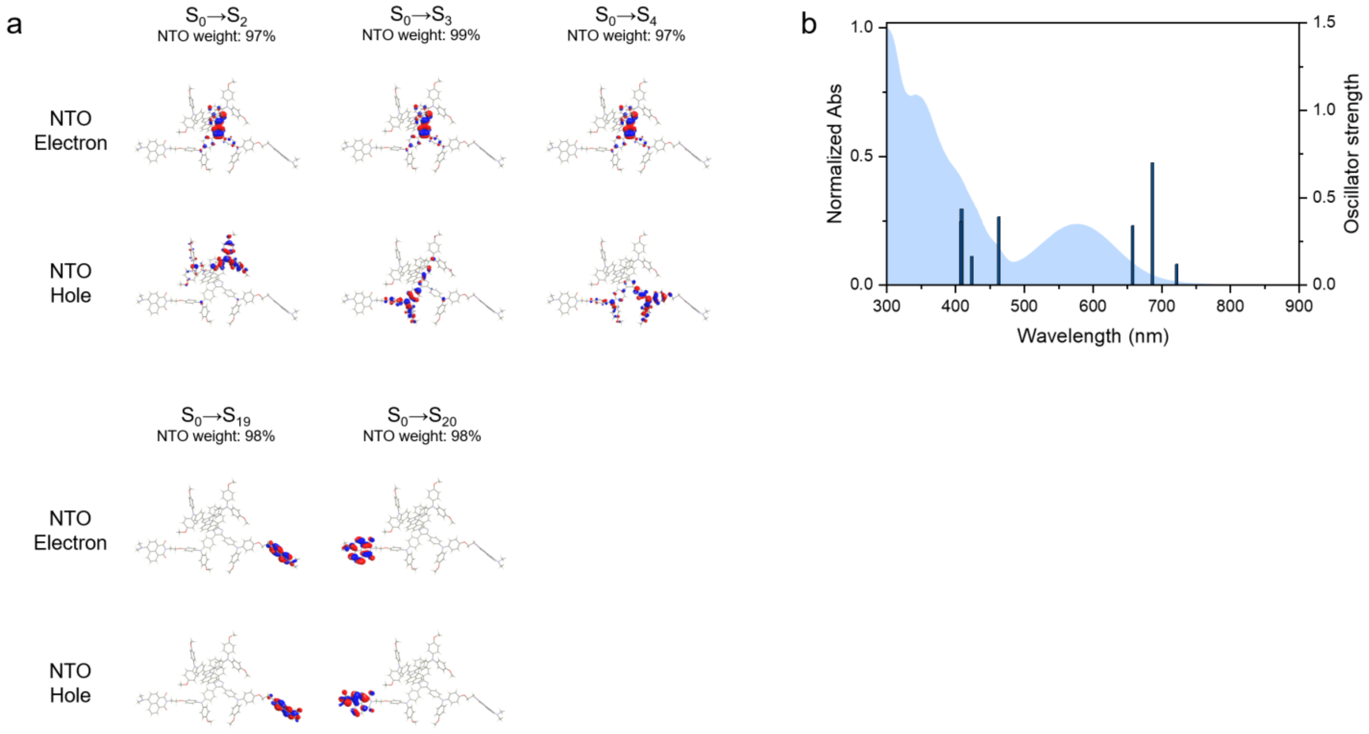

Figure S32. NTOs and calculated absorption spectrum for **NTPA/Napht**. (a) Natural Transition Orbitals (NTOs) for $S_0 \rightarrow S_2$, $S_0 \rightarrow S_3$, $S_0 \rightarrow S_4$, $S_0 \rightarrow S_{19}$ and $S_0 \rightarrow S_{20}$ transitions in **NTPA/Napht**. The contributions of the NTOs to the respective transitions are indicated in each case (isosurface: 0.03 e/bohr^3). (b) Experimental (fill area) and calculated (perpendicular lines) absorption spectra for **NTPA/Napht**.

4. FRET experimental characterization and two-photon validation studies

- **fs-UC studies for FRET characterization**

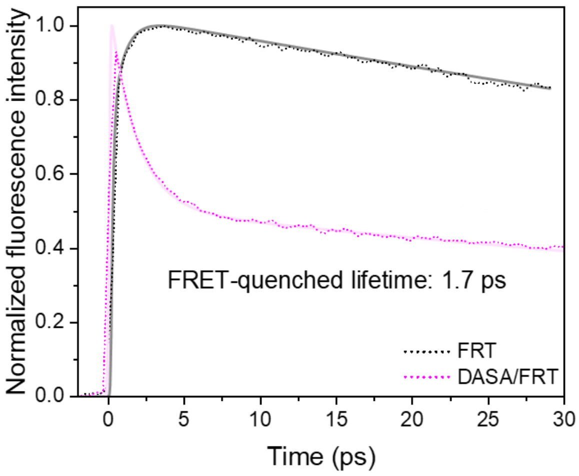

Figure S33. Comparison between the fluorescence decays (dotted lines, excitation at 400 nm and detection at 500 nm) in the ps regime of model **FRT** (black) and dyad **DASA/FRT** (magenta) in air-equilibrated toluene solution. Solid lines represent the fittings of the respective decays.

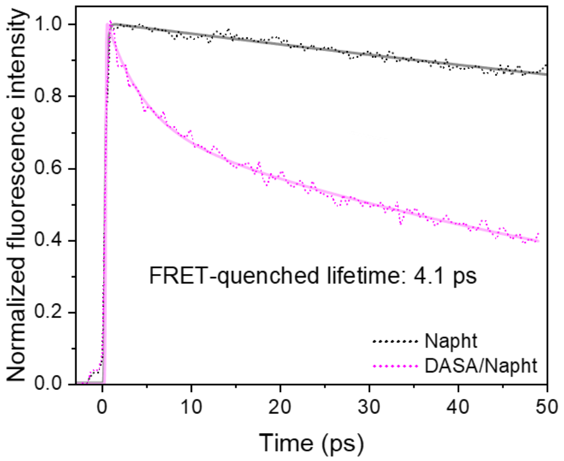

Figure S34. Comparison between the fluorescence decays (dotted lines, excitation at 400 nm and detection at 500 nm) in the ps regime of model **Napht** (black) and dyad **DASA/Napht** (magenta) in air-equilibrated toluene solution. Solid lines represent the fittings of the respective decays.

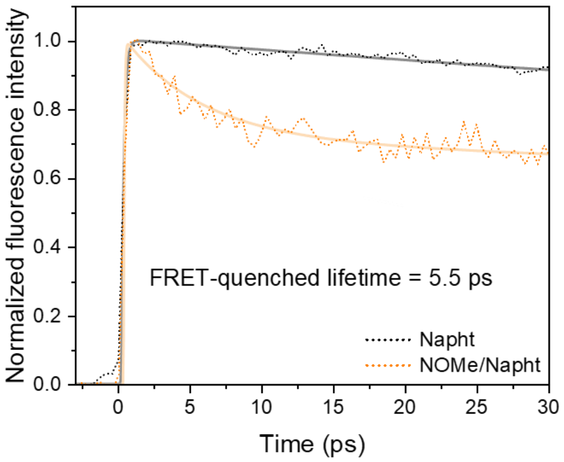

Figure S35. Comparison between the fluorescence decays (dotted lines, excitation at 400 nm and detection at 500 nm) in the ps regime of model **Napht** (black) and triad **NOMe/Napht** (orange) in air-equilibrated toluene solution. Solid lines represent the fittings of the respective decays.

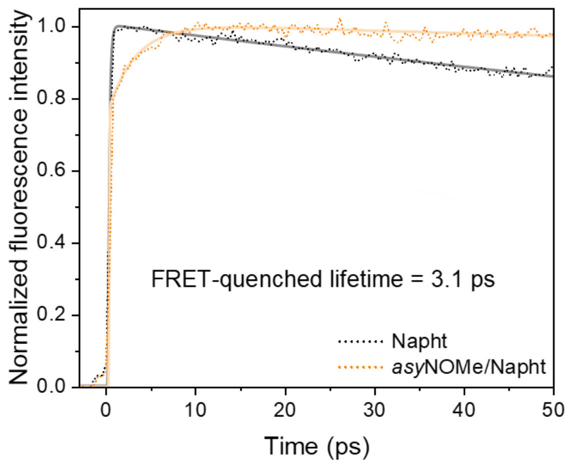

Figure S36. Comparison between the fluorescence decays (dotted lines, excitation at 400 nm and detection at 500 nm) in the ps regime of model **Napht** (black) and dyad ***asy*NOMe/Napht** (orange) in air-equilibrated toluene solution. Solid lines represent the fittings of the respective decays.

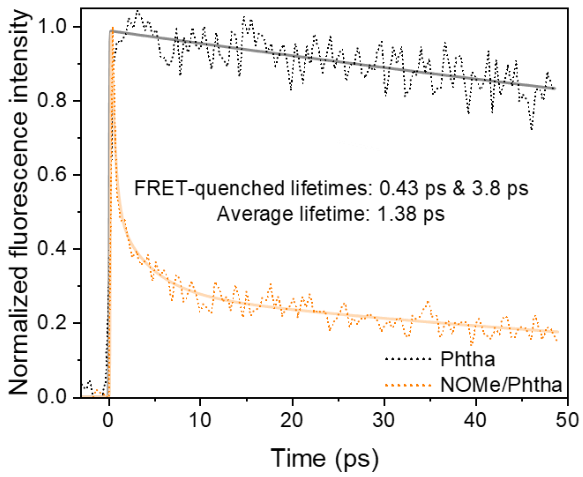

Figure S37. Comparison between the fluorescence decays (dotted lines, excitation at 400 nm and detection at 500 nm) in the ps regime of model **Phtha** (black) and triad **NOMe/Phtha** (orange) in air-equilibrated toluene solution. Solid lines represent the fittings of the respective decays.

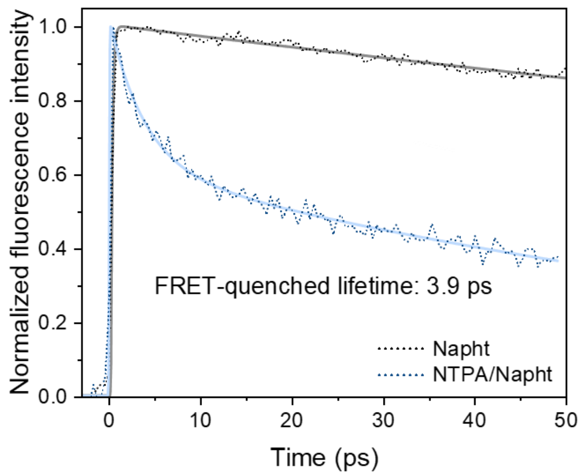

Figure S38. Comparison between the fluorescence decays (dotted lines, excitation at 400 nm and detection at 500 nm) in the ps regime of model **Napht** (black) and triad **NTPA/Napht** (blue) in air-equilibrated toluene solution. Solid lines represent the fittings of the respective decays.

- ## Background emission determination

Table S8. Background emission of all compounds under study.

Compound	Background emission[a]
**DASA/FRT**	< 0.12
**DASA/Napht**	< 0.14
**NOMe/Napht**	< 0.04
***asy*NOMe/Napht**	< 0.03
**NOMe/Phtha**	< 0.03
**NTPA/Napht**	< 0.01

[a] The background emission is determined as the ratio between the fluorescence intensities of the PSC-2PAP dyad/triad and the corresponding 2PAP monomer. As the excitation light for emission readout triggers isomerization to the fluorescent form, we have given the background emission as an upper limit.

- ## Time-dependent fluorescence detection under 2P-excitation and corresponding log-log plots

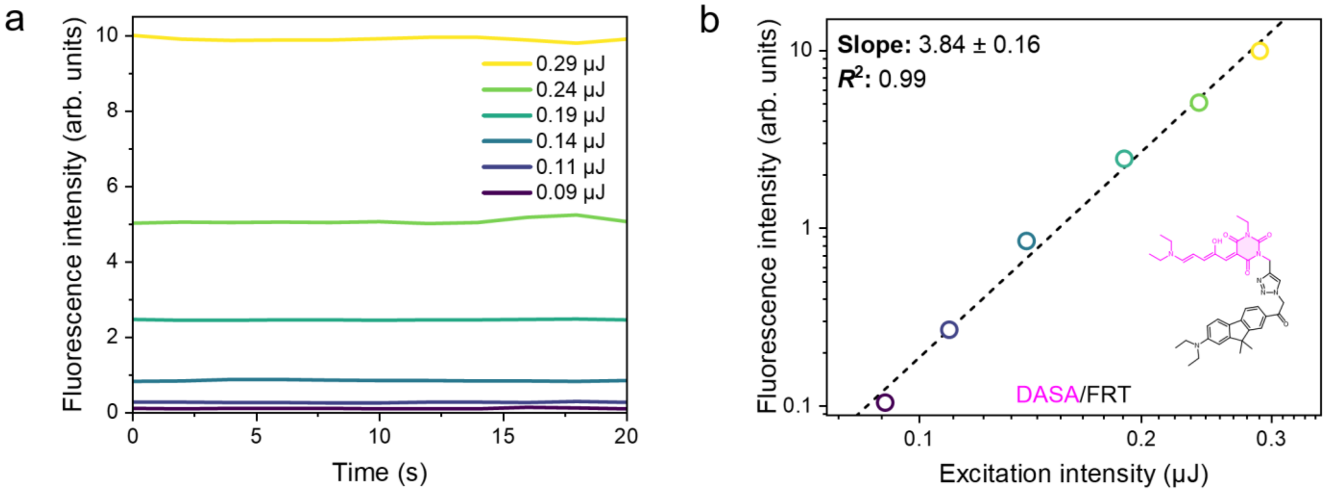

Figure S39. Validation experiments for **DASA/FRT** under two-photon excitation. (a) Fluorescence intensities of **DASA/FRT** in air-equilibrated toluene, monitored for 20 s at equilibrium upon excitation at 800 nm at varying excitation intensities. (b) Corresponding double logarithmic plot.

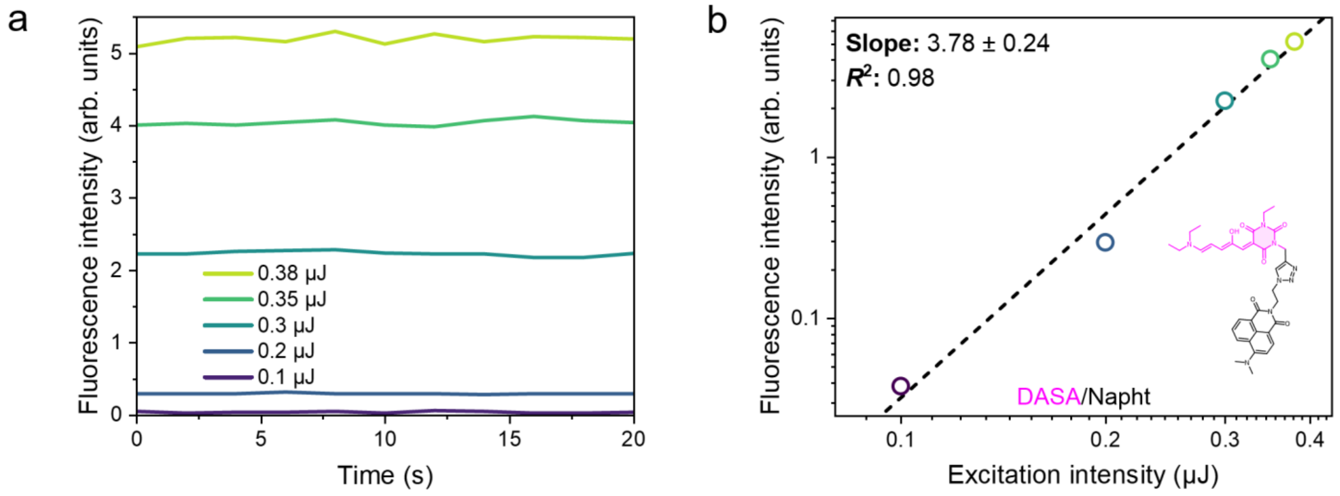

Figure S40. Validation experiments for **DASA/Napht** under two-photon excitation. (a) Fluorescence intensities of **DASA/Napht** in air-equilibrated toluene, monitored for 20 s at equilibrium upon excitation at 800 nm at varying excitation intensities. (b) Corresponding double logarithmic plot.

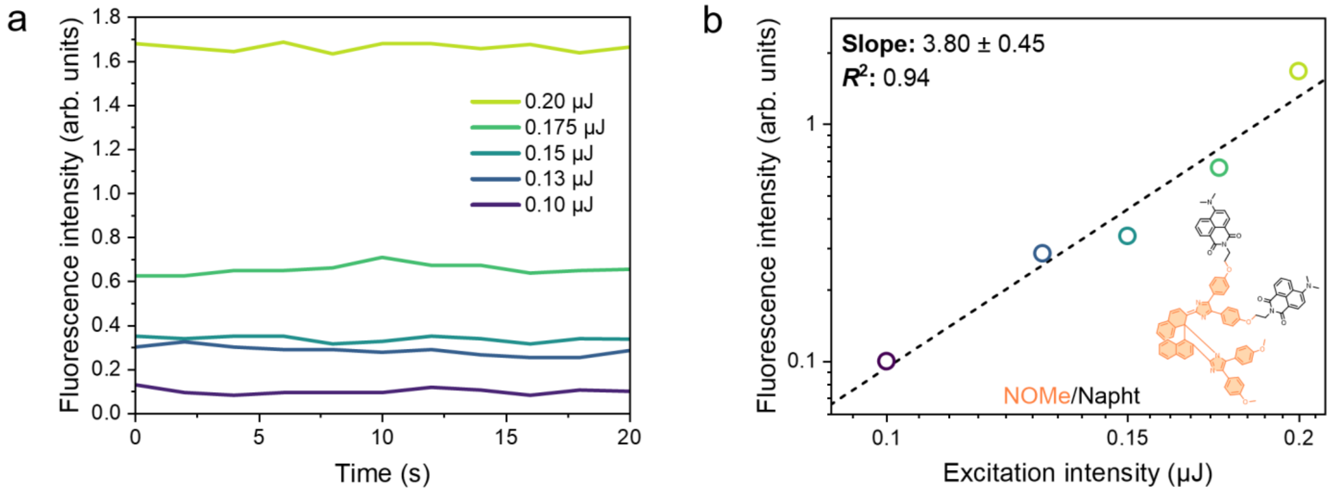

Figure S41. Validation experiments for **NOMe/Napht** under two-photon excitation. (a) Fluorescence intensities of **NOMe/Napht** in air-equilibrated toluene, monitored for 20 s at equilibrium upon excitation at 800 nm at varying excitation intensities. (b) Corresponding double logarithmic plot.

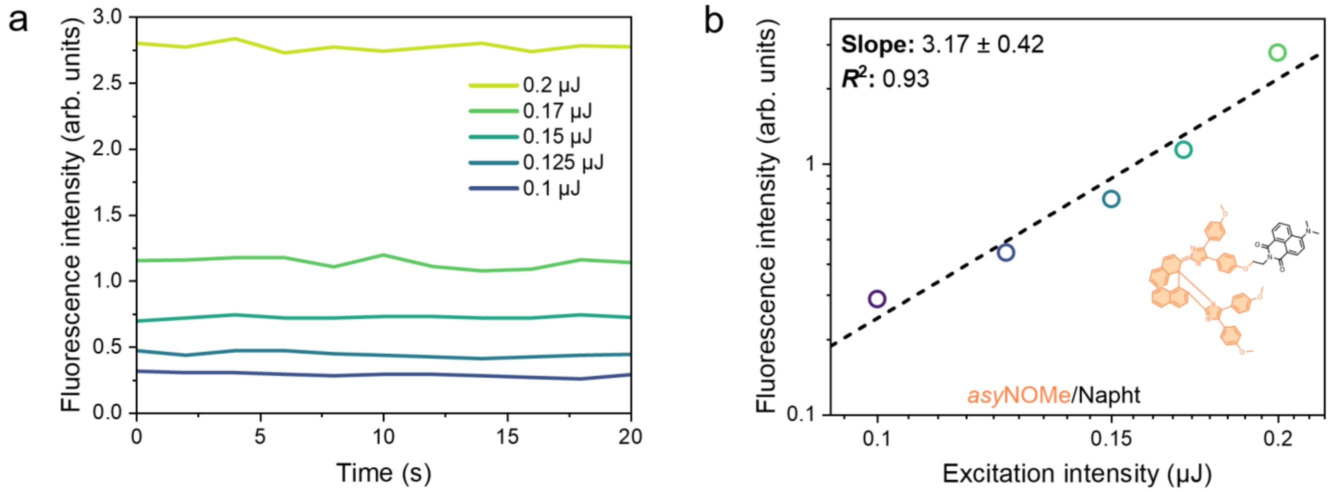

Figure S42. Validation experiments for ***asy*NOMe/Napht** under two-photon excitation. (a) Fluorescence intensities of ***asy*NOMe/Napht** in air-equilibrated toluene, monitored for 20 s at equilibrium upon excitation at 800 nm at varying excitation intensities. (b) Corresponding double logarithmic plot.

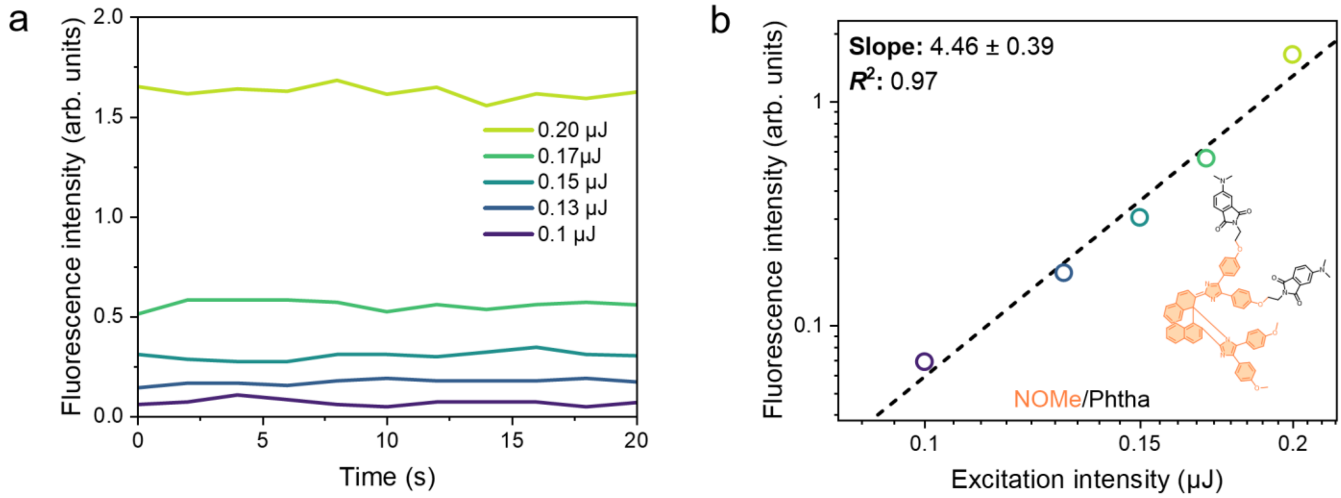

Figure S43. Validation experiments for **NOMe/Phtha** under two-photon excitation. (a) Fluorescence intensities of **NOMe/Phtha** in air-equilibrated toluene, monitored for 20 s at equilibrium upon excitation at 800 nm at varying excitation intensities. (b) Corresponding double logarithmic plot.

5. NMR spectra

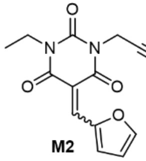
M2

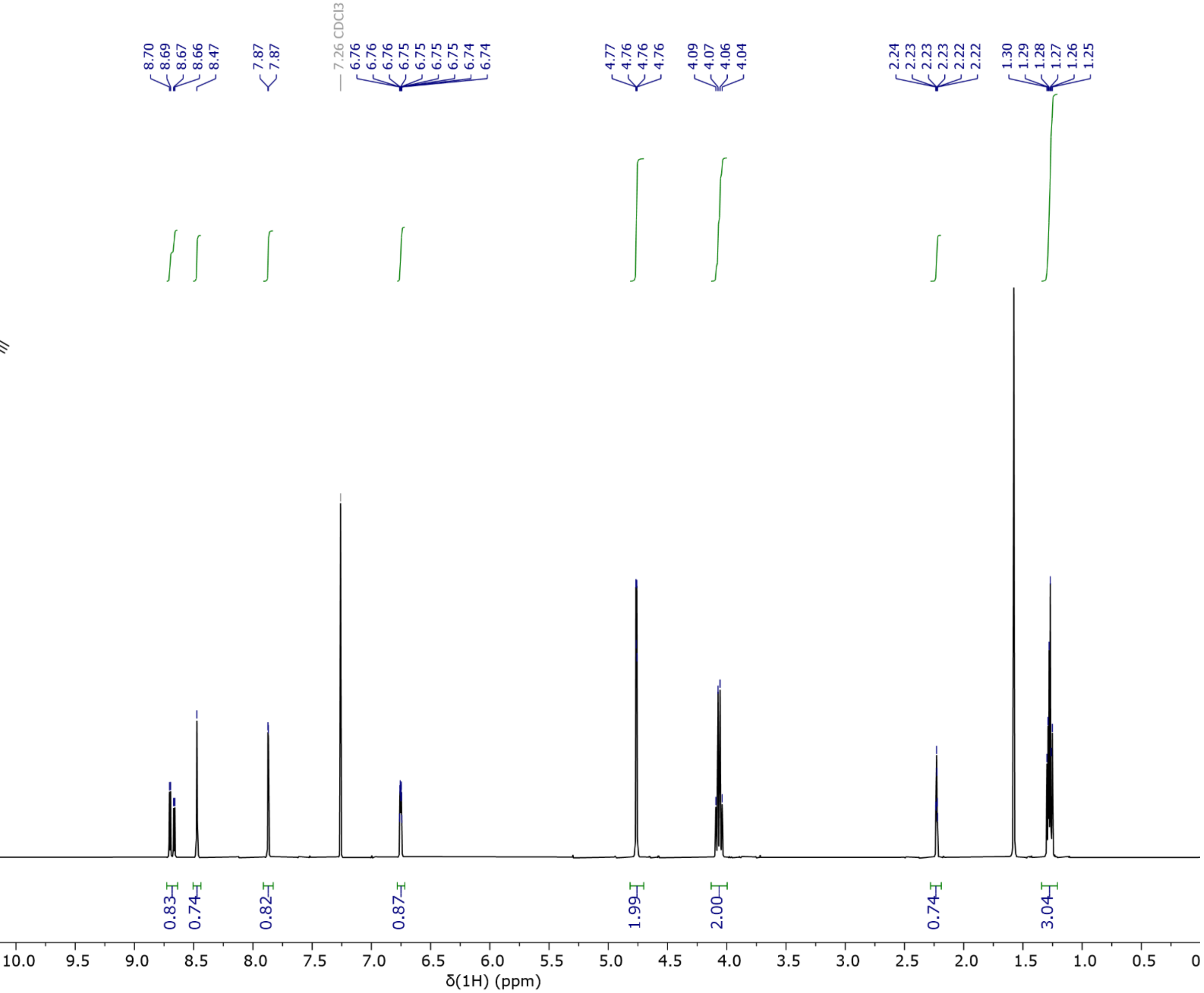

Figure S44. ^{1}H NMR spectrum (400 MHz, $CDCl_3$) of **M2**.

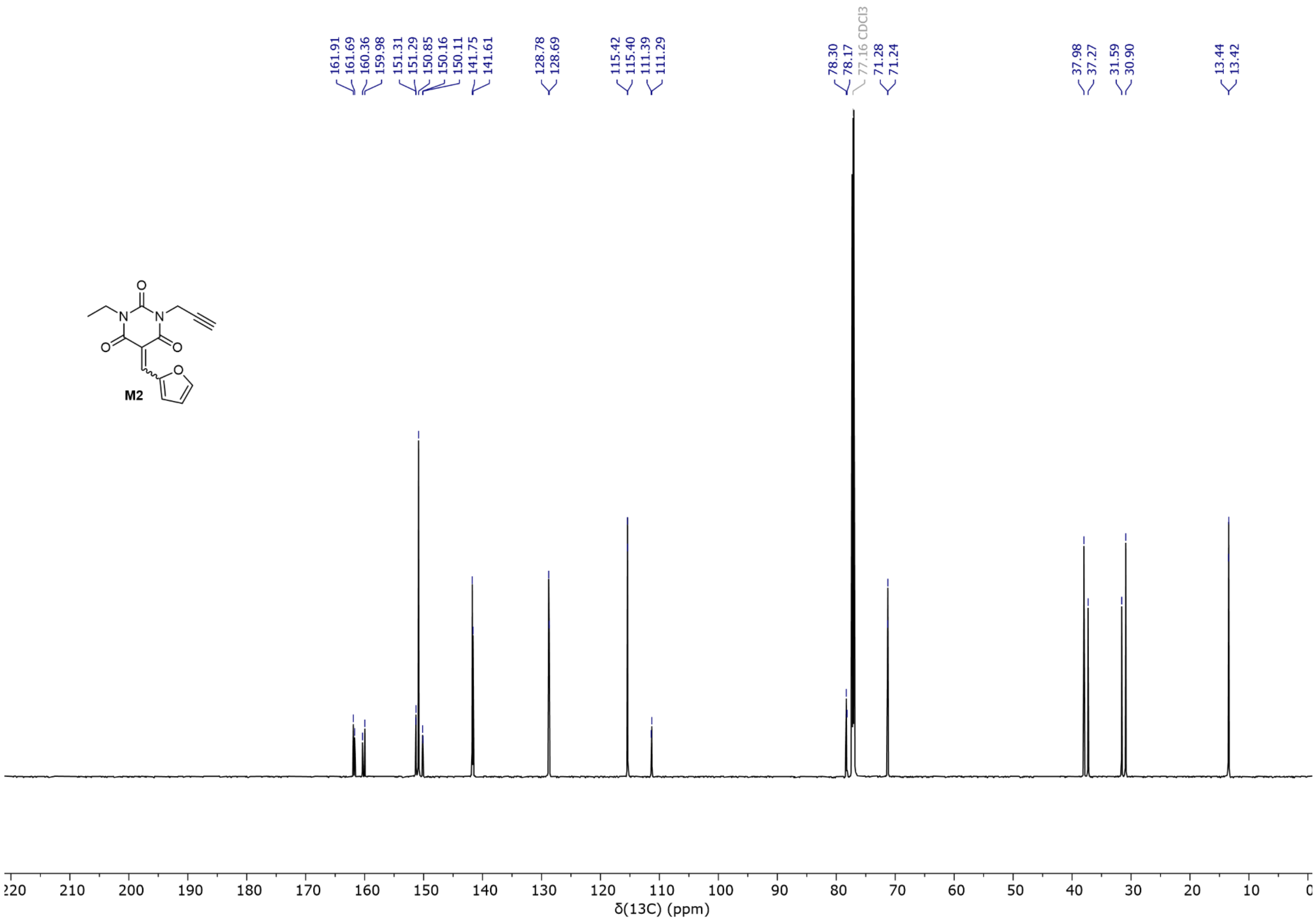

Figure S45. ^{13}C NMR spectrum (201 MHz, $CDCl_3$) of **M2**.

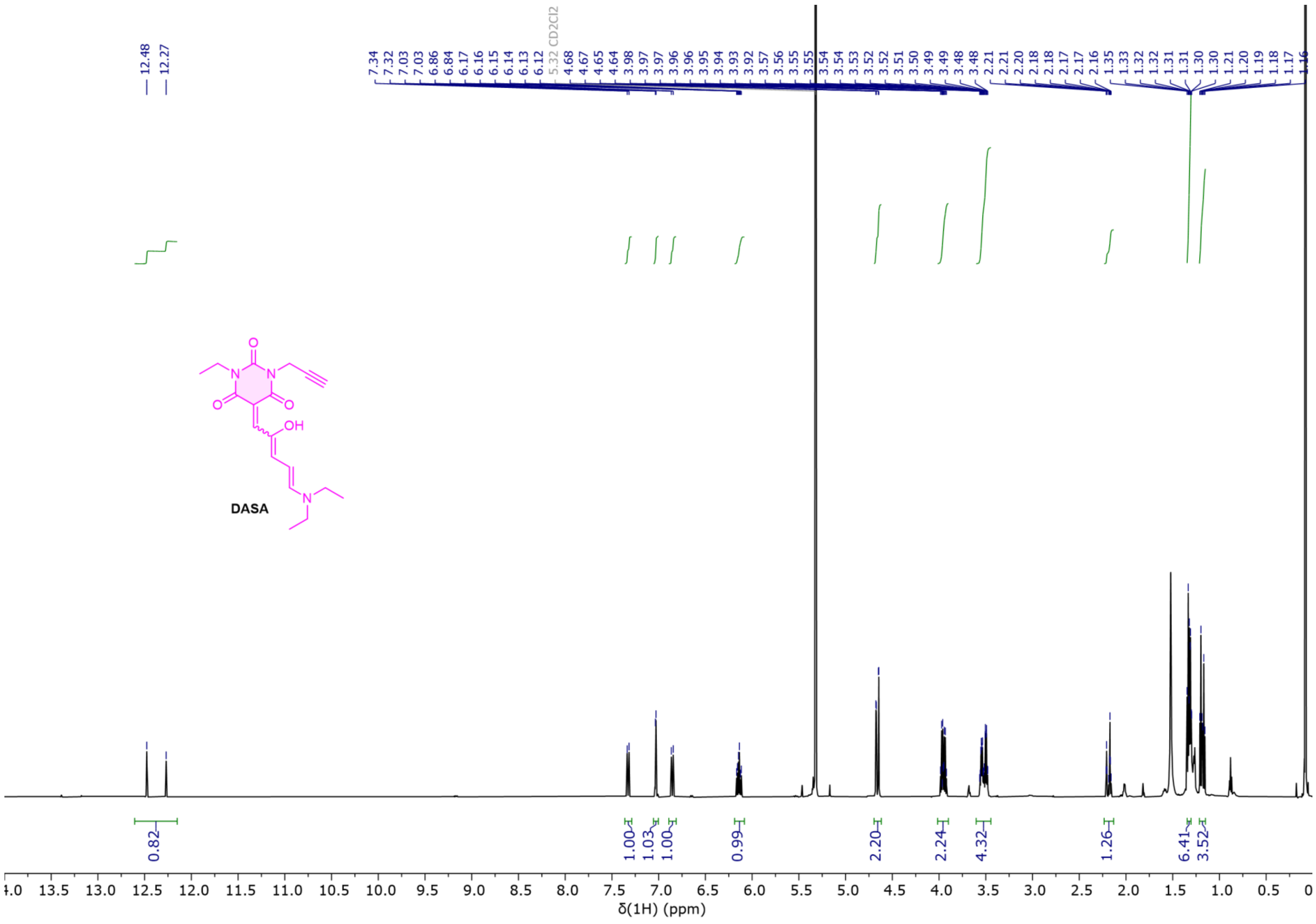

Figure S46. ^{1}H NMR spectrum (600 MHz, CD_2Cl_2) of **DASA**.

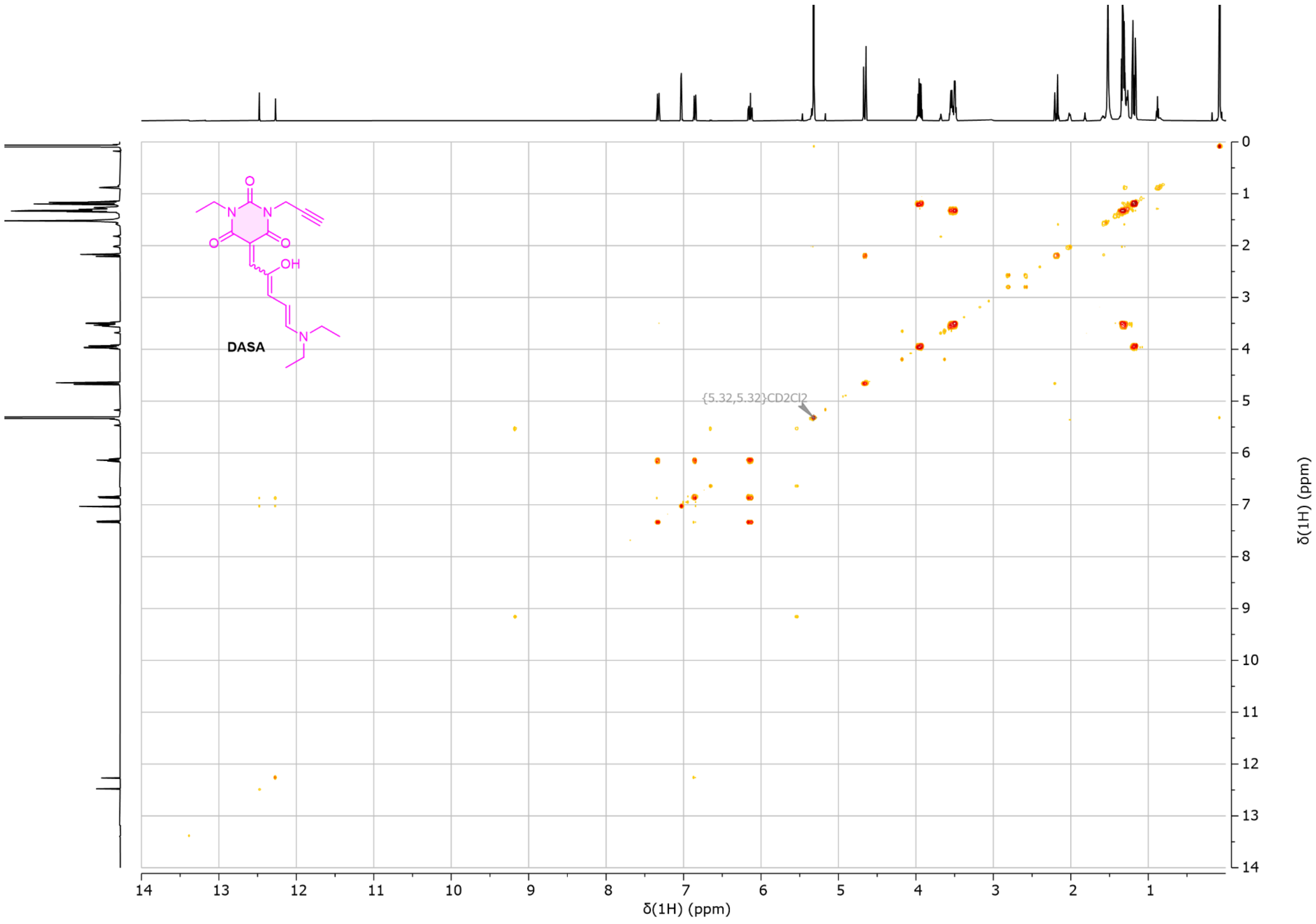

Figure S47. ^1H-^1H COSY spectrum (600 MHz, CD_2Cl_2) of **DASA**.

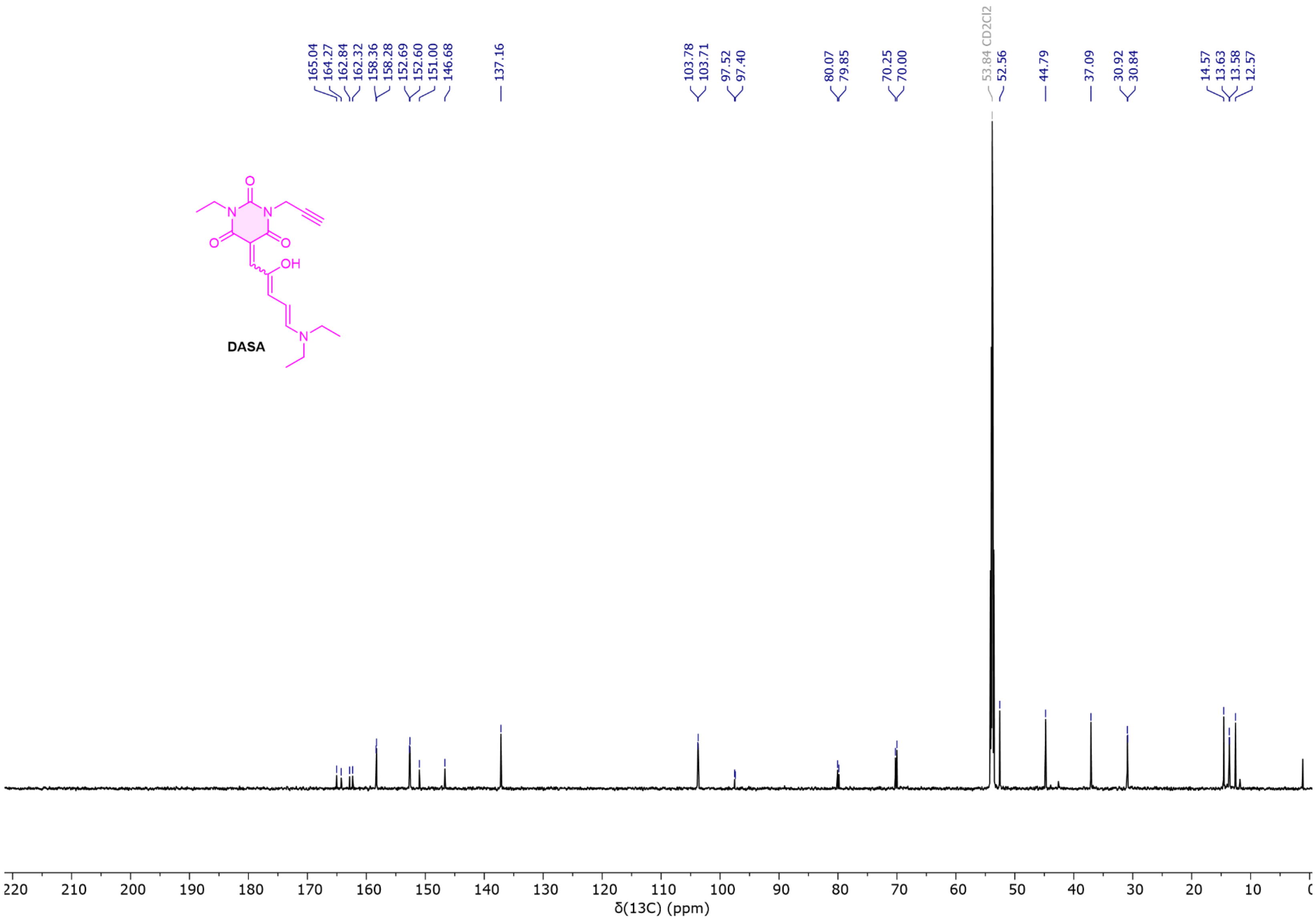

Figure S48. ^{13}C NMR spectrum (201 MHz, CD_2Cl_2) of **DASA**.

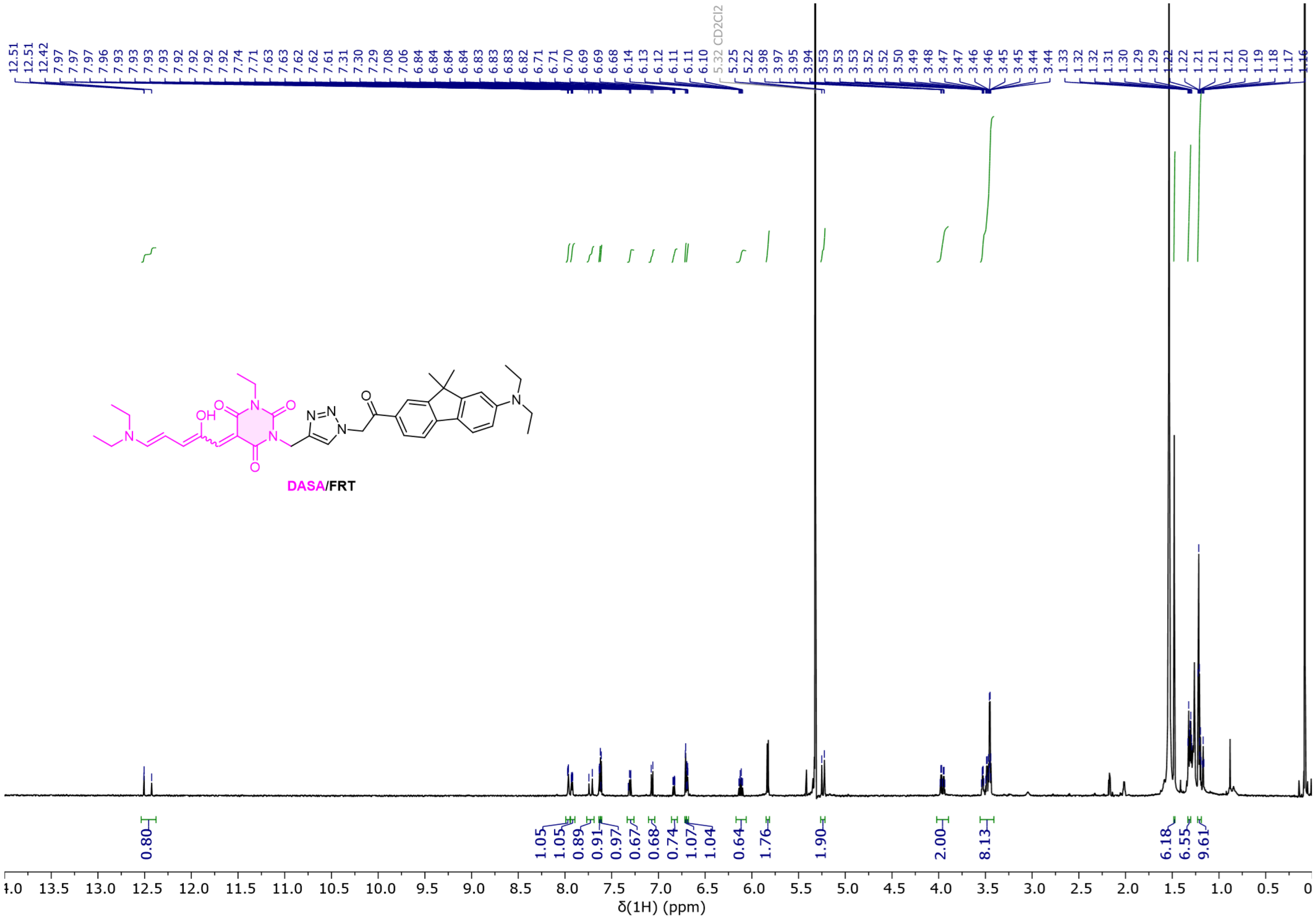

Figure S49. ^{1}H NMR spectrum (900 MHz, CD_2Cl_2) of **DASA/FRT**.

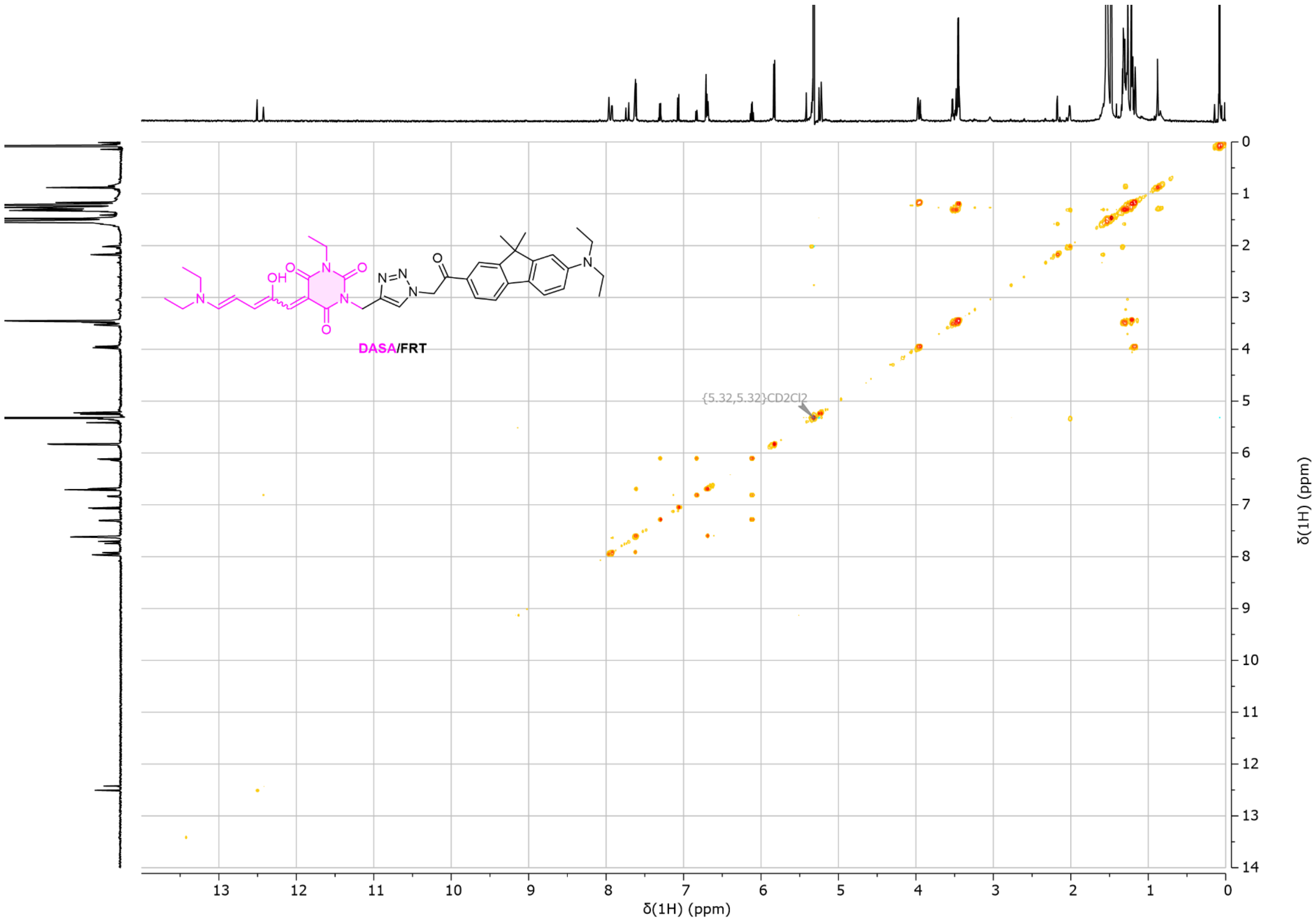

Figure S50. ^{1}H-^{1}H COSY spectrum (900 MHz, CD_2Cl_2) of **DASA/FRT**.

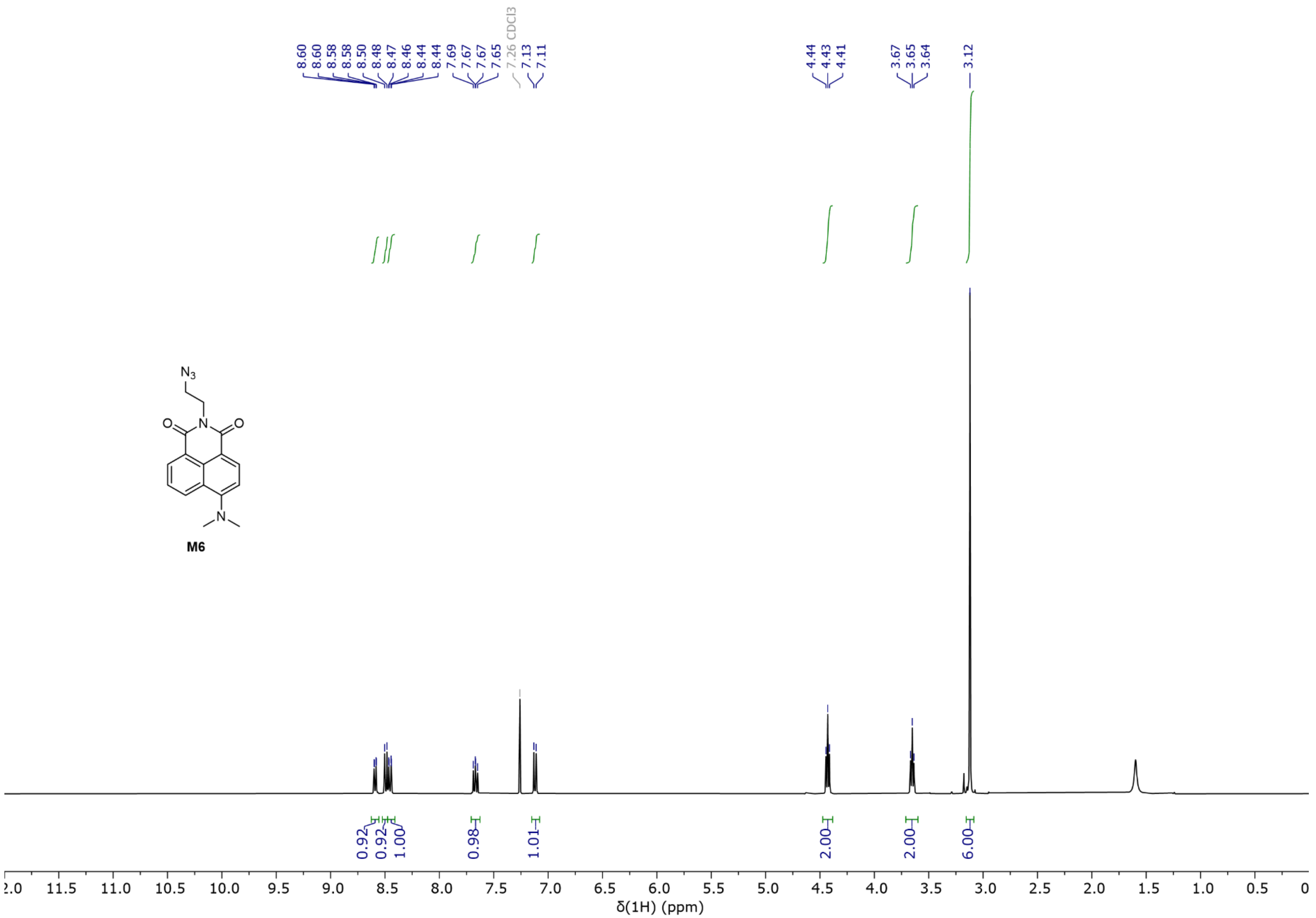

Figure S51. ^{1}H NMR spectrum (400 MHz, $CDCl_3$) of **M6**.

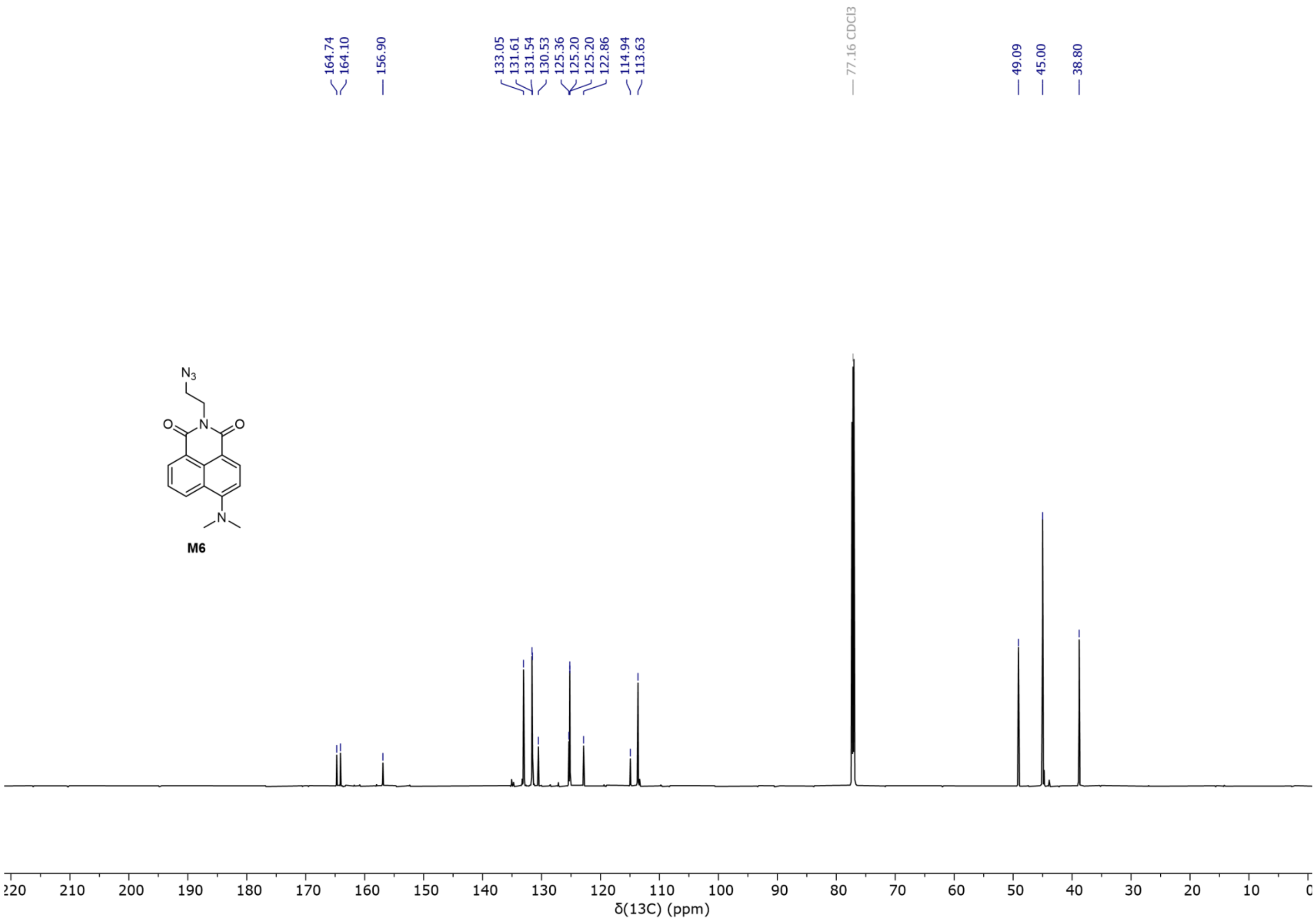

Figure S52. ^{13}C NMR spectrum (201 MHz, $CDCl_3$) of **M6**.

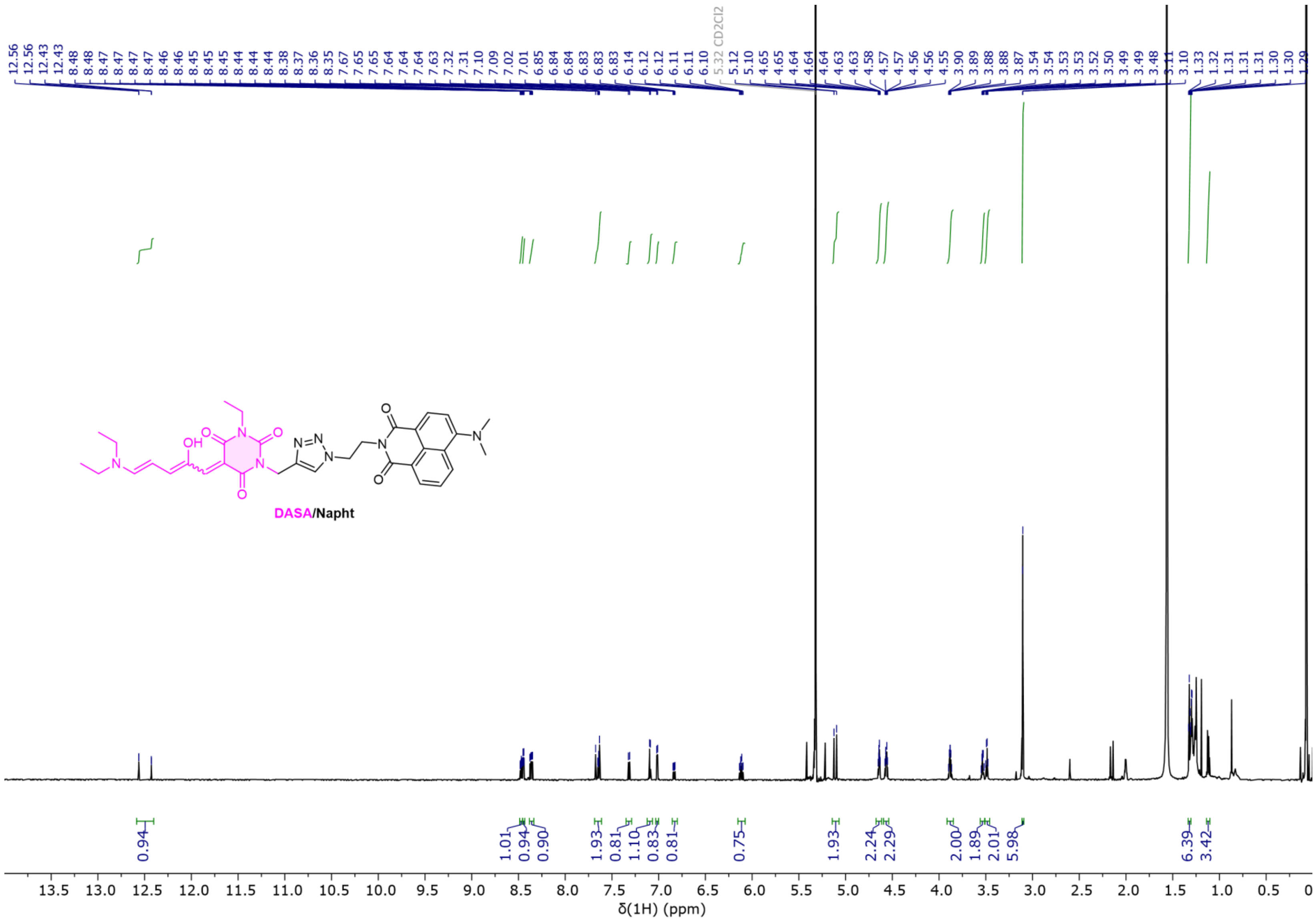

Figure S53. ^{1}H NMR spectrum (900 MHz, CD_2Cl_2) of **DASA/Napht**.

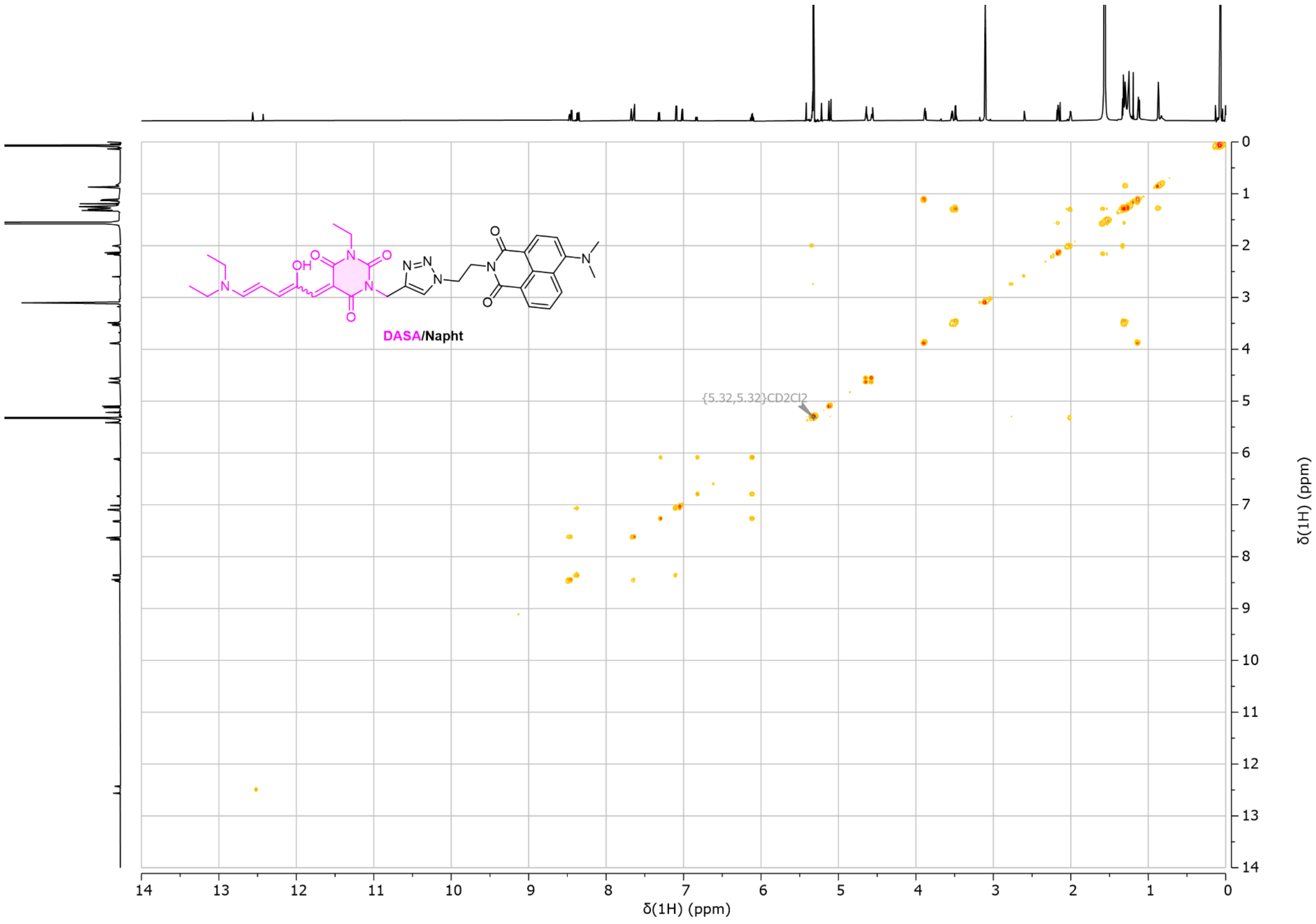

Figure S54. ^{1}H-^{1}H COSY spectrum (900 MHz, CD_2Cl_2) of **DASA/Napht**.

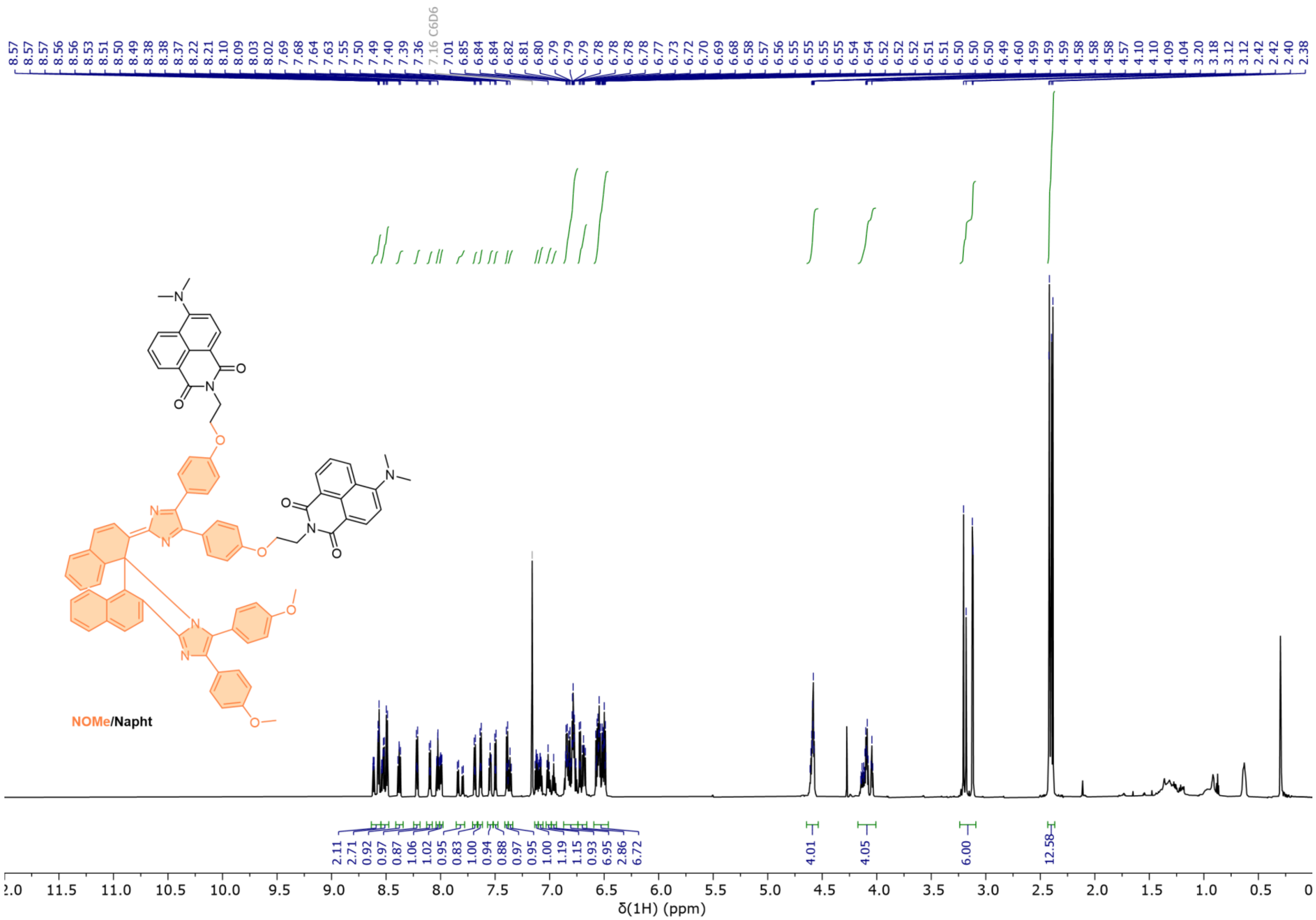

Figure S55. ^{1}H NMR spectrum (800 MHz, C_6D_6) of **NOMe/Napht**.

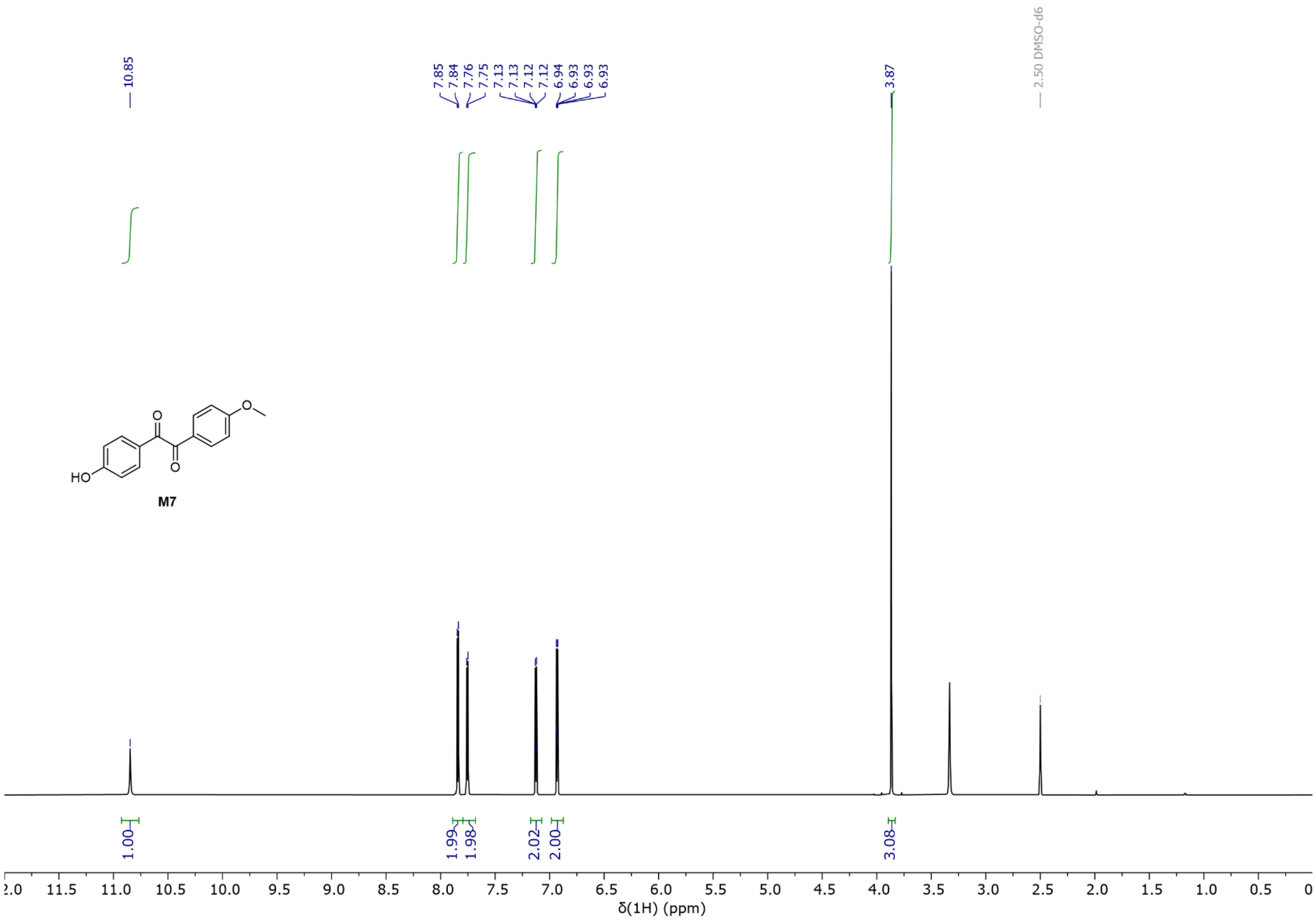

Figure S56. ^{1}H NMR spectrum (800 MHz, DMSO-d_6) of **M7**.

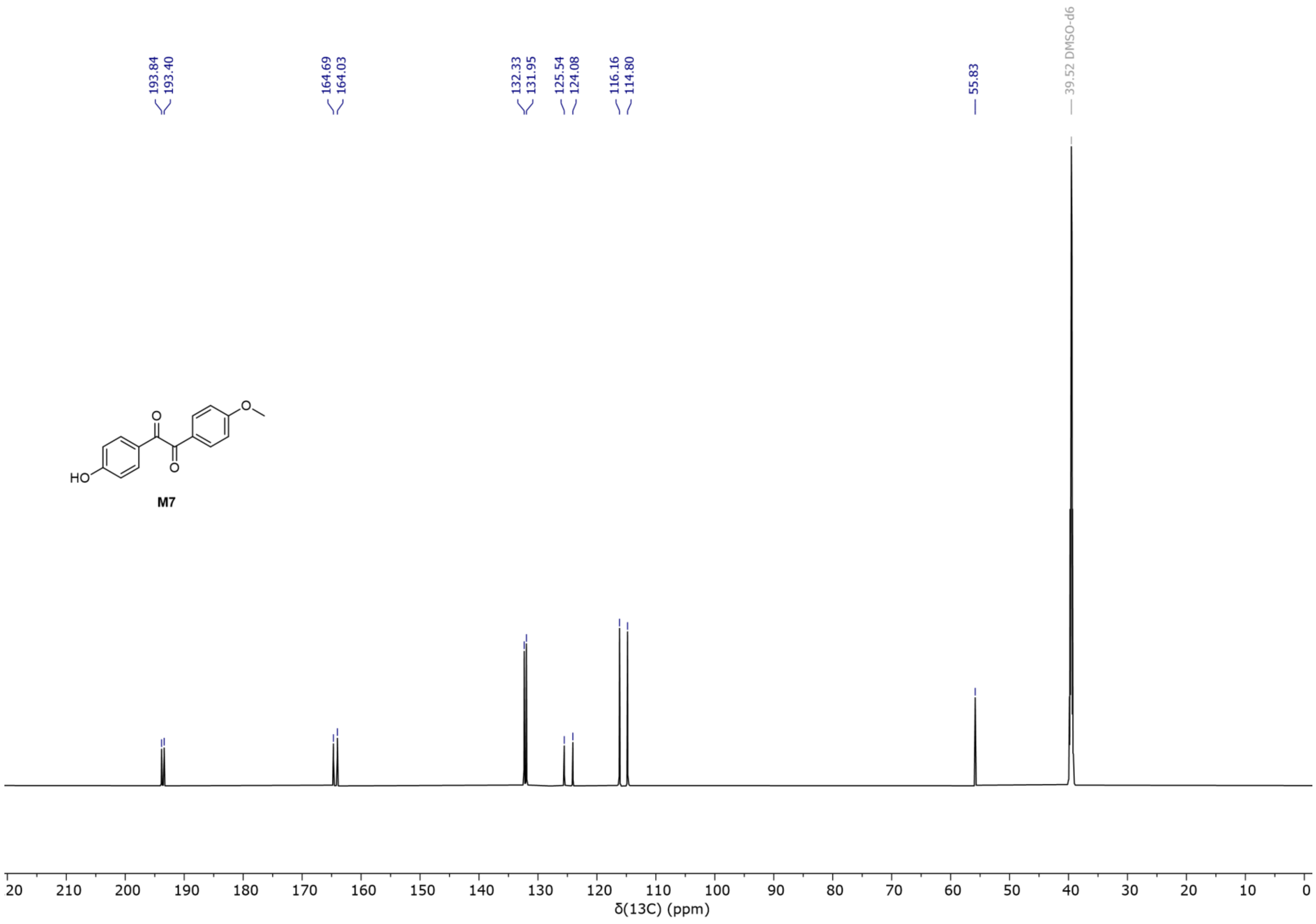

Figure S57. ^{13}C NMR spectrum (201 MHz, DMSO-d_6) of **M7**.

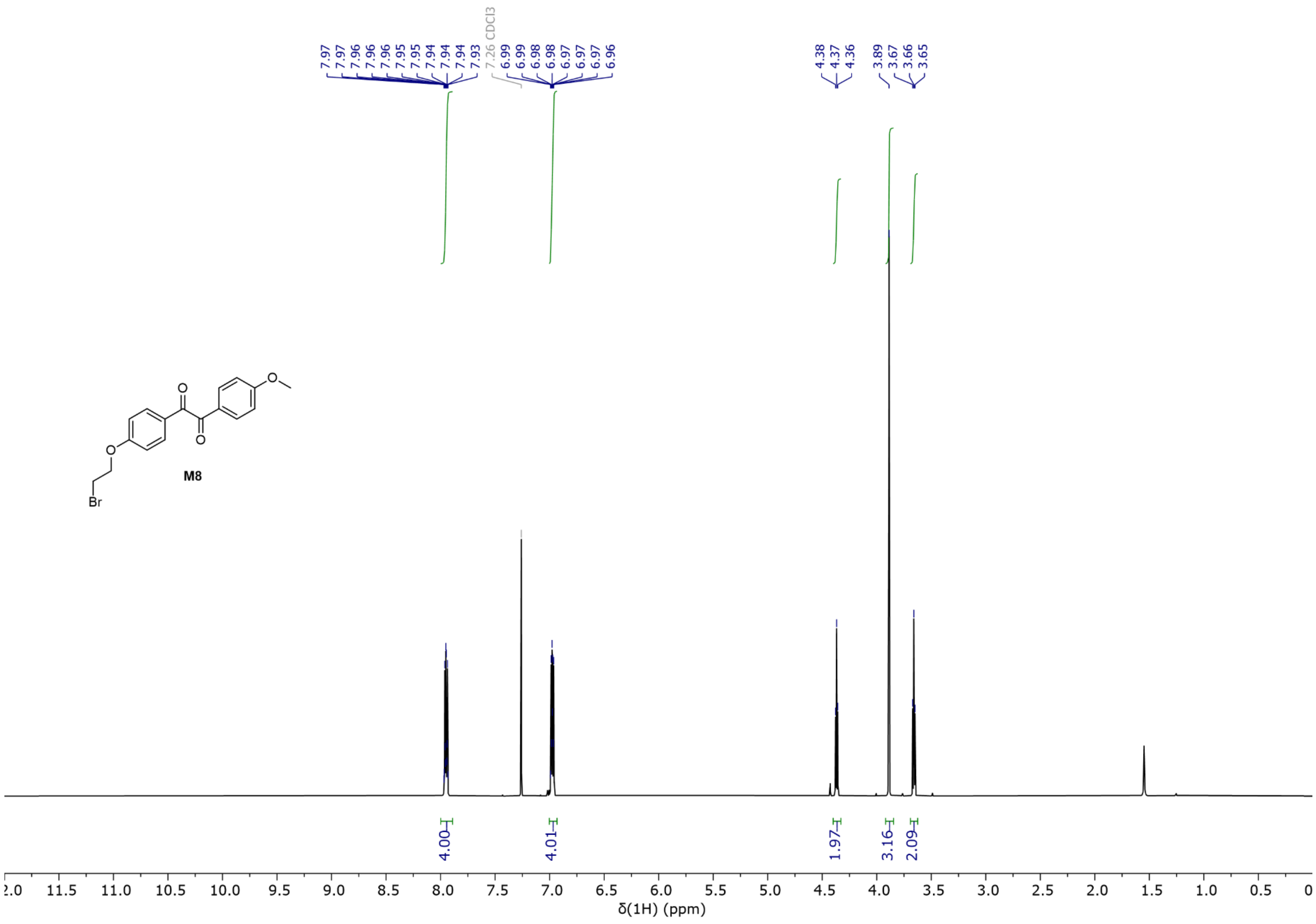

Figure S58. ^{1}H NMR spectrum (600 MHz, $CDCl_3$) of **M8**.

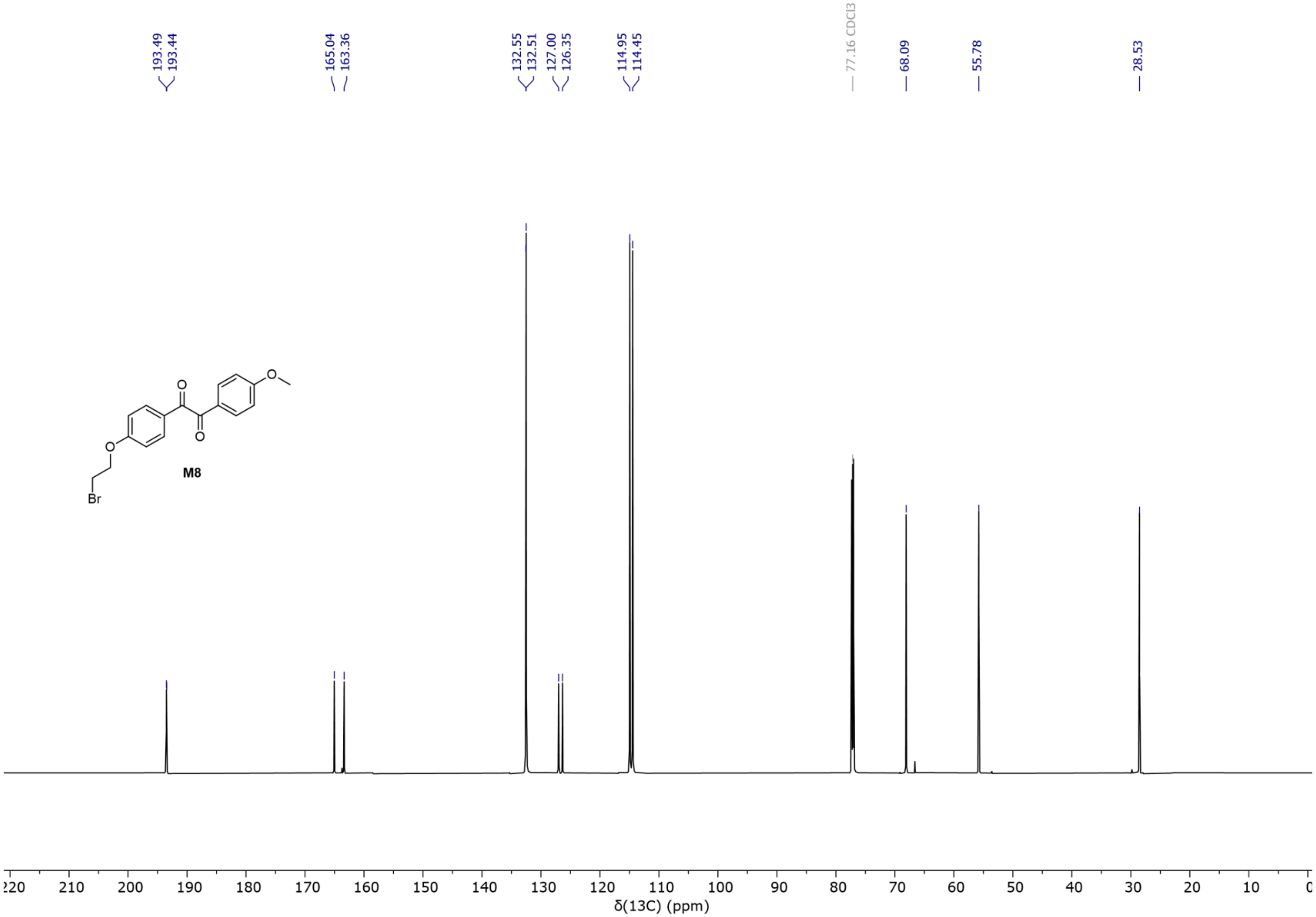

Figure S59. ^{13}C NMR spectrum (201 MHz, $CDCl_3$) of **M8**.

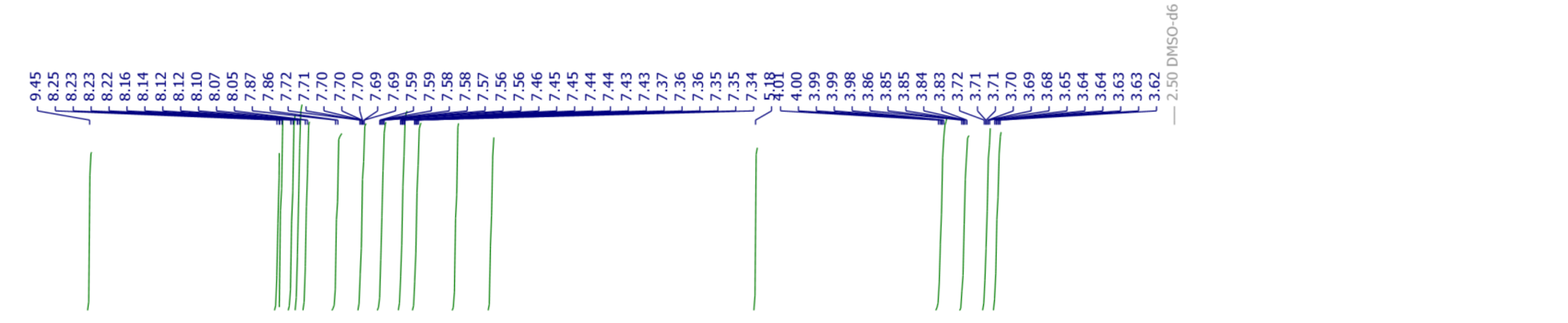

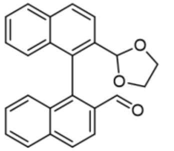

M10

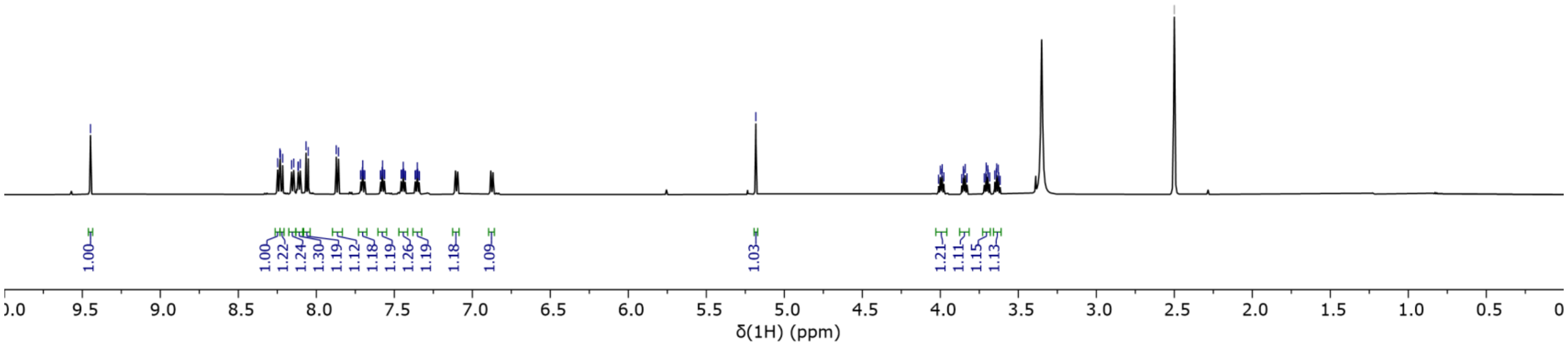

Figure S60. ^{1}H NMR spectrum (600 MHz, DMSO-d_6) of **M10**.

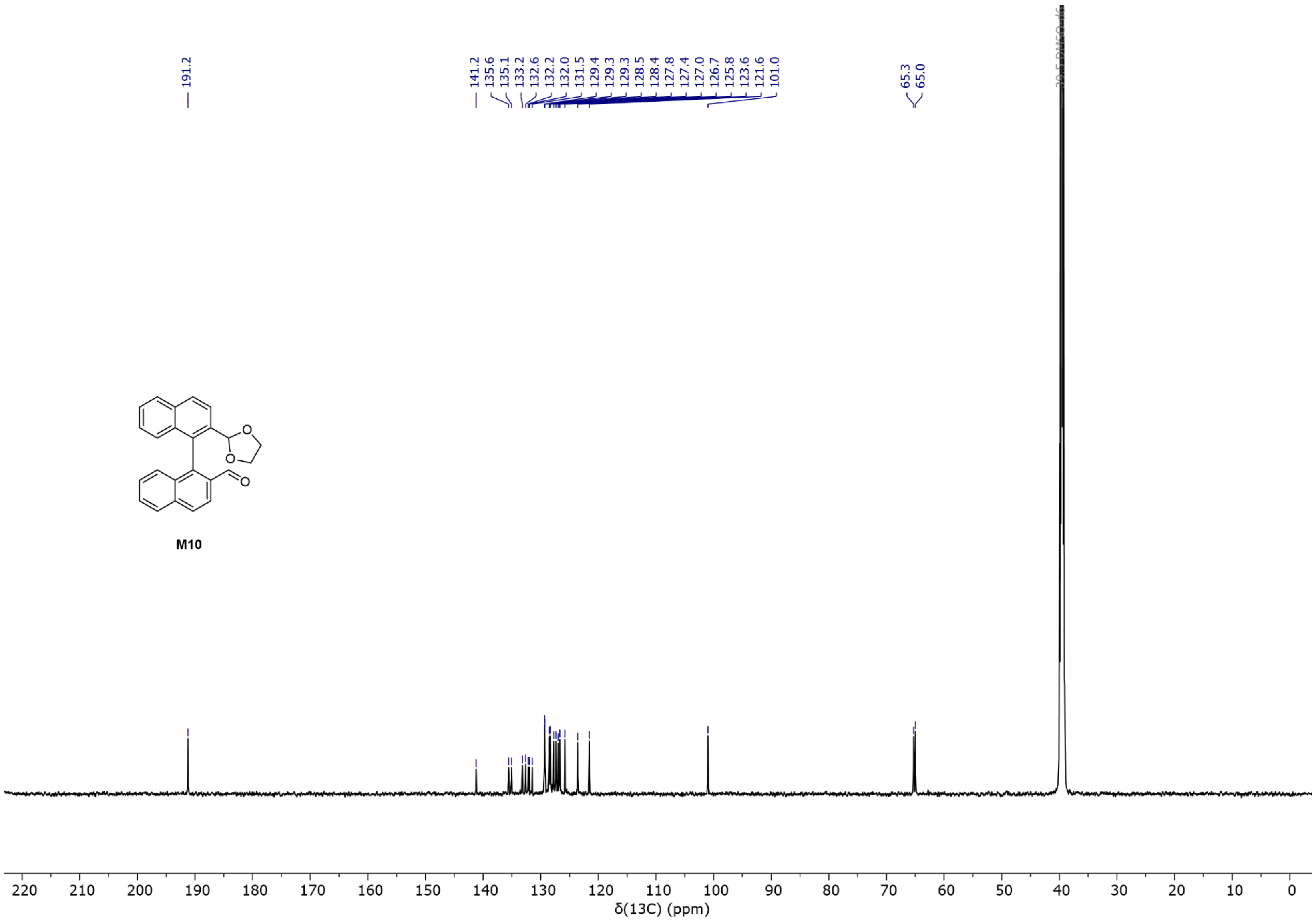

Figure S61. ^{13}C NMR spectrum (151 MHz, DMSO-d_6) of **M10**.

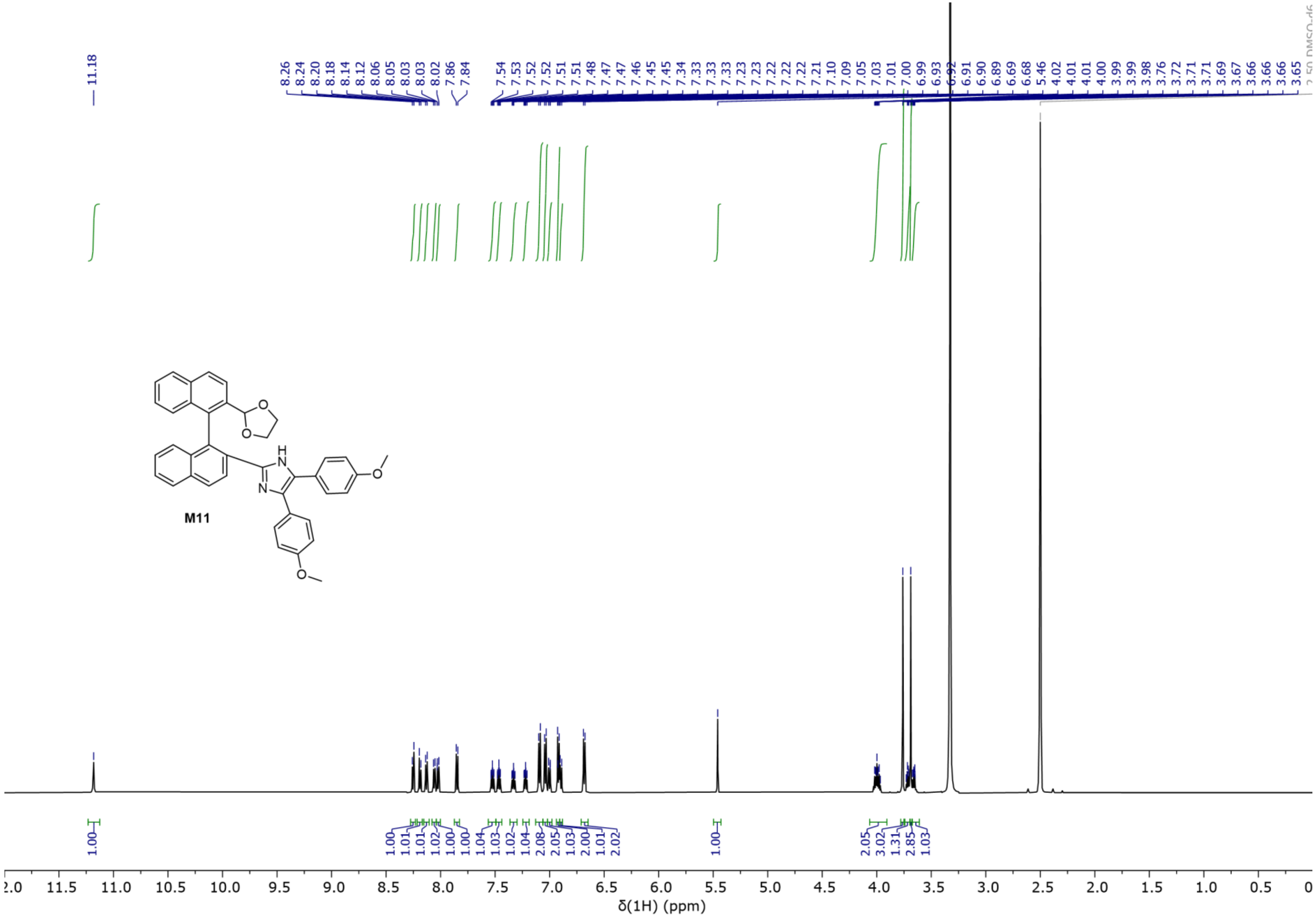

Figure S62. ^{1}H NMR spectrum (600 MHz, DMSO-d_6) of **M11**.

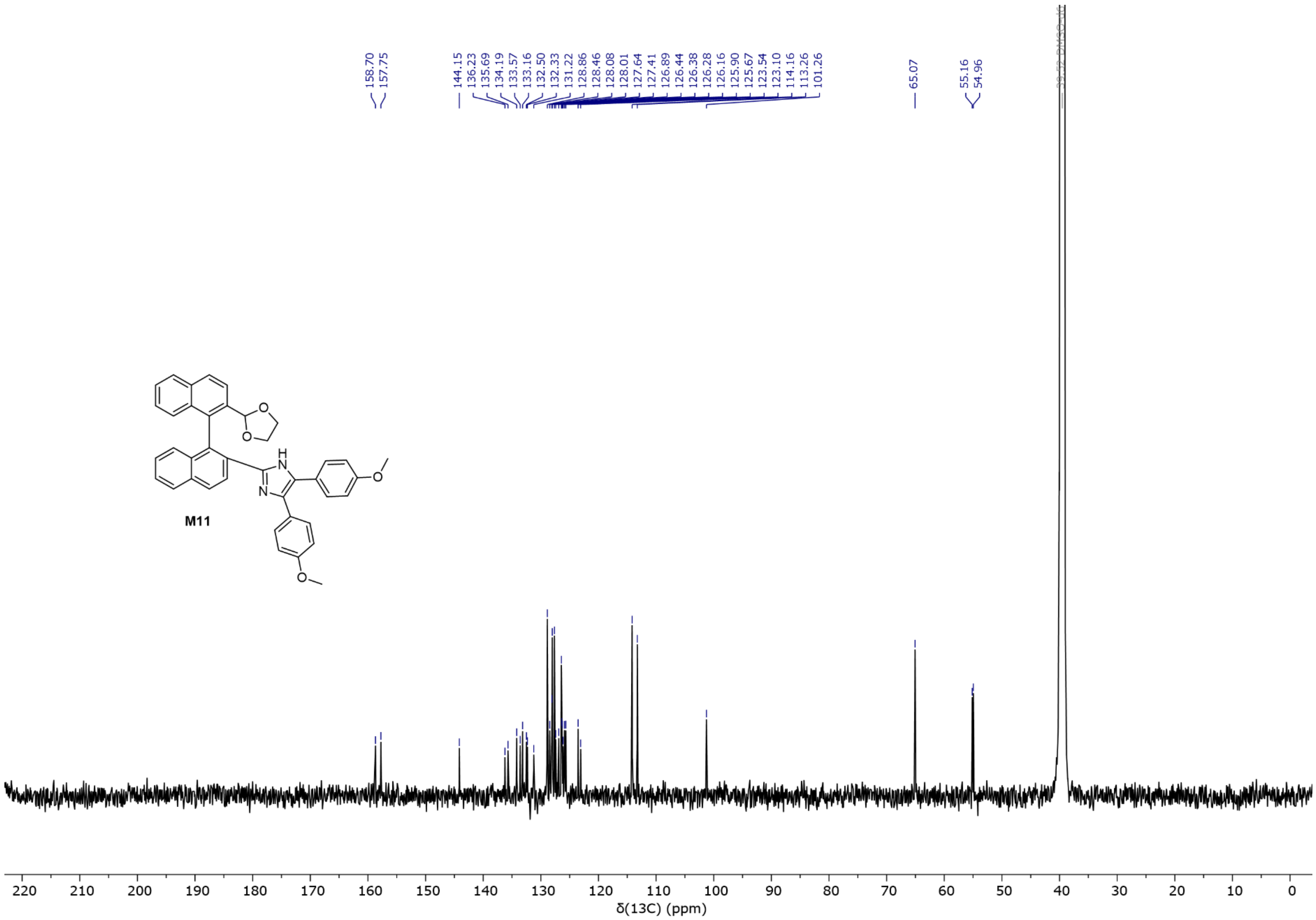

Figure S63. ^{13}C NMR spectrum (151 MHz, DMSO-d_6) of **M11**.

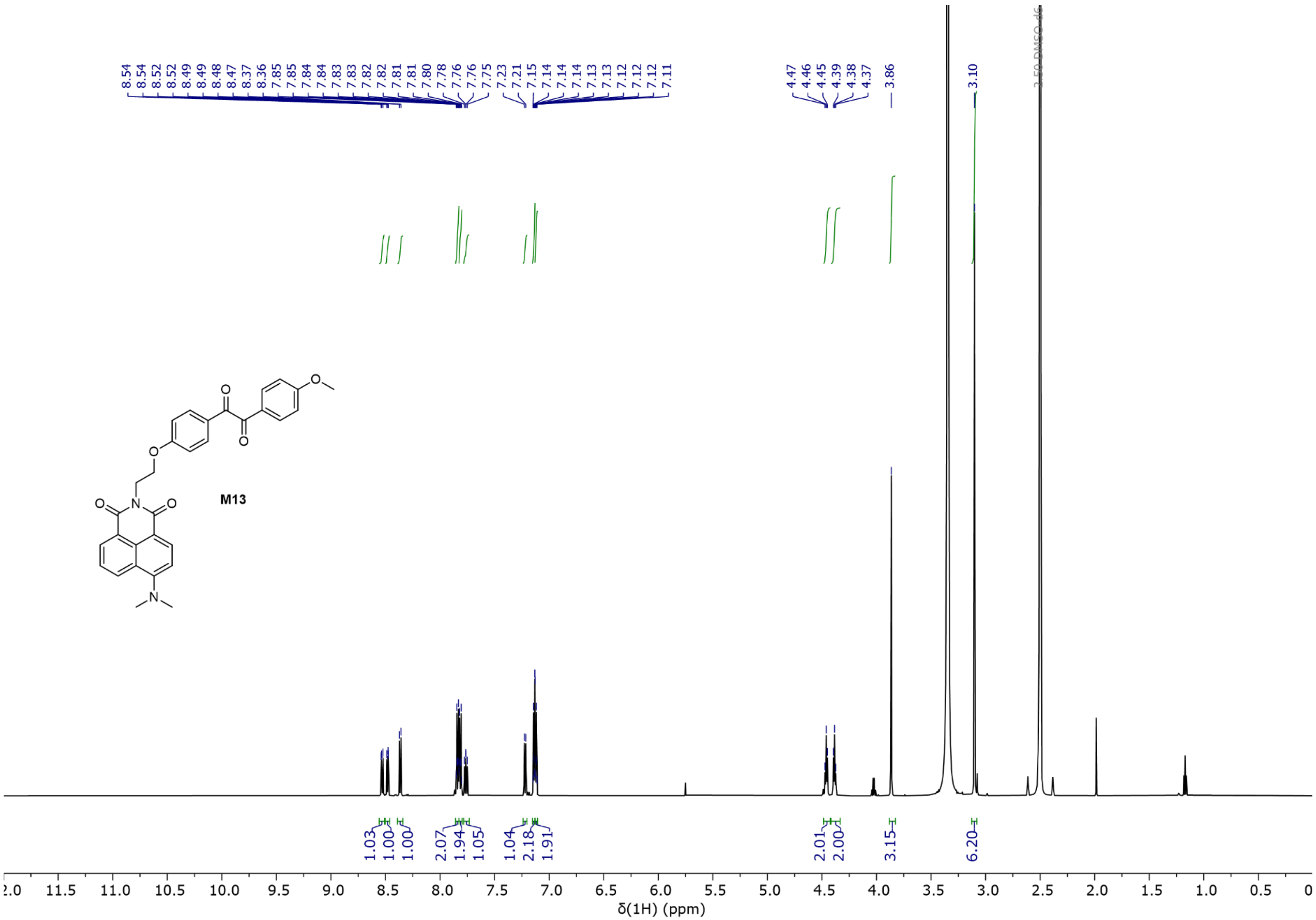

Figure S64. ^{1}H NMR spectrum (600 MHz, DMSO-d_6) of **M13**.

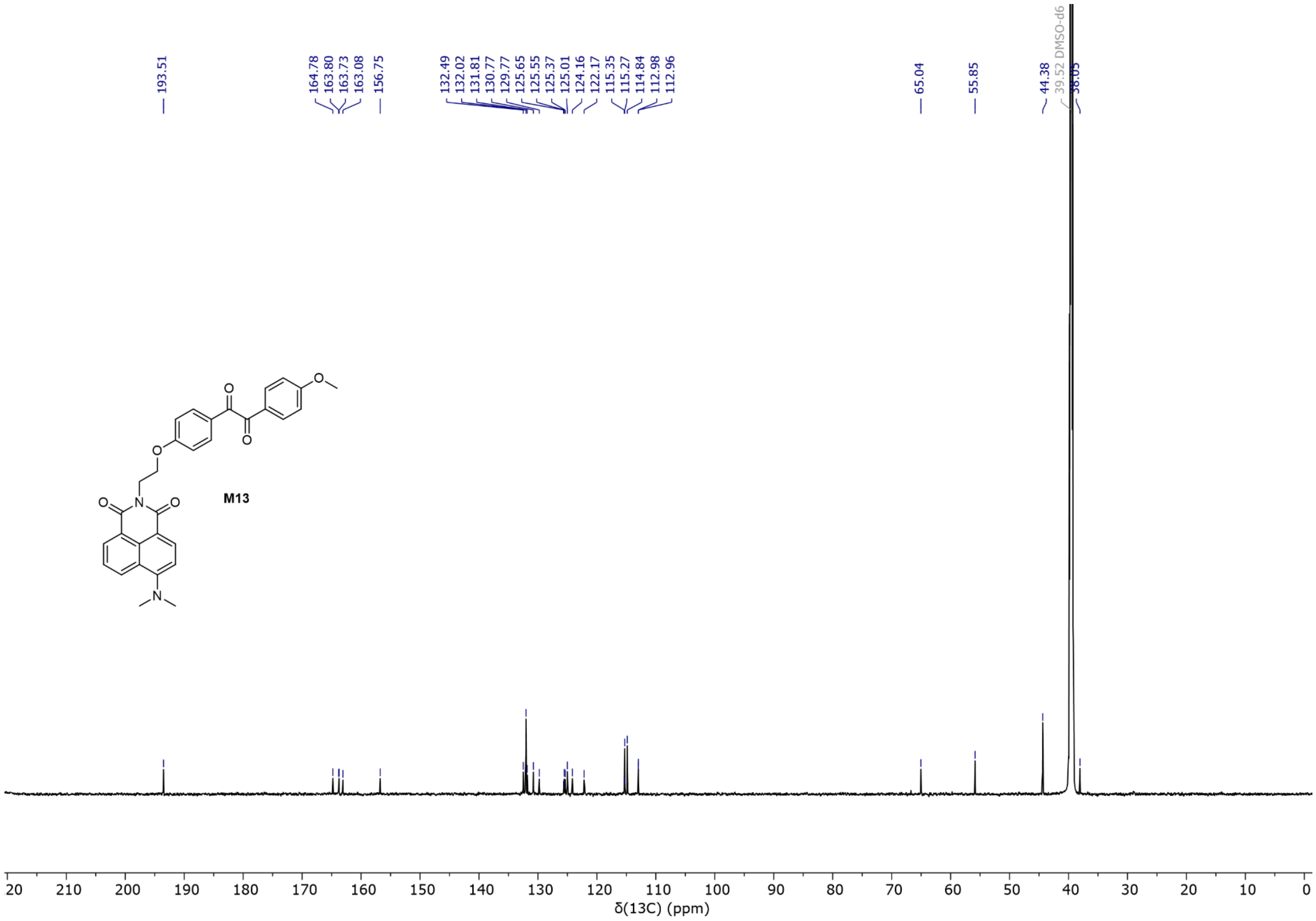

M13

Figure S65. ^{13}C NMR spectrum (151 MHz, DMSO-d_6) of **M13**.

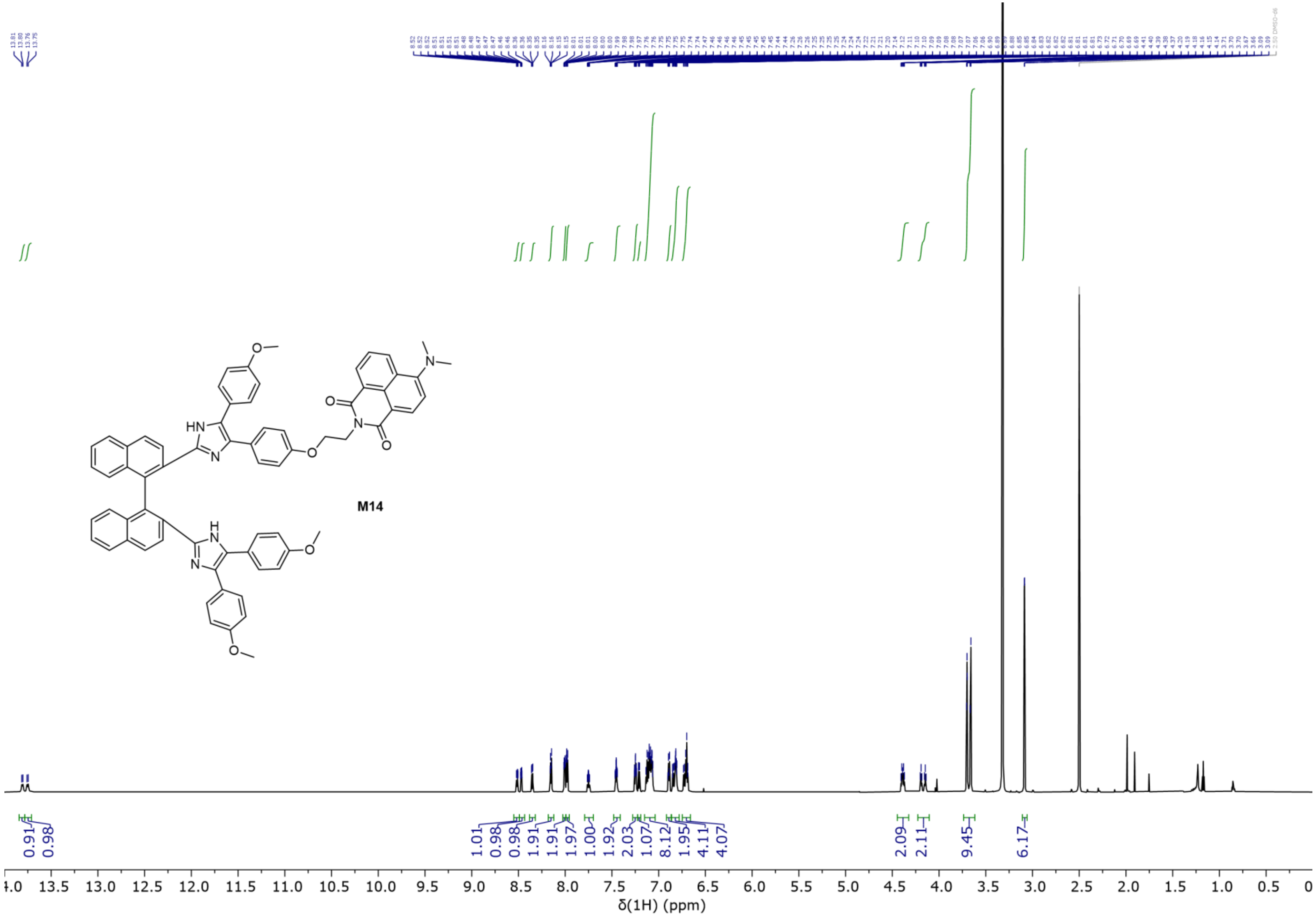

Figure S66. ^{1}H NMR spectrum (600 MHz, DMSO-d_6) of **M14**.

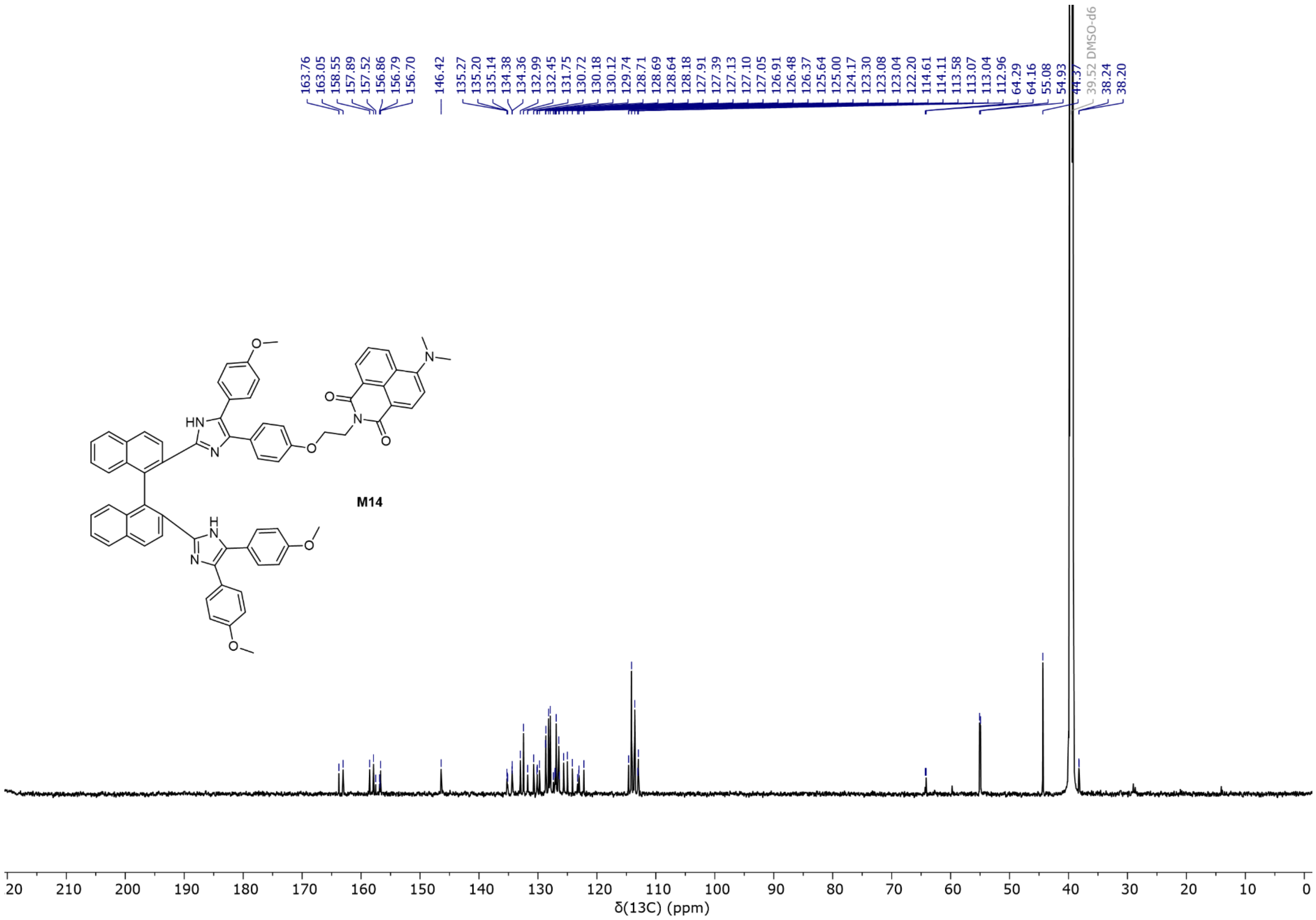

Figure S67. ^{13}C NMR spectrum (201 MHz, DMSO-d_6) of **M14**.

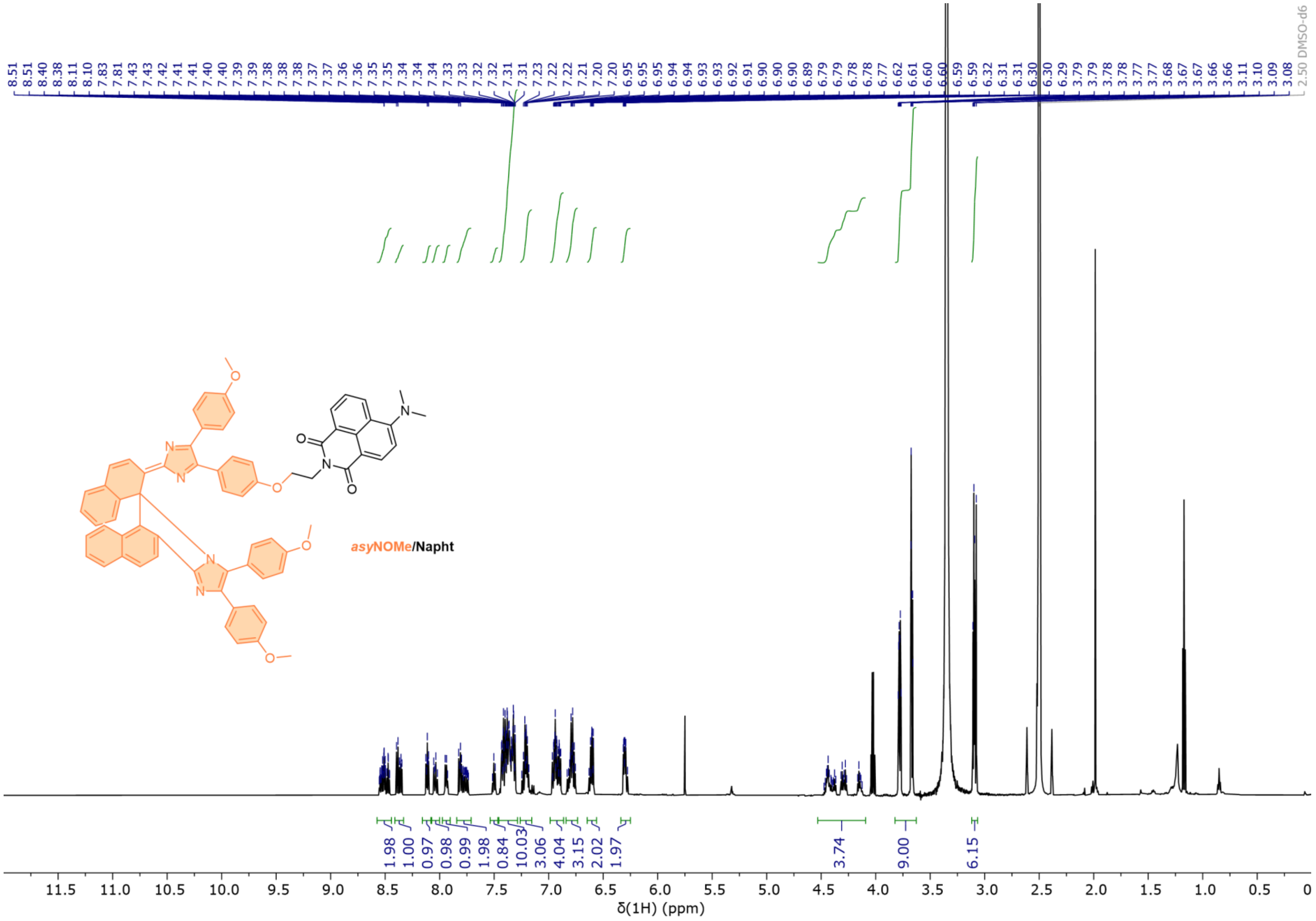

Figure S68. ^{1}H NMR spectrum (600 MHz, DMSO-d_6) of ***asy*NOMe/Napht**.

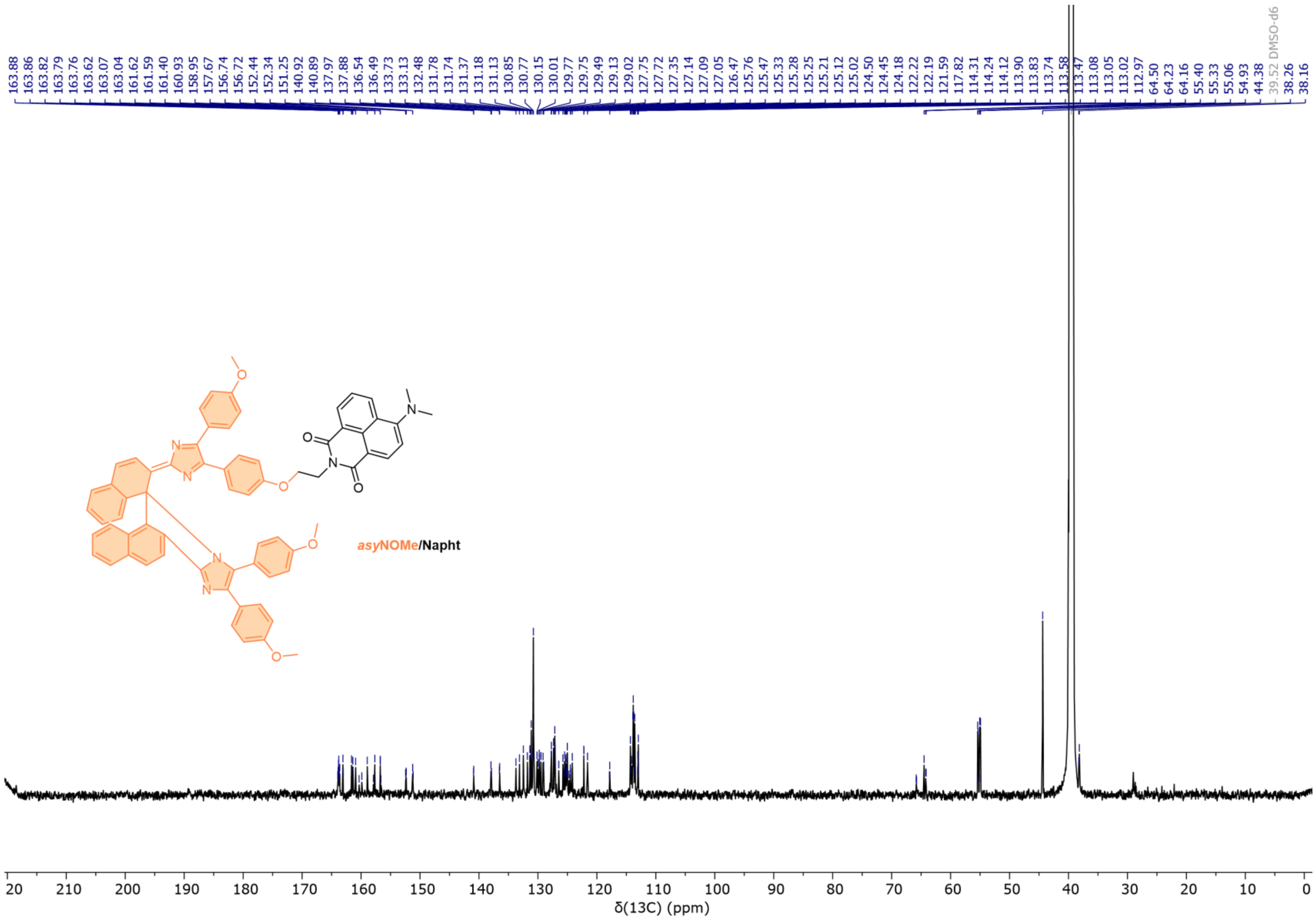

Figure S69. ^{13}C NMR spectrum (201 MHz, DMSO-d_6) of ***asy*NOMe/Napht**.

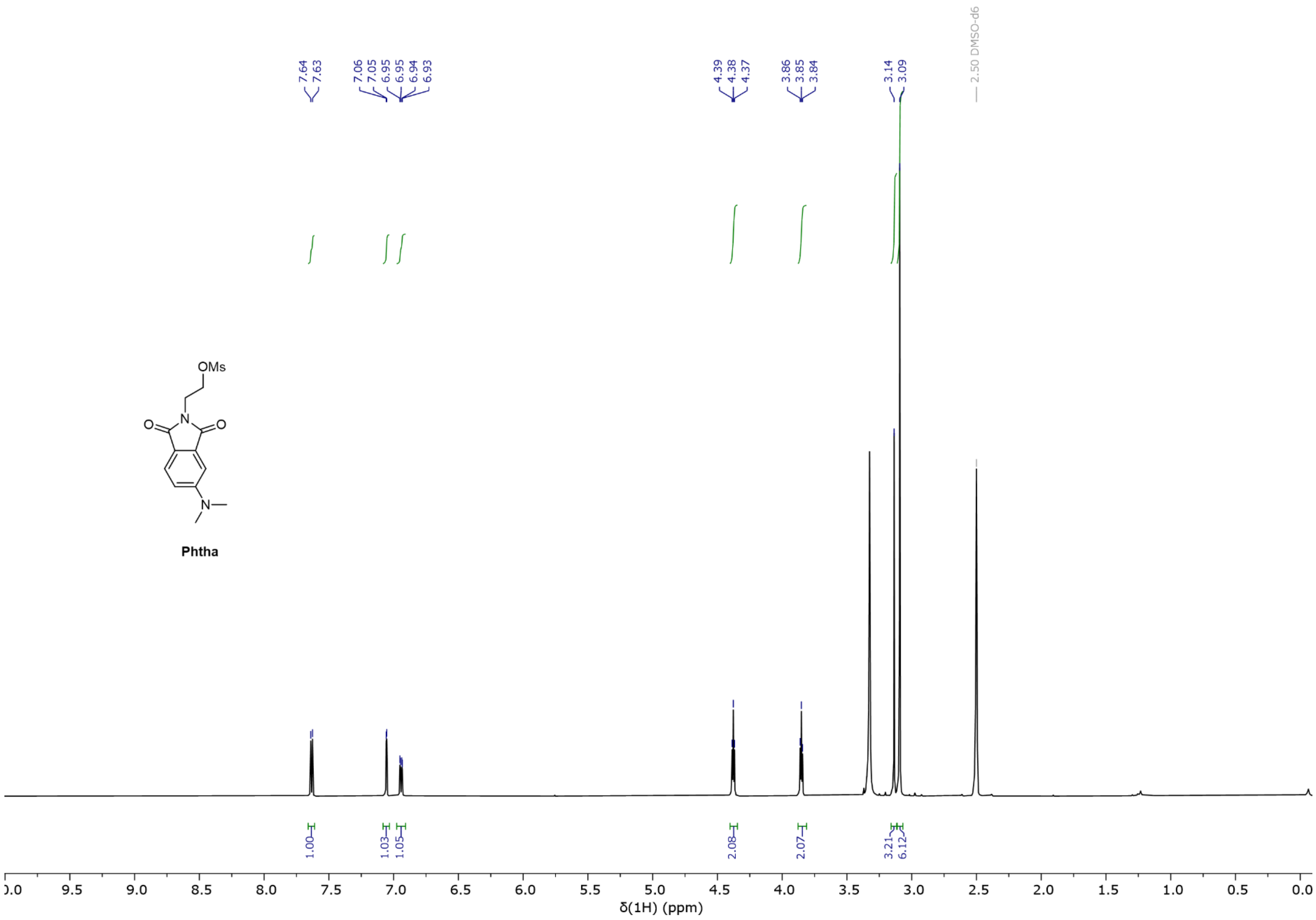

Figure S70. ^{1}H NMR spectrum (600 MHz, DMSO-d_6) of **Phtha**.

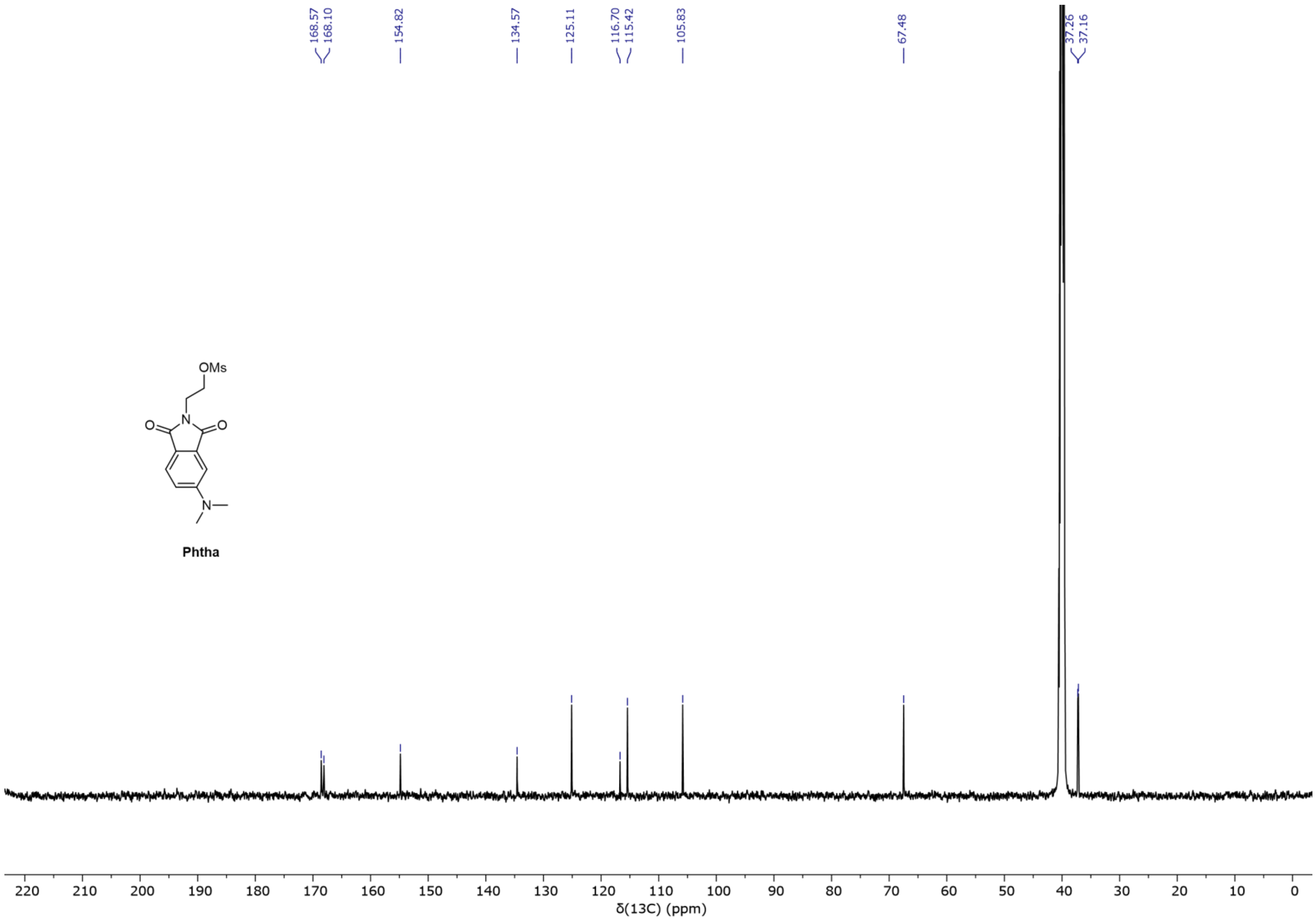

Figure S71. ^{13}C NMR spectrum (151 MHz, DMSO-d_6) of **Phtha**.

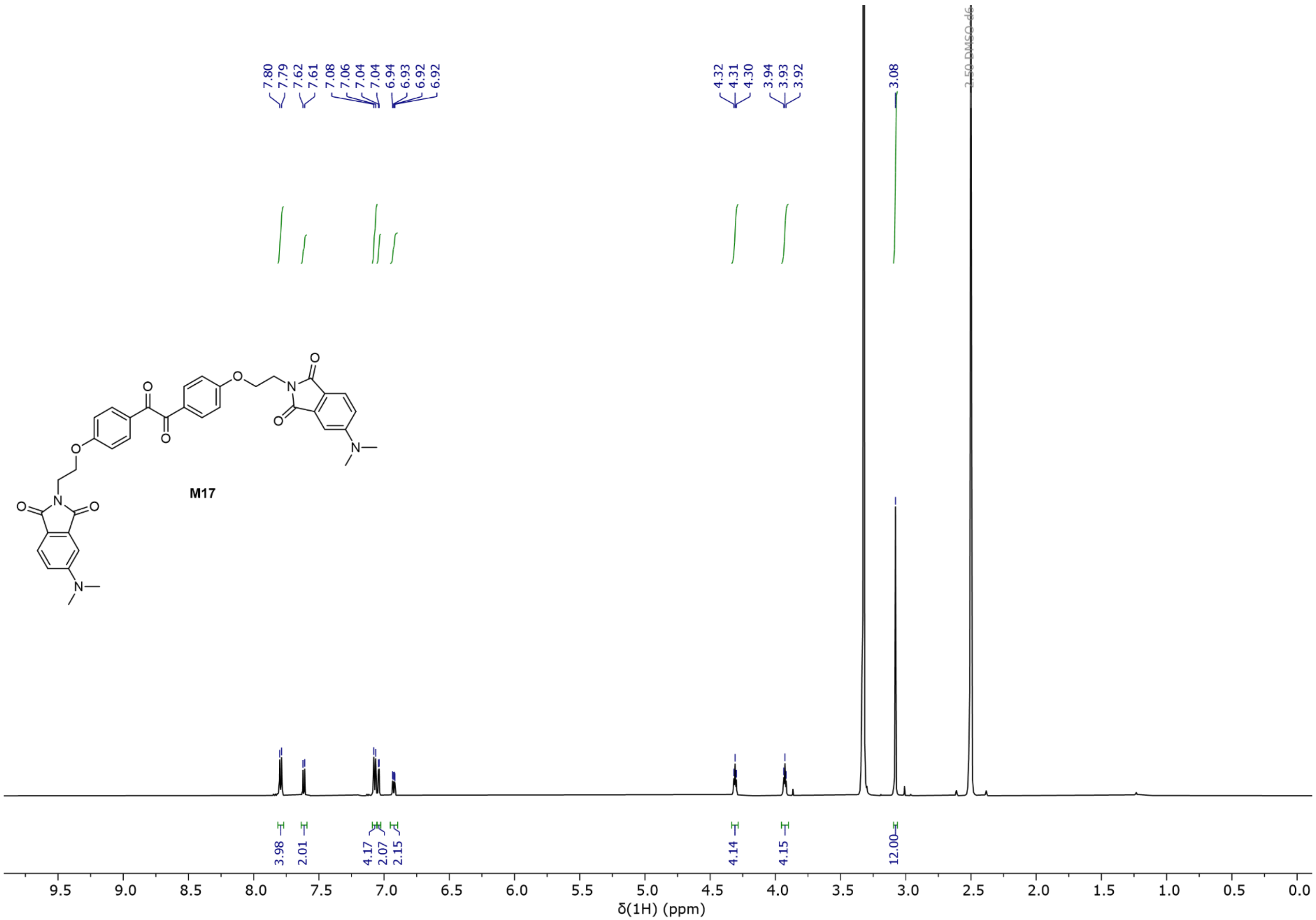

Figure S72. ^{1}H NMR spectrum (600 MHz, DMSO-d_6) of **M17**.

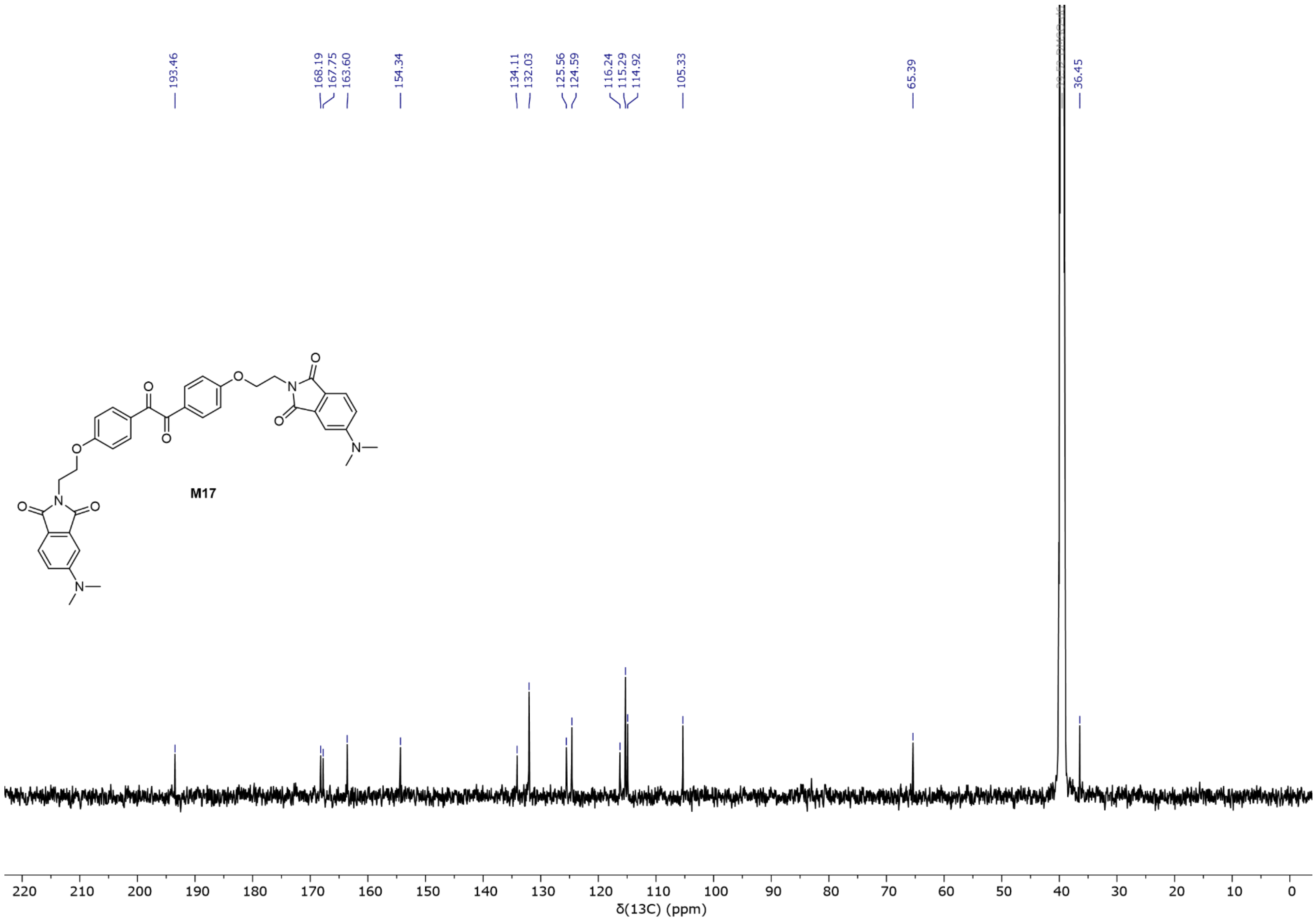

Figure S73. ^{13}C NMR spectrum (151 MHz, DMSO-d_6) of **M17**.

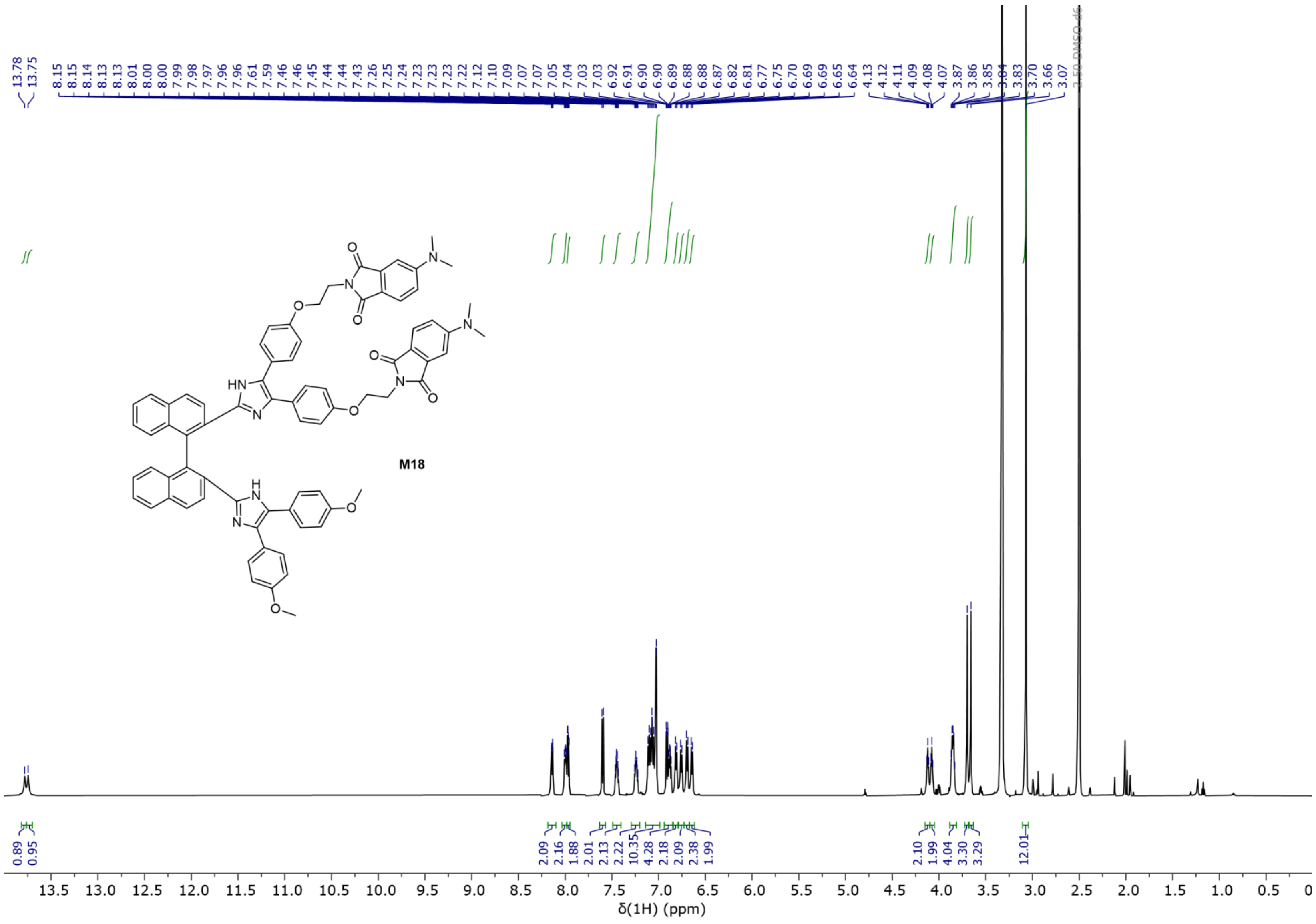

Figure S74. ^{1}H NMR spectrum (600 MHz, DMSO-d_6) of **M18**.

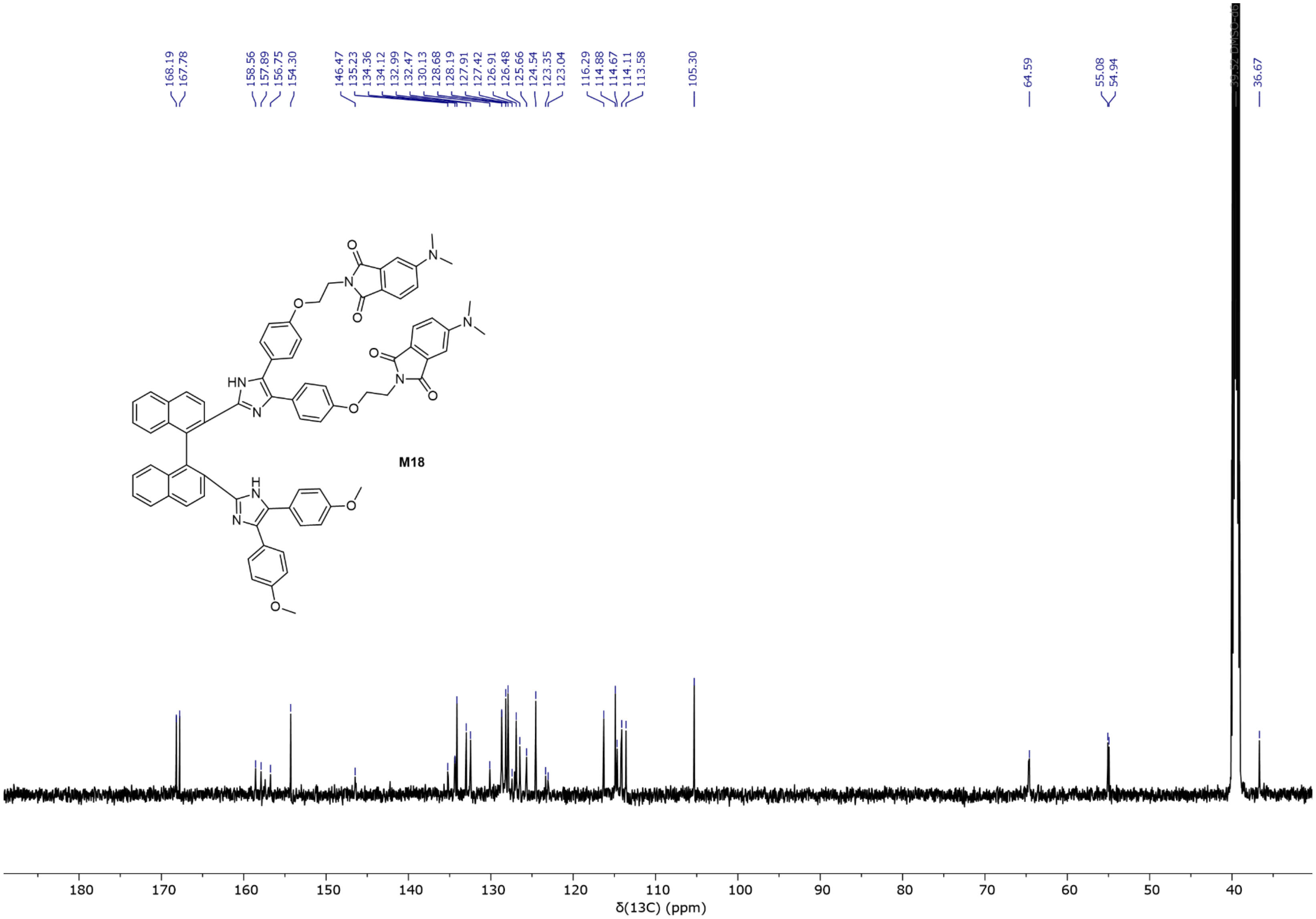

Figure S75. ^{13}C NMR spectrum (151 MHz, DMSO-d_6) of **M18**.

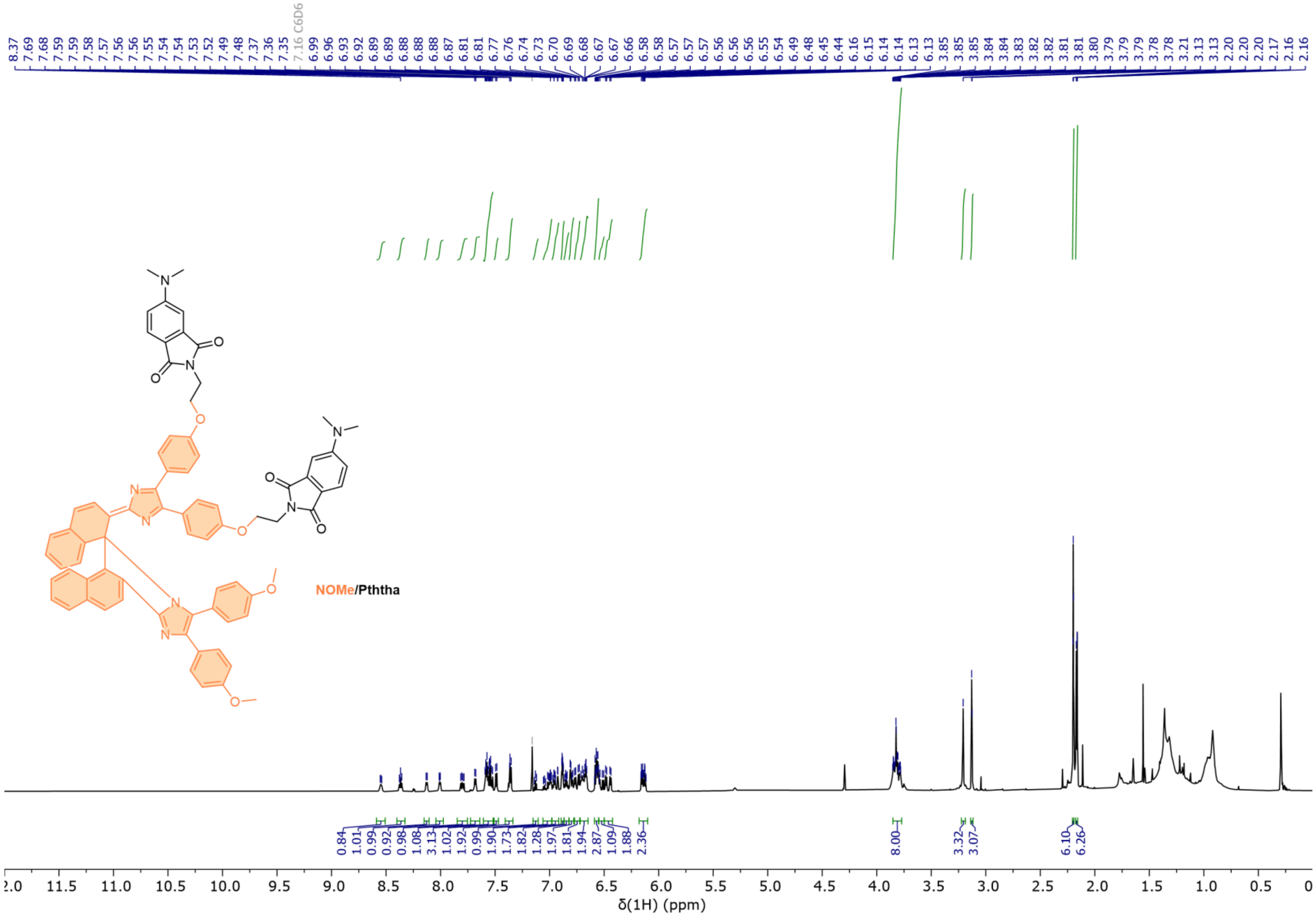

Figure S76. ^{1}H NMR spectrum (800 MHz, C_6D_6) of **NOMe/Phtha**.

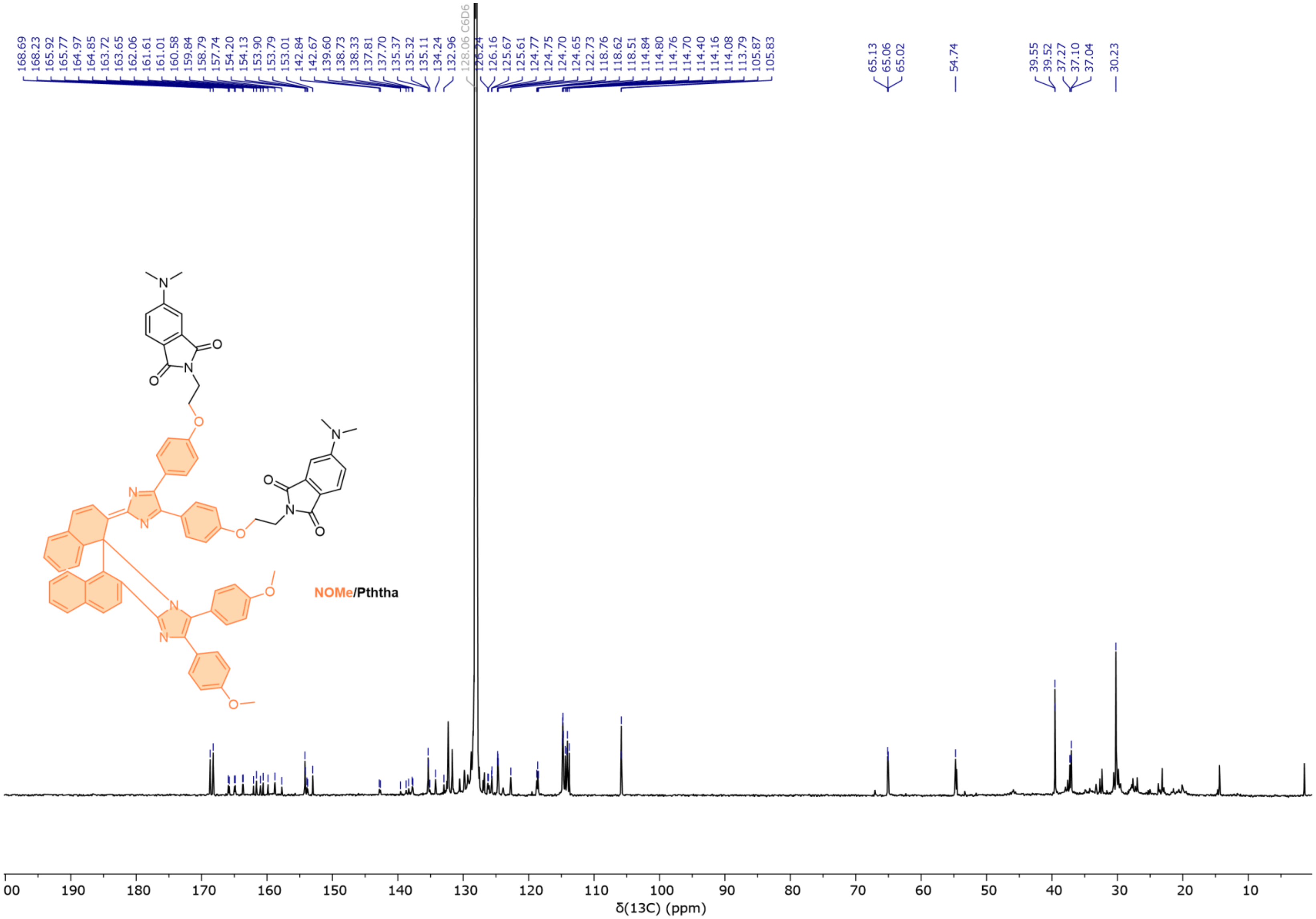

Figure S77. ^{13}C NMR spectrum (201 MHz, C_6D_6) of **NOMe/Phtha**.

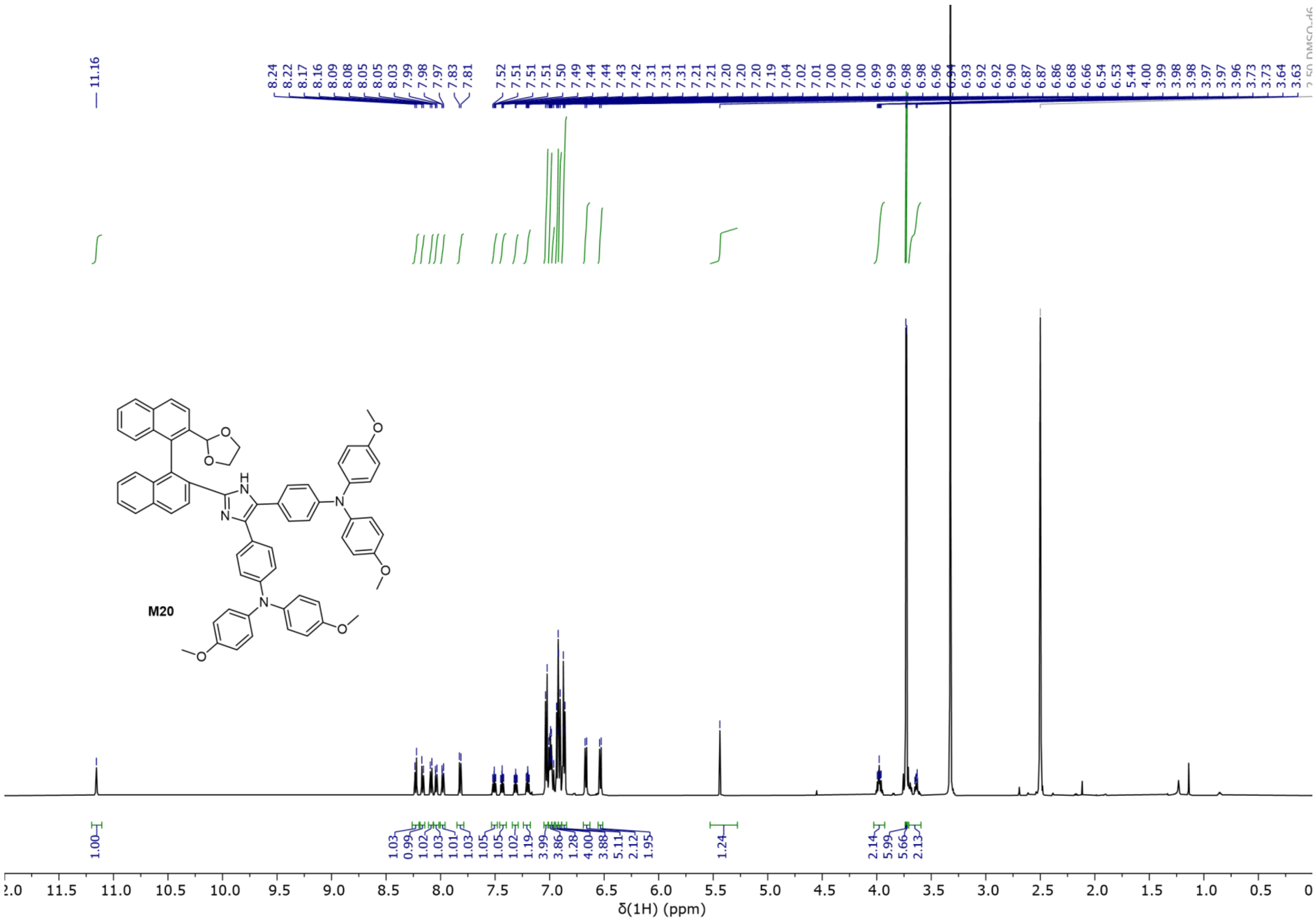

Figure S78. ^{1}H NMR spectrum (600 MHz, DMSO-d_6) of **M20**.

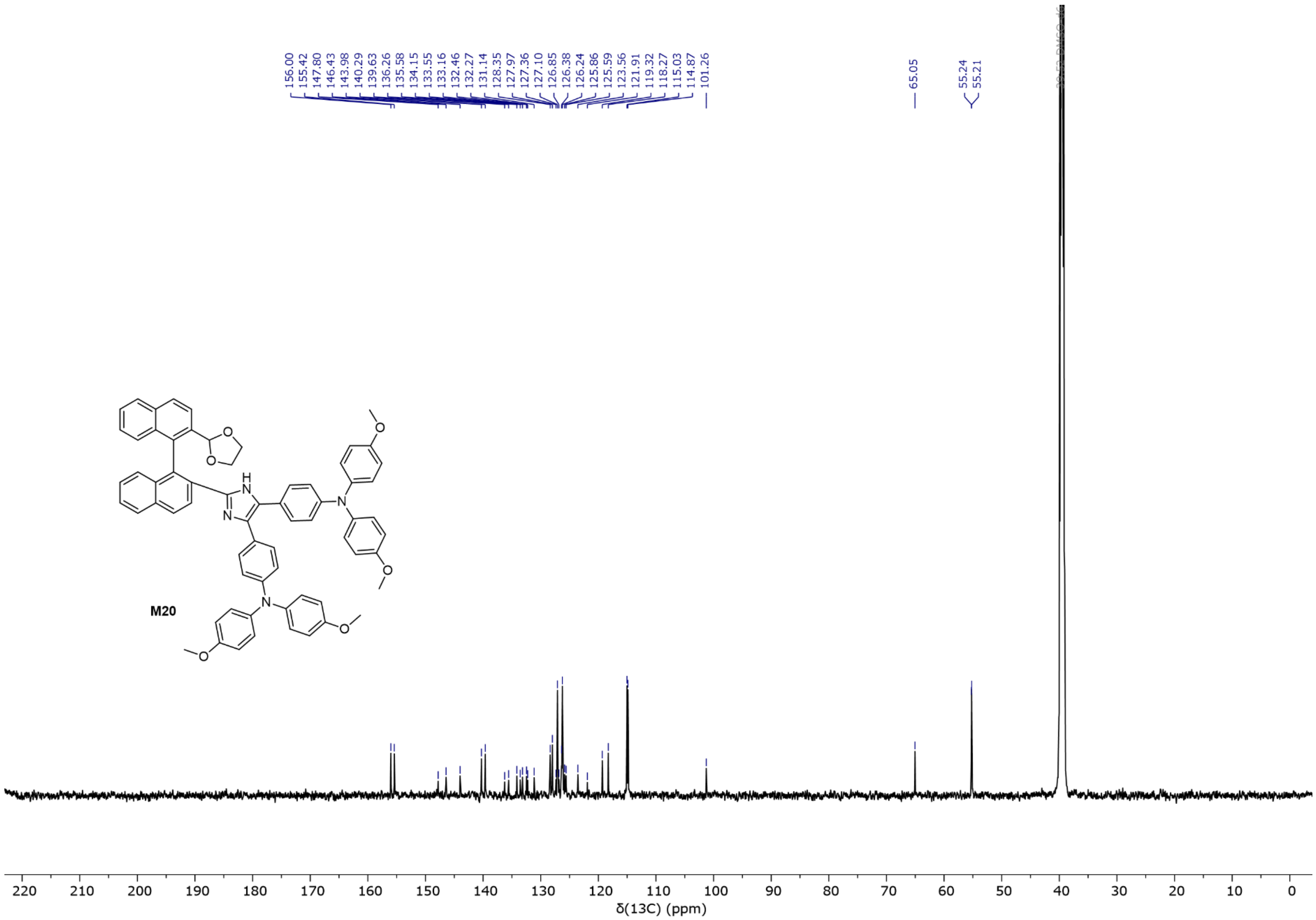

Figure S79. ^{13}C NMR spectrum 151 MHz, DMSO-d_6) of **M20**.

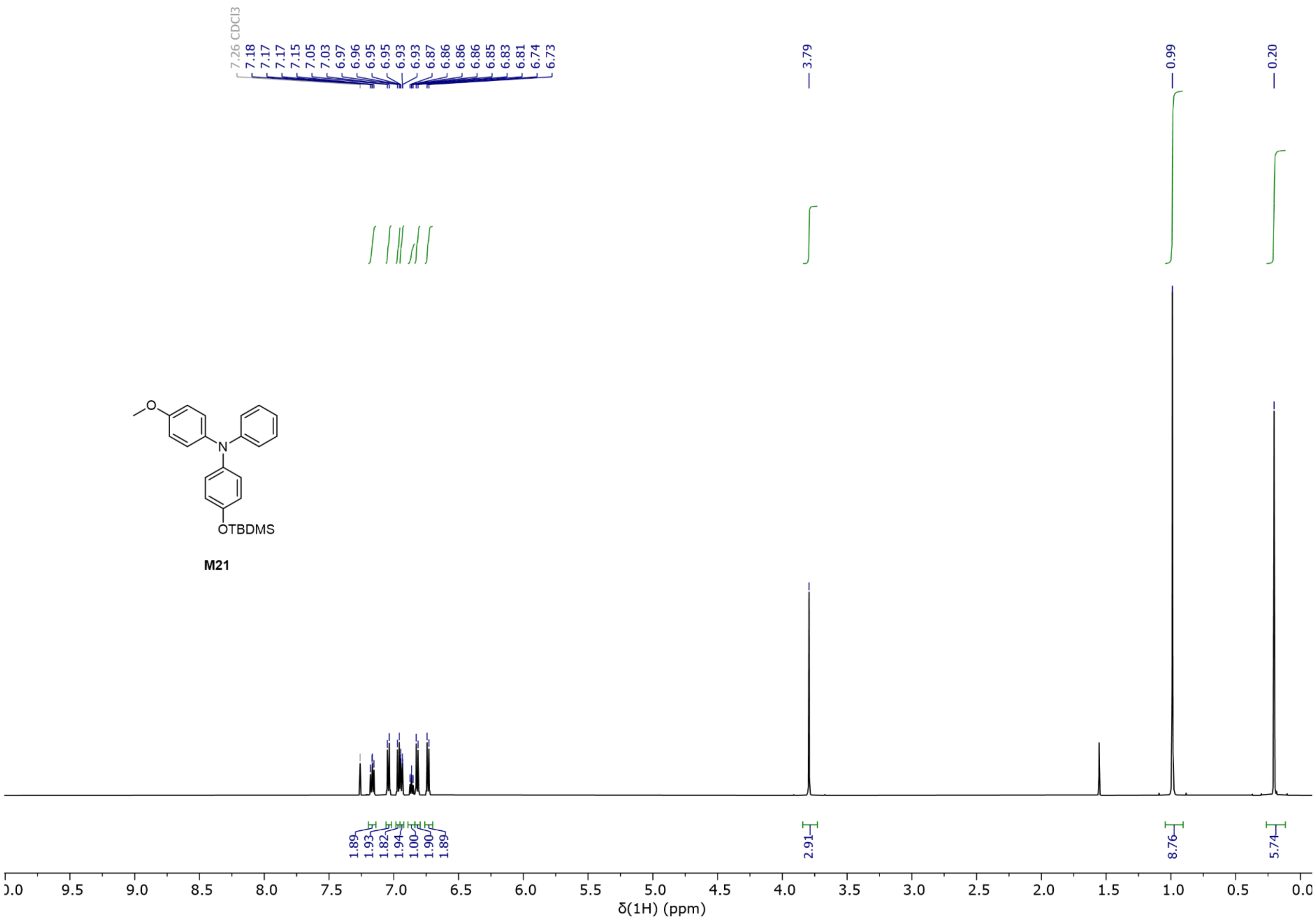

Figure S80. ^{1}H NMR spectrum (600 MHz, $CDCl_3$) of **M21**.

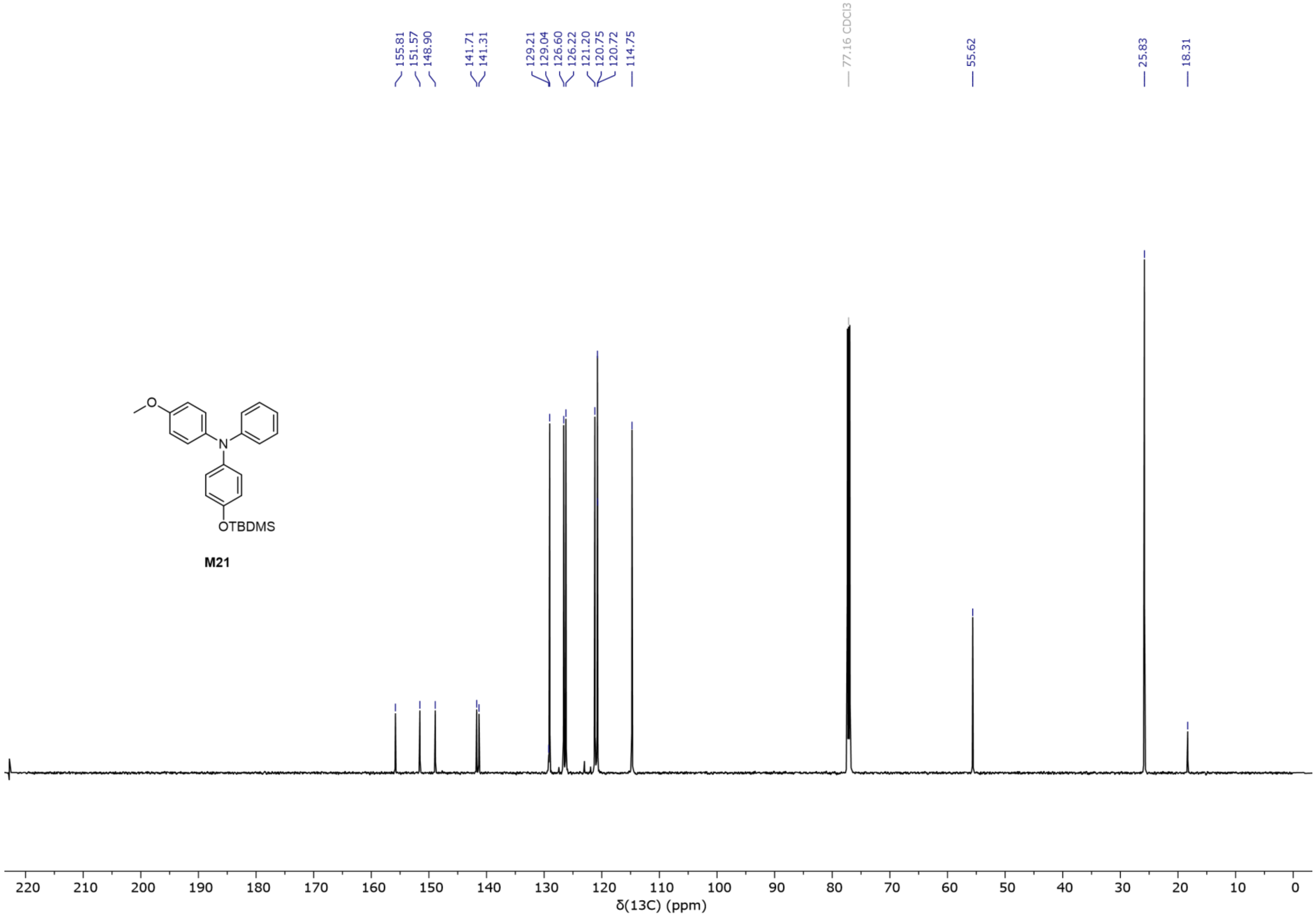

Figure S81. ^{13}C NMR spectrum 151 MHz, $CDCl_3$) of **M21**.

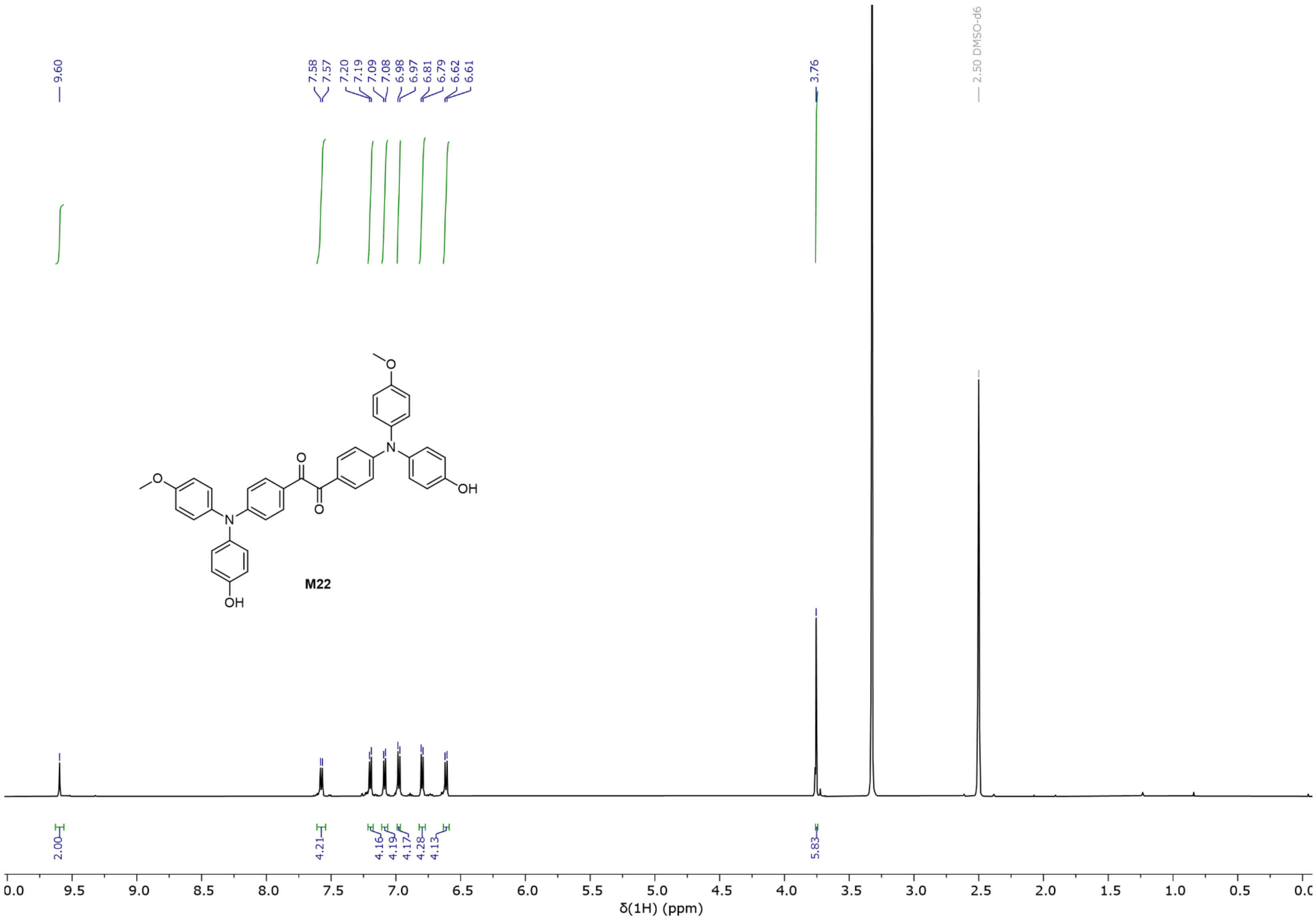

Figure S82. ^{1}H NMR spectrum (600 MHz, $CDCl_3$) of **M22**.

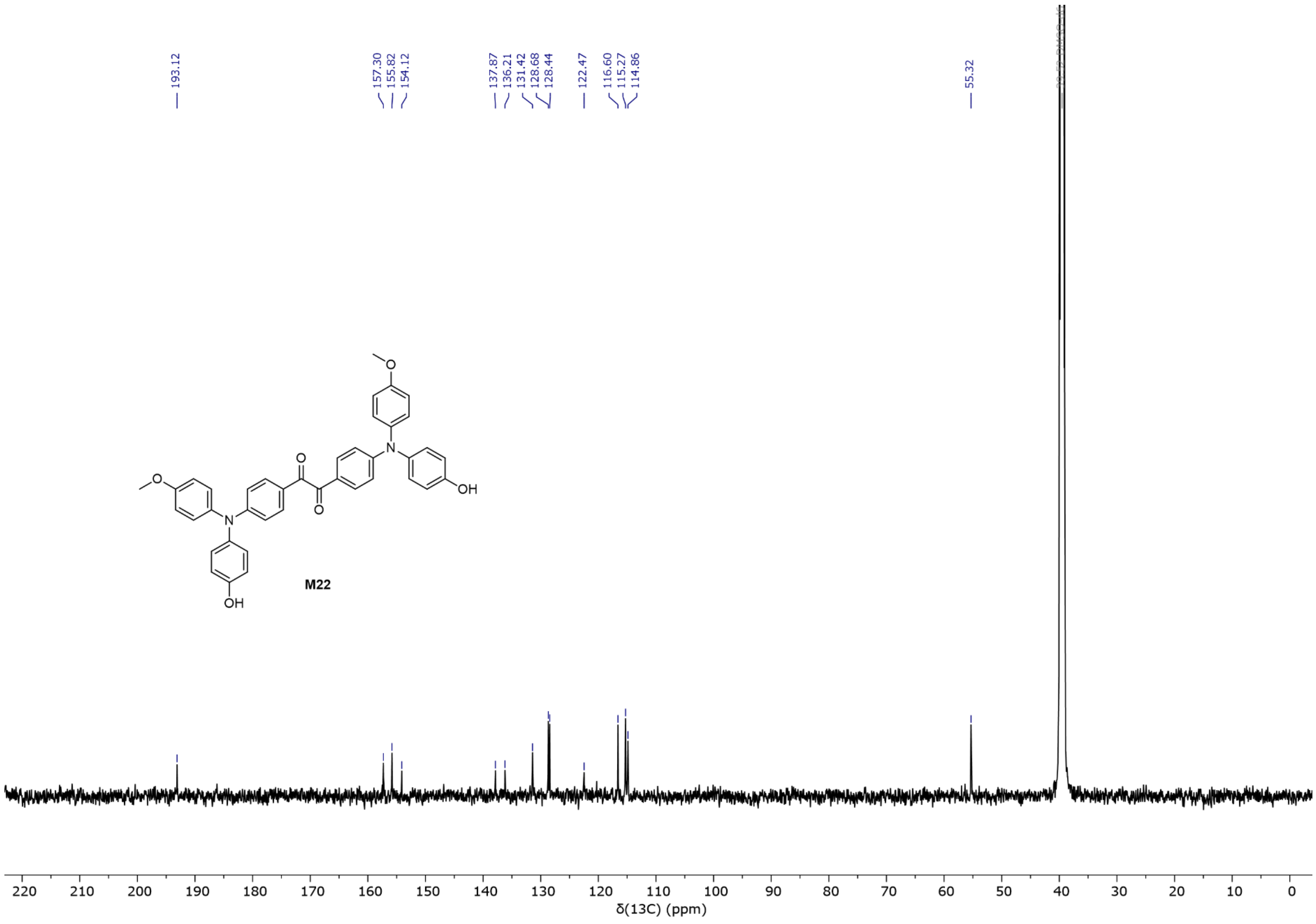

Figure S83. ^{13}C NMR spectrum 151 MHz, $CDCl_3$) of **M22**.

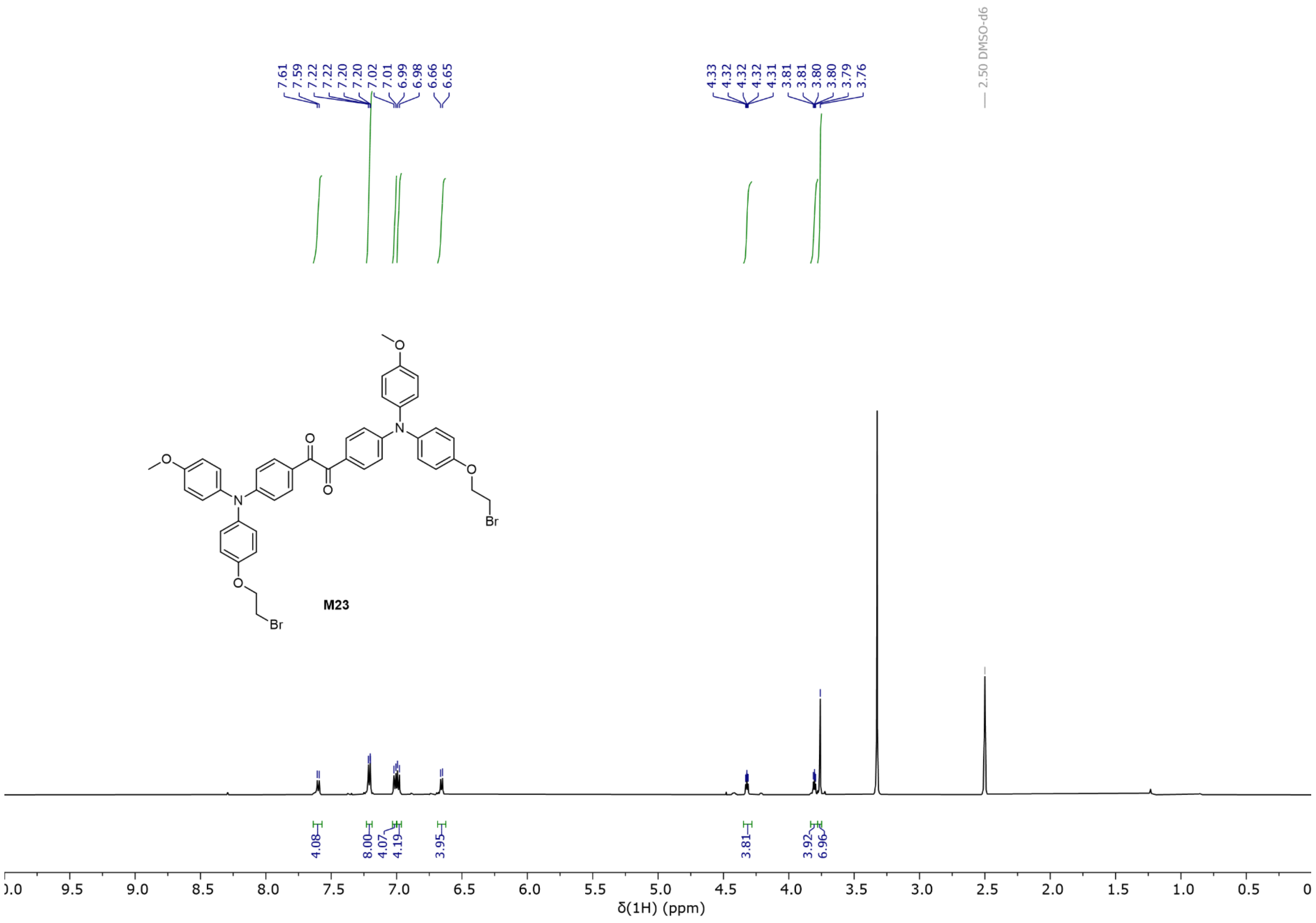

Figure S84. ^{1}H NMR spectrum (600 MHz, DMSO-d_6) of **M23**.

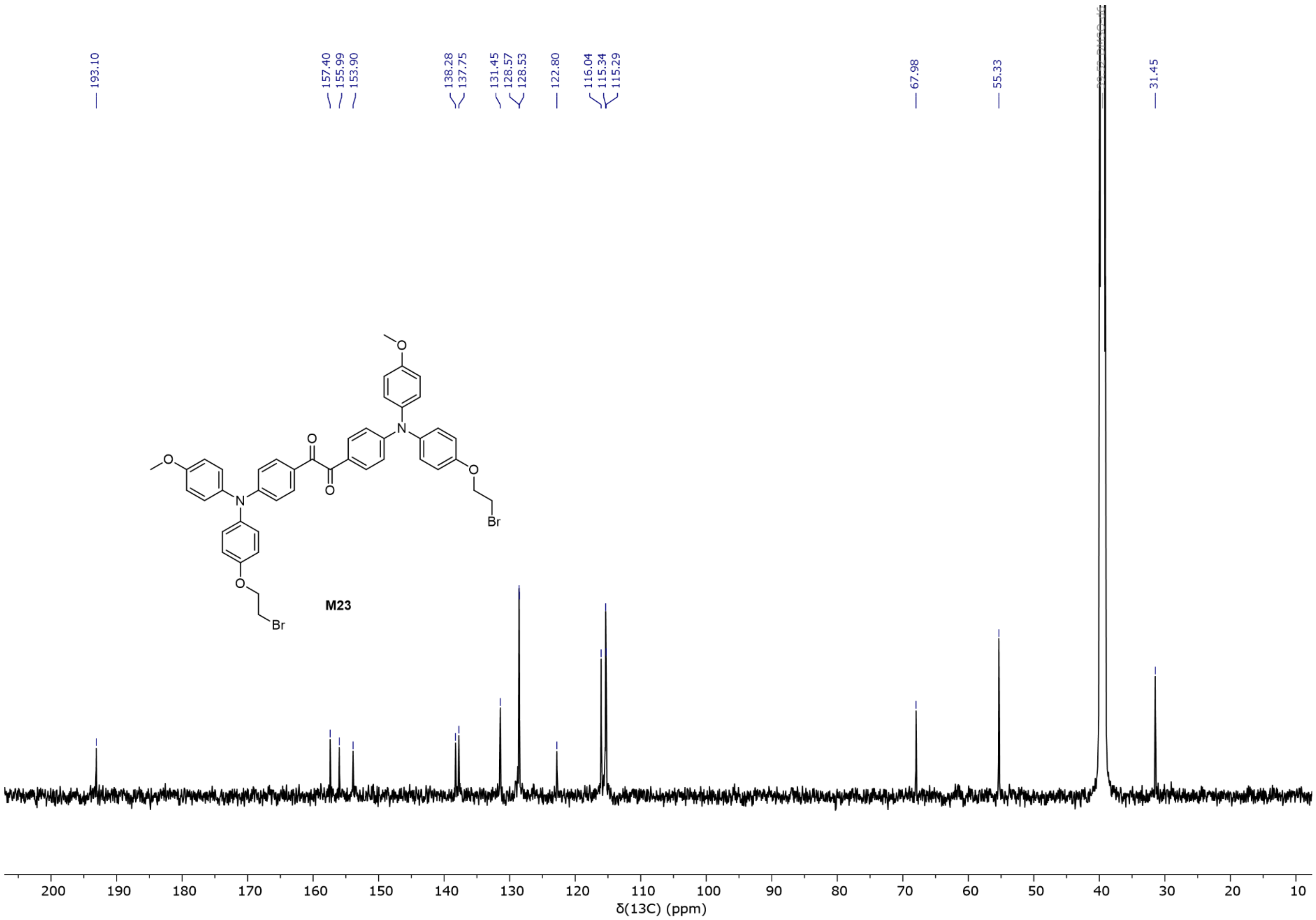

Figure S85. ^{13}C NMR spectrum 151 MHz, DMSO-d_6) of **M23**.

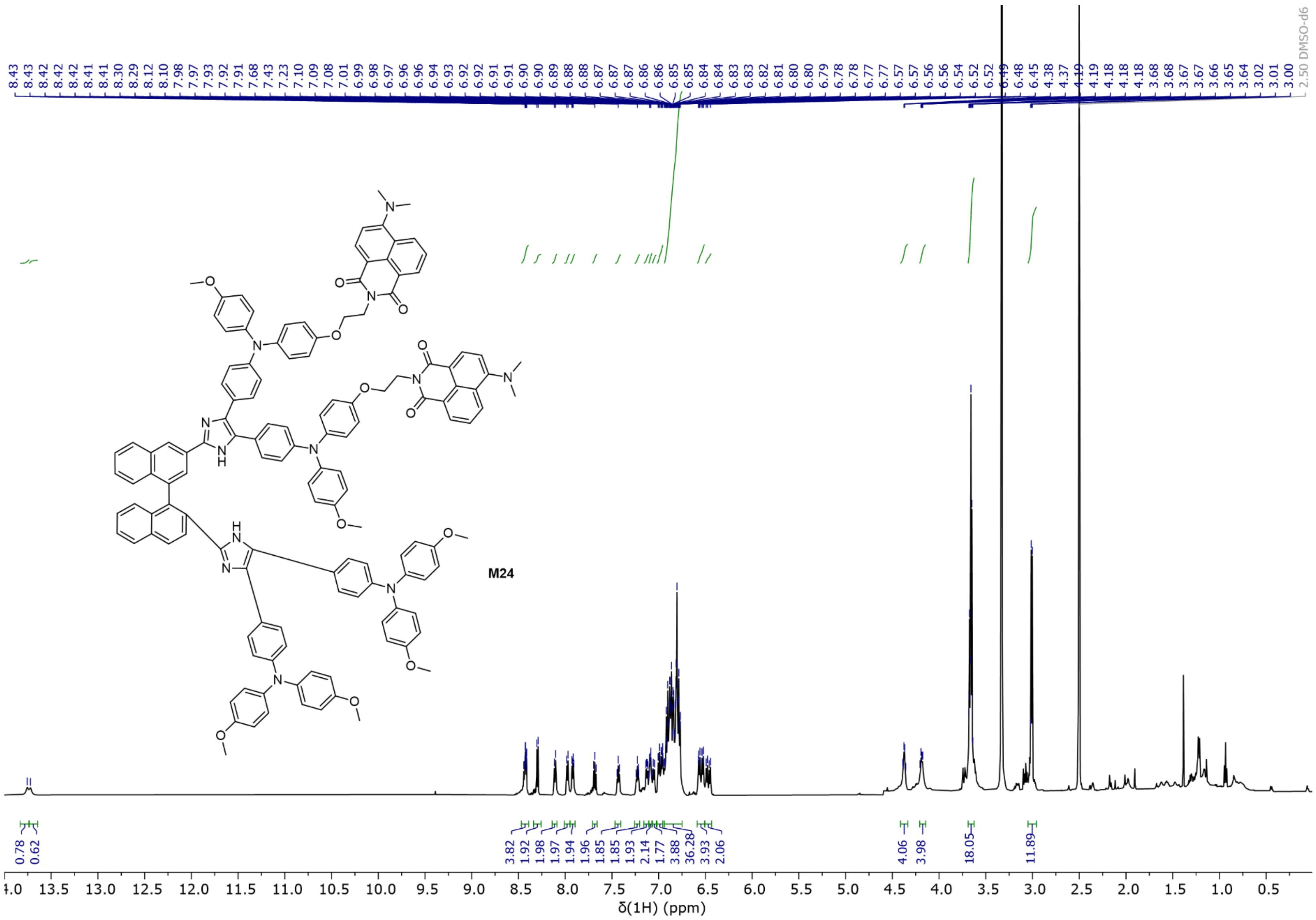

Figure S86. ¹H NMR spectrum (600 MHz, DMSO-d_6) of **M24**.

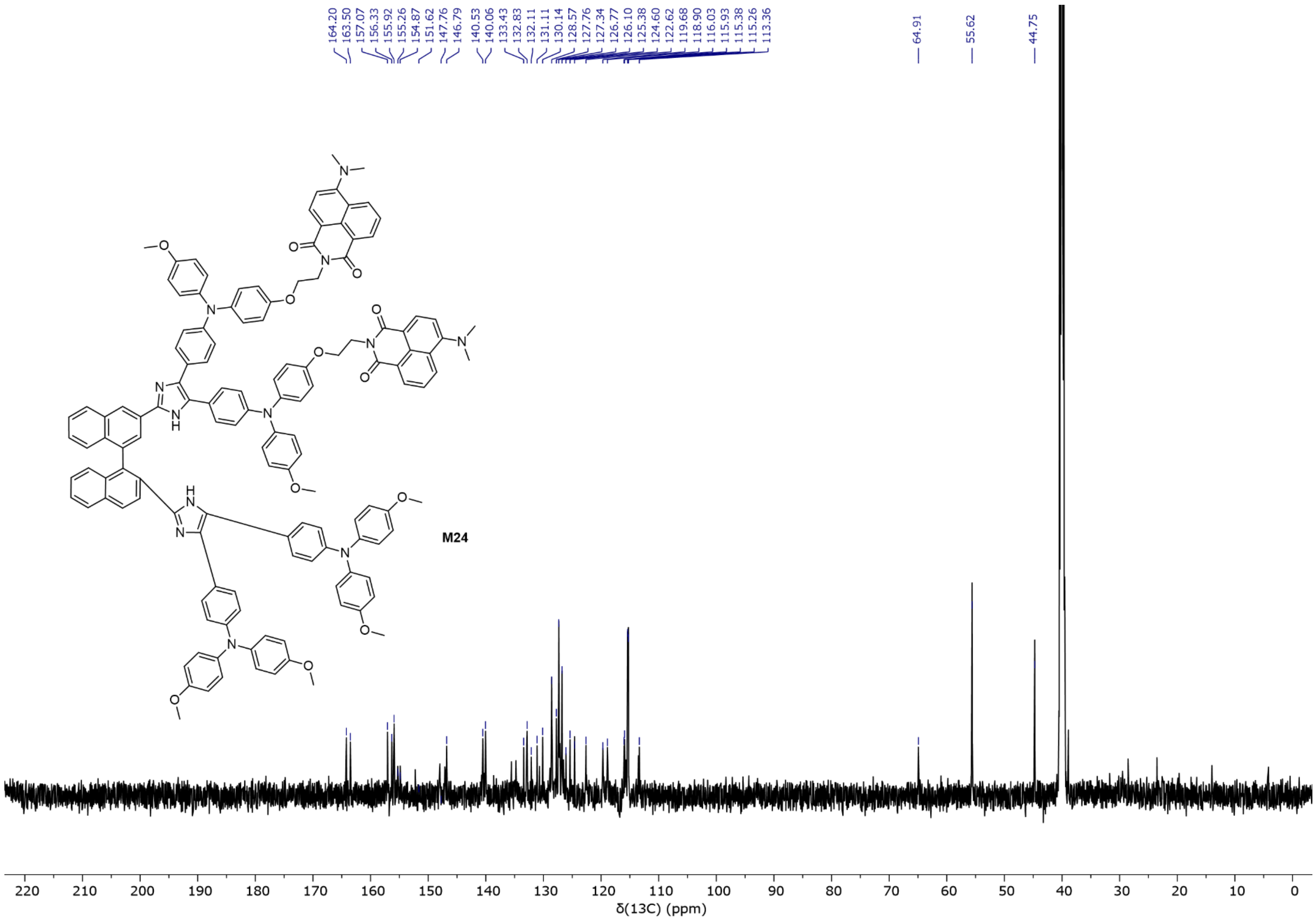

Figure S87. ^{13}C NMR spectrum 151 MHz, DMSO-d_6) of **M24**.

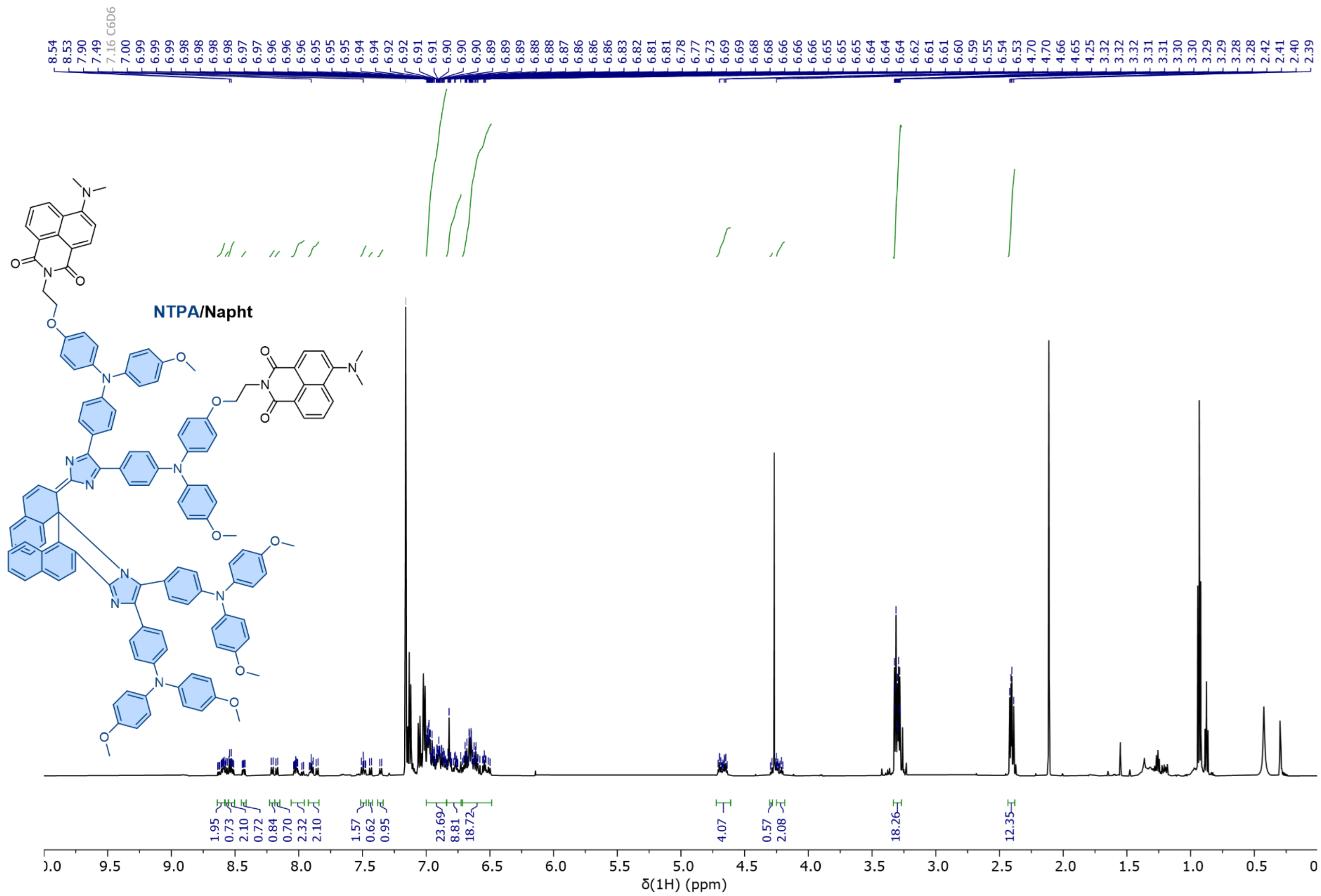

Figure S88. ^{1}H NMR spectrum (600 MHz, C_6D_6) of **NTPA/Napht**.

6. Supplementary Notes

Supplementary Note 1.

The 2PA cross section of a derivative similar to the **DASA** analogue employed in this work is reported to be negligible in reference 29 in the manuscript (i.e., Reza-González, F. A., Villatoro, E., Reza, M. M., Jara-Cortés, J., García-Ortega, H., Blanco-Acuña, E. F., López-Cortés, J. G., Esturau-Escofet, N., Aguirre-Soto, A. & Peon, J. Two-photon isomerization properties of donor-acceptor Stenhouse adducts. *Chem. Sci.* **14**, 5783-5794 (2023)). There are no data available for the 1,1'-binaphtyl-bridged imidazole dimers.

Supplementary Note 2.

The observation of slopes less than 4 can be attributed to background emission, that is, emission occurring before the sensitized photoisomerization produces the fluorescent isomer. The background emission could originate from minor amounts of fluorescent impurities or fluorescent PS_{CL}-2PAP isomers present at thermal equilibrium, or a combination thereof. We exclude non-quantitative FRET-quenching as the origin, as the theoretically estimated and the experimentally observed FRET efficiencies (E_{FRET}) are determined to 0.999 (indicating virtually quantitative quenching).

7. Supplementary References

1. Portier, F., Solier, J. & Halila, S. *N*,*N'*-disubstituted barbituric acid: a versatile and modular multifunctional platform for obtaining *β*-glycoconjugates from unprotected carbohydrates in water. *Eur. J. Org. Chem.* **2019**, 6158-6162 (2019).

2. Rouillon, J., Benitez-Martin, C., Grøtli, M. & Andréasson, J. Click and shift: the effect of triazole on solvatochromic dyes. *Phys. Chem. Chem. Phys.* **27**, 4679-4685 (2025).

3. Mutoh, K., Miyashita, N., Arai, K. & Abe, J. Turn-on mode fluorescence switch by using negative photochromic imidazole dimer. *J. Am. Chem. Soc.* **141**, 5650-5654 (2019).

4. Wang, T., Wu, Y., Kuan, S. L., Dumele, O., Lamla, M., Ng, D. Y., Arzt, M., Thomas, J., Mueller, J. O., Barner-Kowollik, C. & Weil, T. A disulfide intercalator toolbox for the site-directed modification of polypeptides. *Chem. Eur. J.* **21**, 228-238 (2015).

5. Costales, P., Rios-Lombardia, N., Lorenzo-Herrero, S., Moris, F. & Gonzalez-Sabin, J. Novel chiral naphthalimide-cycloalkanediamine conjugates: design, synthesis and antitumor activity. *Bioorg. Chem.* **112**, 104859 (2021).

6. Sainlos, M. & Imperiali, B. Synthesis of anhydride precursors of the environment-sensitive fluorophores 4-DMAP and 6-DMN. *Nat. Protoc.* **2**, 3219-3225 (2007).

7. Kometani, A., Inagaki, Y., Mutoh, K. & Abe, J. Red or near-infrared light operating negative photochromism of a binaphthyl-bridged imidazole dimer. *J. Am. Chem. Soc.* **142**, 7995-8005 (2020).

8. Banthia, S., Samanta, A. Influence of structure on the unusual spectral behavior of 4-dialkylamino-1,8-naphthalimide. *Chem. Lett.*, **34**, 722-723 (2005).

9. Hatano, S., Horino, T., Tokita, A., Oshima, T. & Abe, J. Unusual negative photochromism via a short-lived imidazolyl radical of 1,1'-binaphthyl-bridged imidazole dimer. *J. Am. Chem. Soc.* **135**, 3164-3172 (2013).

10. Benitez-Martin, C., Li, S., Dominguez-Alfaro, A., Najera, F., Perez-Inestrosa, E., Pischel, U. & Andréasson, J. Toward two-photon absorbing dyes with unusually potentiated nonlinear fluorescence response. *J. Am. Chem. Soc.* **142**, 14854-14858 (2020).

11. Agam, G., Gebhardt, C., Popara, M., MSchtel, R., Folz, J., Ambrose, B., Chamachi, N., Chung, S. Y., Craggs, T. D., de Boer, M., Grohmann, D., Ha, T., Hartmann, A., Hendrix, J., Hirschfeld, V., Hübner, C. G., Hugel, T., Kammerer, D., Kang, H. S., Kapanidis, A. N., Krainer, G., Kramm, K., Lemke, E. A., Lerner, E., Margeat, E., Martens, K., Michaelis, J., Mitra, J., Muñoz, G. G. M., Quast, R. B., Robb, N. C., Sattler, M., Schlierf, M., Schneider, J., Schröder, T., Sefer, A., Tan, P. S., Thurn, J., Tinnefeld, P., van Noort, J., Weiss, S., Wendler, N., Zijlstra, N., Barth, A., Seidel, C. A. M., Lamb, D. C. & Cordes, T. Reliability and accuracy of single-molecule FRET studies for characterization of structural dynamics and distances in proteins. *Nat. Methods* **20**, 523-535 (2023).
