# Supplementary material for: Exploiting negative photochromism to harness a four-photon-like fluorescence response with two-photon excitation
Source: Nat Commun. 2025 Dec 3;16:10897. doi: 10.1038/s41467-025-66602-1 (PMC12678773; doi:10.1038/s41467-025-66602-1)
Supplement: Supplementary file 2 — Description of Additional Supplementary Files [file 41467_2025_66602_MOESM2_ESM.pdf]

Description of Additional Supplementary Files

Supplementary Data 1: Atomic coordinates of DASA/FRT

Supplementary Data 2: Atomic coordinates of DASA/Napht

Supplementary Data 3: Atomic coordinates of NOMe/Napht

Supplementary Data 4: Atomic coordinates of asyNOMe/Napht

Supplementary Data 5: Atomic coordinates of NOMe/Phtha

Supplementary Data 6: Atomic coordinates of NTPA/Napht)
